# Transcribed ultraconserved region 339 promotes carcinogenesis by modulating tumor suppressor microRNAs

**DOI:** 10.1038/s41467-017-01562-9

**Published:** 2017-11-27

**Authors:** Ivan Vannini, Petra M. Wise, Kishore B. Challagundla, Meropi Plousiou, Mirco Raffini, Erika Bandini, Francesca Fanini, Giorgia Paliaga, Melissa Crawford, Manuela Ferracin, Cristina Ivan, Linda Fabris, Ramana V. Davuluri, Zhiyi Guo, Maria Angelica Cortez, Xinna Zhang, Lu Chen, Shuxing Zhang, Cecilia Fernandez-Cymering, Leng Han, Silvia Carloni, Samanta Salvi, Hui Ling, Mariam Murtadha, Paolo Neviani, Barbara J. Gitlitz, Ite A. Laird-Offringa, Patrick Nana-Sinkam, Massimo Negrini, Han Liang, Dino Amadori, Amelia Cimmino, George A. Calin, Muller Fabbri

**Affiliations:** 1 Istituto Scientifico Romagnolo per lo Studio e la Cura dei Tumori (IRST) S.r.l., IRCCS, Gene Therapy Unit, 47014 Meldola (FC), Italy; 2 Departments of Pediatrics and Molecular Microbiology & Immunology, Norris Comprehensive Cancer Center, Keck School of Medicine, University of Southern California, Children’s Center for Cancer and Blood Diseases and The Saban Research Institute, Children’s Hospital Los Angeles, Los Angeles, CA 90027 USA; 3 Department of Biochemistry and Molecular Biology, University of Nebraska Medical Center, 985870 Nebraska Medical Center, Omaha, NE 68198 USA; 4 Department of Internal Medicine, Division of Pulmonary, Allergy, Critical Care and Sleep Medicine, The Ohio State University, Columbus, OH 43210 USA; 5 Department of Experimental, Diagnostic and Specialty Medicine—DIMES, University of Bologna, 40126 Bologna, Italy; 6 Department of Experimental Therapeutics, The University of Texas MD Anderson Cancer Center, Houston, TX 77030 USA; 7 The Center for RNA Interference and Non-coding RNAs, The University of Texas MD Anderson Cancer Center, Houston, 77030 TX USA; 8 Departments of Preventive Medicine and Neurological Surgery, Northwestern University-Feinberg School of Medicine, Chicago, IL 60611 USA; 9 Department of Experimental Radiation Oncology, The University of Texas MD Anderson Cancer Center, Houston, TX 77030 USA; 10 Department of Gynecologic Oncology and Reproductive Medicine, The University of Texas MD Anderson Cancer Center, Houston, TX 77030 USA; 11 Integrated Molecular Discovery Laboratory, Department of Experimental Therapeutics, The University of Texas MD Anderson Cancer Center, Houston, TX 77030 USA; 12 Department of Molecular Virology, Immunology and Medical Genetics, Comprehensive Cancer Center, Ohio State University, Columbus, OH 43210 USA; 13 Department of Biochemistry and Molecular Biology, McGovern Medical School, The University of Texas Health Science Center at Houston, Houston, TX 77030 USA; 14 Istituto Scientifico Romagnolo per lo Studio e la Cura dei Tumori (IRST) S.r.l., IRCCS, Biosciences Laboratory Unit, 47014 Meldola (FC), Italy; 15 Division of Medical Oncology, Norris Comprehensive Cancer Center, Keck School of Medicine, University of Southern California, Los Angeles, CA 90033 USA; 16 Departments of Surgery and Biochemistry and Molecular Medicine, Norris Comprehensive Cancer Center, Keck School of Medicine, University of Southern California, Los Angeles, CA 90033 USA; 17 Division of Pulmonary Diseases and Critical Care Medicine, Virginia Commonwealth University, Richmond, VA 23298 USA; 18 Department of Morphology, Surgery and Experimental Medicine and Laboratory for Technologies of Advanced Therapies (LTTA), University of Ferrara, 44121 Ferrara, Italy; 19 Department of Bioinformatics and Computational Biology, The University of Texas MD Anderson Cancer Center, Houston, TX 77030 USA; 20 Department of Oncology Unit, Istituto Scientifico Romagnolo per lo Studio e la Cura dei Tumori (IRST) S.r.l., IRCCS, 47014 Meldola (FC), Italy; 21 Institute of Genetics and Biophysics, National Research Council, 80131 Naples, Italy; 22 Present Address: Division of Pre-Clinical Innovation, National Center for Advancing Translational Sciences (NCATS), Rockville, MD 20850 USA

## Abstract

The transcribed ultraconserved regions (T-UCRs) encode long non-coding RNAs implicated in human carcinogenesis. Their mechanisms of action and the factors regulating their expression in cancers are poorly understood. Here we show that high expression of *uc.339* correlates with lower survival in 210 non-small cell lung cancer (NSCLC) patients. We provide evidence from cell lines and primary samples that TP53 directly regulates *uc.339*. We find that transcribed *uc.339* is upregulated in archival NSCLC samples, functioning as a decoy RNA for *miR-339-3p*, *-663b-3p*, and *-95-5p*. As a result, Cyclin E2, a direct target of all these microRNAs is upregulated, promoting cancer growth and migration. Finally, we find that modulation of *uc.339* affects microRNA expression. However, overexpression or downregulation of these microRNAs causes no significant variations in *uc.339* levels, suggesting a type of interaction for *uc.339* that we call “entrapping”. Our results support a key role for *uc.339* in lung cancer.

## Abstract (web summary)

T-UCRs encode long non-coding RNAs implicated in human carcinogenesis, but the underlying mechanisms are poorly understood. Here, the authors identify *uc.339* as an oncogene in lung cancer that is upregulated through the loss of TP53 and promotes Cyclin E activation by entrapping regulatory miRNAs.

## Introduction

Cancer is a complex genetic disease driven by dysregulation not only of protein-coding genes, but also of non-coding RNAs (ncRNAs). Among the latter group, microRNAs (miRNAs) are the most widely studied, but other families of ncRNAs are emerging as involved in human carcinogenesis. The ultraconserved regions (UCRs) are a family of genomic sequences longer than 200 base pairs (bp) with 100% identity between orthologous regions of the human, murine, and rat genomes^1^. We previously identified their transcriptional activity and named these as transcribed UCRs (T-UCR) genes^2^. Most T-UCRs are not translated into proteins. They are frequently located at fragile sites and other cancer-associated genomic regions and are dysregulated in several types of solid and hematological malignancies, compared to their normal tissue counterpart^2^. An altered expression of T-UCRs has been observed in pediatric tumors^3, 4^ and is correlated with outcome in high-risk neuroblastomas^3^. We described that *uc.73A* has oncogenic activity in colorectal cancer (CRC) cell lines, whereas other groups identified *uc.73A* and *uc.338* as oncogenes in CRC^5^ and hepatocellular carcinoma (HCC)^6^, respectively. Also, single nucleotide polymorphisms (SNPs) in T-UCR genes have been found to be associated with familial breast cancer risk^7^. Our group previously showed that T-UCR genes are epigenetically regulated by miRNAs^2^ and CpG island promoter hypermethylation^8^. Recently, it was shown that transcribed *uc.339* is overexpressed in HCC cells and HCC-derived exosomes, contributing to a pro-tumoral HCC microenvironment^9^. Also, we recently showed that *uc.8* is able to promote bladder carcinogenesis by interacting with *miR-596* ^10^. Collectively, these findings support a role for T-UCRs in human carcinogenesis. However, much about the mechanism and consequences of dysregulation of T-UCR in human cancers remains unknown.

In this study we examine whether *uc.339* is differentially expressed in non-small cell lung cancer (NSCLC) primary tumors compared to that in the adjacent non-cancerous lung tissue, and we assess in The Cancer Genome Atlas (TCGA) database whether this expression correlates with overall survival. Then, we determine whether *uc.339* acts as an oncogene in lung cancer, and investigate a possible mechanism of action of the encoded *uc.339* RNA. Finally, we test whether *TP53*, a tumor-suppressor gene deleted or mutated in more than 50% of human tumors, including NSCLC^11–13^, might be responsible for *uc.339* dysregulation in lung cancer. We discovered that transcribed *uc.339* is directly regulated by TP53, is significantly upregulated in NSCLCs, and interacts with multiple Cyclin E2 regulating miRNAs by a type of interaction that we call “entrapping”. Our results support a key role for *uc.339* in lung cancer.

## Results

### *uc.339* is upregulated in NSCLC tumors with poor prognosis

**Fig. 1:**
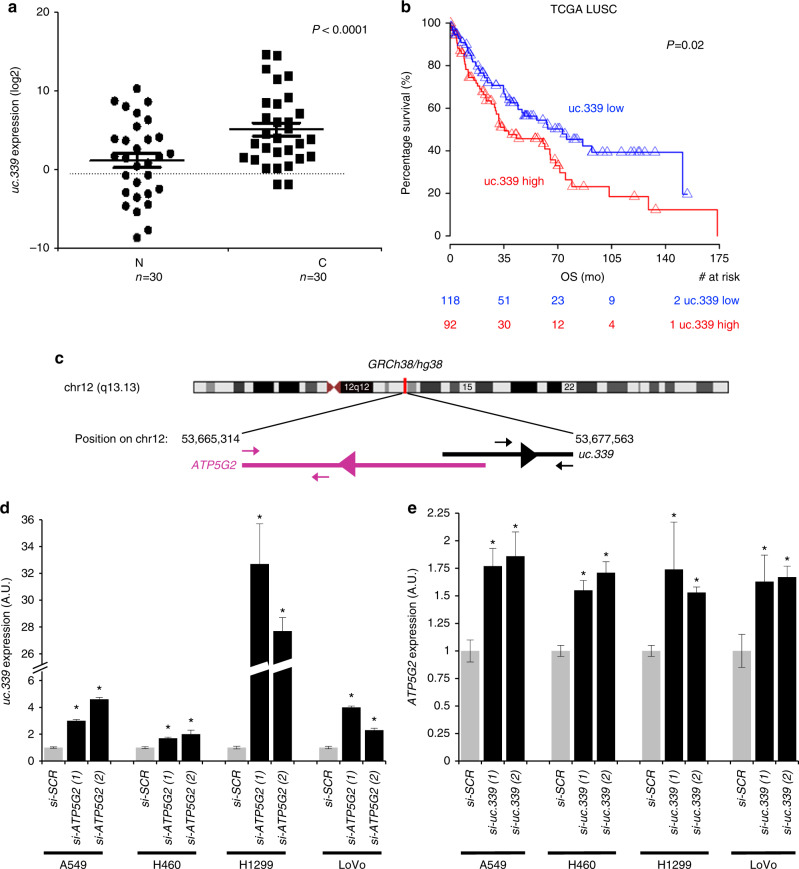
*uc.339* expression in NSCLC and its inverse correlation with *ATP5G2* gene. **a** qRT-PCR for *uc.339* in 30 paired primary NSCLC cancerous tissues (C) and the adjacent non-tumor lung (N). The expression of *uc.339* has been normalized to *RNU44* and log2 transformed. Data are shown as mean ± s.d. referred to N mean value. The linear fold-change between C and N was 20.6. Paired *t*-test *P*-value <0.0001. **b** Kaplan–Meier survival curve of 210 patients with lung squamocellular carcinoma (LUSC) from the TCGA database, expressing high (red) or low (blue) levels of *uc.339*. OS overall survival, mo months, TCGA The Cancer Genome Atlas. **c** Map of *uc.339* on chromosome 12q13.13 and its location in relation to its host gene *ATP5G2* (according to the GRCh38/hg38 Human Genome Chromosome information). The black arrowheads above and below the bar representing the *uc.339* transcript indicate the position of the primers used in this study for the detection of *uc.339* by qRT-PCR, while the violet arrowheads above and below the bar representing the *ATP5G2* indicate the position of the primers used for the detection of *ATP5G2* mRNA by qRT-PCR. The transcriptional direction of the *ATP5G2* gene and of the *uc.339* gene are indicated by the purple and black arrowhead within the bars, respectively. **d** qRT-PCR for *uc.339* in A549, H460, H1299, and LoVo cells transfected with two different anti-*ATP5G2* siRNAs (si-*ATP5G2 (1)* and *(2))* or an anti-scrambled siRNA (si-*SCR*). The expression of *uc.339* has been normalized to *RNU44* and presented as normalized to si-SCR *P < 0.05. **e** qRT-PCR for *ATP5G2* in A549, H460, H1299, and LoVo cells transfected with two different anti-*uc.339* siRNAs (si-*uc.339 (1)* and *(2)*) or an anti-scrambled siRNA (si-*SCR*). The expression of *ATP5G2* has been normalized to *HPRT1* and presented as normalized to si-*SCR*. *P < 0.05. All data are presented as mean ± s.d. of experiments conducted in triplicate

Increased expression of *uc.339* has been described in CRC and HCC, compared to that in the corresponding non-tumor tissue^2, 9^. We asked whether such an upregulation also occurred in NSCLC primary samples. We measured *uc.339* RNA in 30 paired frozen tumor and adjacent non-tumor lung, by quantitative real-time PCR (qRT-PCR). Statistically significant upregulation of *uc.339* was observed in tumor compared to that in the adjacent non-tumor lung (*P* < 0.0001 Fig. 1a). To assess whether the increased *uc.339* RNA levels harbored prognostic implications, we interrogated RNA-seq data from 210 NSCLC patients from the TCGA database and found that high levels of *uc.339* significantly associate with a lower overall survival (*P* = 0.02, log-rank, Fig. 1b).

### *uc.339* promotes NSCLC growth and migration

As we observed higher levels of *uc.339* RNA in NSCLC primary tissues compared to that in the adjacent non-tumor lung, we investigated the functional implications of *uc.339* dysregulation in lung carcinogenesis. According to the classification of UCRs as devised by Bejerano et al.^1^, *uc.339* is a possibly exonic T-UCR (meaning a T-UCR with inconclusive evidence of overlap with a protein-coding gene), partly embedded in the *ATP5G2* host gene (Fig. 1c). We determined the length of the *uc.339* transcript by rapid amplification of cDNA ends (RACE), and we assessed the endogenous expression of *uc.339* in three NSCLC cell lines (A549, H1299, and H460) and one CRC cell line (LoVo) by QuantStudio 3D digital PCR (dPCR), carefully selecting for primers designed to amplify within the ultraconserved area as described by Bejerano et al.^1^, but not overlapping with the *ATP5G2* host gene (Fig. 1c). The tested cell lines showed a variable expression of *uc.339*, with the highest expression in H1299 and LoVo, as measured by dPCR (Supplementary Fig. 1a, b). Next, we assessed the *uc.339* transcript length by RACE in H1299 and LoVo cells and identified a prevalent *uc.339* RNA of 849 nucleotides (nt), beginning 273 nt upstream and ending 324 nt downstream the sequence, reported by Bejerano et al.^1^, and in antisense direction from its “host” gene *ATP5G2* (Supplementary Fig. 1c). To assess whether *uc.339* and *ATP5G2* transcripts affect each other’s expression, we transfected the four different cell lines (A549, H460, H1299, and LoVo) with siRNA anti-*ATP5G2* (Fig. 1d) or with siRNA anti-*uc.339* (Fig. 1e) and observed upregulation of *uc.339* and *ATP5G2* transcripts, respectively. This suggests that *uc.339* might function as an antisense for the *ATP5G2* transcript.

**Fig. 2:**
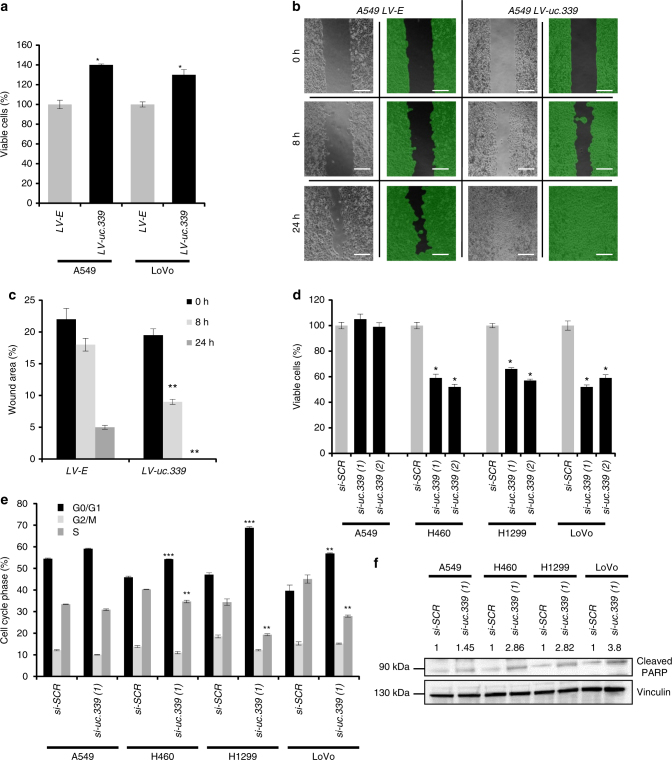
*uc.339* promotes cancer cell growth and migration. **a** Cell viability assay in A549 and LoVo cells infected with a lentiviral vector overexpressing *uc.339* (LV-*uc.339*) or its empty vector counterpart (LV-E) and detected after 72 h. Data are presented as mean ± s.d. of experiments conducted in triplicate and normalized to LV-E. *P < 0.05. **b** Representative image of a scratch migration assay in A549 LV-E and A549 LV-*uc.339* cells at 0, 8, and 24 h. Scale bar = 200 μm. **c** Quantification of the percentage of the wound area of the scratch migration assay shown in **b** and represented as mean ± s.d. of experiments conducted in triplicate. **P < 0.01. **d** Cell viability assay in A549, H460, H1299, and LoVo cells transfected with two different si-*uc.339* or si-*SCR* for 72 h. Data are presented as mean ± s.d. of experiments conducted in triplicate and normalized to si-*SCR*. *P < 0.05. **e** Cell-cycle analysis (shown as the percentage of cells in G_0_/G_1_ or G_2_/M or S-phase of the cell cycle) conducted by cytofluorimetry with propidium iodide staining in A549, H460, H1299, and LoVo cells transfected with si-*uc.339(1)* or si-*SCR* for 72 h. Data are presented as mean ± s.d. of experiments conducted in triplicate. **P < 0.01. ***P < 0.001. **f** Immunoblotting for cleaved PARP and Vinculin in A549, H460, H1299, and LoVo cells transfected with si-*uc.339(1)* or si-*SCR* for 72 h. The numbers above the bands represent the quantification of the band intensity, calculated with Quantity One software and normalized to Vinculin and si-SCR

**Fig. 3:**
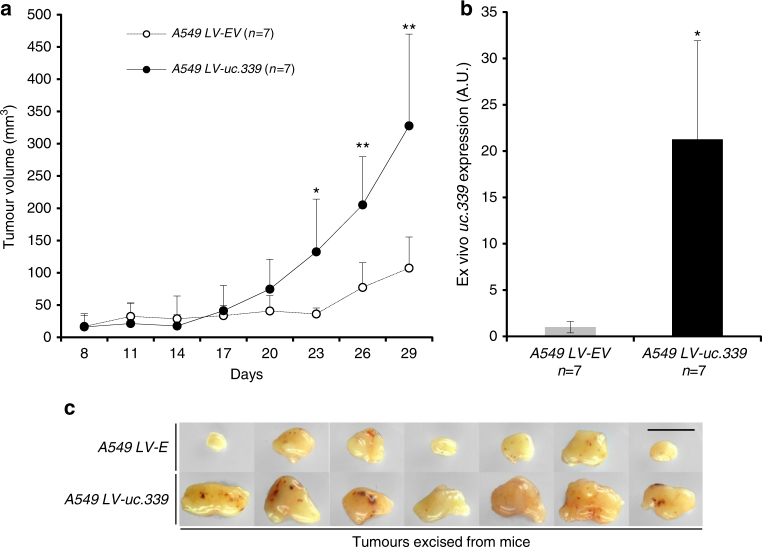
*uc.339* increases NSCLC growth in an in vivo xenograft murine model. **a** Tumor volume growth curve in 14 nude mice injected subcutaneously with A549 LV-E (*n* = 7) or A549 LV-*uc.339* (*n* = 7) and measured from day 8 from the injection until day 29, every 3 days. Data are presented as the mean tumor volumes + s.d. for each animal group. *P < 0.05. **P < 0.01. **b** qRT-PCR for *uc.339* in ex vivo xenografts from the same mice of experiment (**a**). The expression of *uc.339* has been normalized to *RNU44* and data are presented as mean ± s.d. of experiments conducted in triplicate per each mouse, normalized to A549 LV-EV. *P < 0.05. **c** Images of the 14 excised tumors from the mice of experiments (**a**, **b**). Scale bar = 1 cm

Next, we infected A549 and LoVo cells with a lentiviral vector overexpressing the 849 nt *uc.339* RNA (LV-*uc.339*) or its empty vector counterpart (LV-E). We observed that LV-*uc.339*-infected cells (Supplementary Fig. 2a) have an increased viability at 72 h (Fig. 2a) and increased migration in the scratch assay after 24 h (Fig. 2b, c) compared to that in the LV-E infected cells. Conversely, when we silenced *uc.339* endogenous expression with two different siRNAs against two different regions of the *uc.339* transcript [si-*uc.339*(1) and si-*uc.339*(2)] in A549, H460, H1299, and LoVo cells (Supplementary Fig. 2b), we observed reduction of cell viability at 72 h in H460, H1299, and LoVo, but not in A549 (Fig. 2d). Finally, we assessed the effects of *uc.339* silencing in A549, H460, H1299, and LoVo cells by cytofluorimetry. We observed that a decreased expression of *uc.339* reduced the percentage of cells in S-phase, increased the percentage of cells in G_0_/G_1_ phase, and induced protein PARP-cleavage (Fig. 2e, f; Supplementary Fig. 2c, d), an indication of cellular apoptosis. Again, these effects were absent or less pronounced in A549 cells, which express the lowest levels of *uc.339* (Supplementary Fig. 1a), supporting the *uc.339*-mediated effect on cell viability and cell cycle. Next, we assessed the effects of *uc.339* overexpression in an in vivo xenograft murine model. Five-week-old nude female mice (*n* = 7 per group) were injected subcutaneously with A549 LV-*uc.339* or LV-E infected cells. Tumor growth was measured every 3 days from the moment in which the tumors became measurable and until day 29 in both groups. Mice injected with *uc.339* overexpressing A549 cells showed a faster tumor growth than mice injected with control A549 cells, and the difference between the two groups became statistically significant from day 23 (Fig. 3a). After killing the mice, ex vivo analysis of *uc.339* expression in the xenografts showed a significantly persistent higher expression of *uc.339* in the LV-*uc.339* group (Fig. 3b) and bigger tumor volumes in the LV-*uc.339* group (Fig. 3c).

### Effects of *uc.339* on miRNA expression

**Fig. 4:**
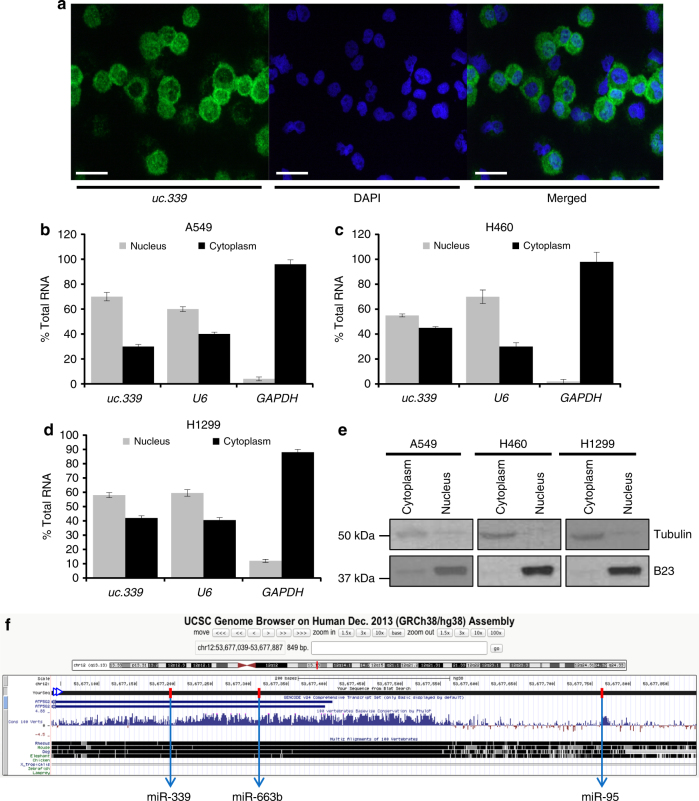
*uc.339* is located both in the nucleus and in the cytoplasm of cancer cells. **a** Cytospin fluorescent in situ hybridization of the endogenous levels of the *uc.339* transcript (green) in H1299 cells. DAPI blue fluorescent co-staining was used to show nuclear labeling. Both stainings can be seen in the “Merged” images. Scale bar = 50 μm. **b**–**d** qRT-PCR for *uc.339*, *RNAU6*, and *GAPDH* in the nuclear and cytoplasmatic cellular fractions and expressed as the percentage of total cellular RNA in A549 (**b**), H460 (**c**), and H1299 (**d**) cell lines. Data are presented as mean ± s.d. of experiments conducted in triplicate. **e** Immunoblotting for Tubulin and B23 in the same nuclear and cytoplasmic protein cellular fractions of A549, H460, and H1299 cells from which RNA was also extracted for the experiments in **b**–**d**. **f** Genome Browser annotation of *uc.339* locus showing the direction of the transcript, all annotated *ATP5G2* regions, the sequence conservation and the positions of the regions that interact with each of the miRNAs (red bars)

**Fig. 5:**
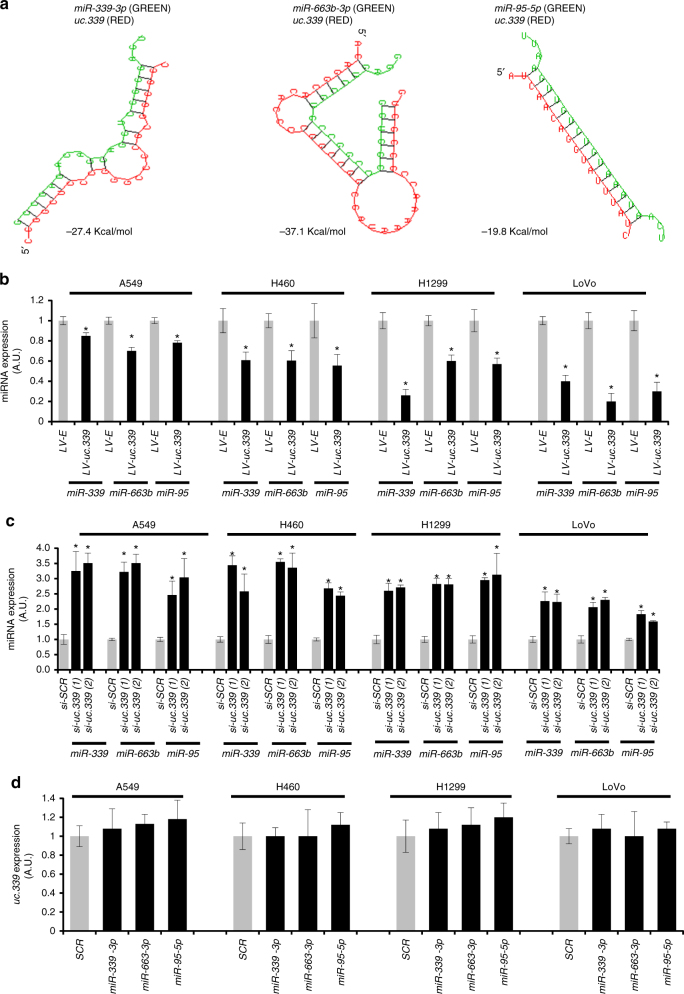
*uc.339* is a decoy for miRNAs. **a** In silico prediction of the interaction between *uc.339* (in red) and *miR-339*, *-663b*, and *-95* (green). **b** qRT-PCR for *miR-339*, *-663b*, and *-95* in A549, H460, H1299, and LoVo cells infected with a lentiviral vector overexpressing *uc.339* (LV-*uc.339*) or its empty vector counterpart (LV-E) and detected after 72 h. The expression of miRNAs has been normalized to *RNU44* RNA and the results are presented as normalized to LV-E. Data are shown as mean ± s.d. of experiments conducted in triplicate. *P < 0.05. (**c**) qRT-PCR for *miR-339*, *-663b*, and *-95* in A549, H460, H1299, and LoVo cells transfected with two different siRNAs anti-*uc.339* (si-*uc.339(1)* and si*-uc.339(2)*) or a siRNA anti-scrambled (si-*SCR*) as a control, for 72 h. The expression of miRNAs has been normalized to *RNU44* RNA and the results are presented as normalized to si-*SCR*. Data are shown as mean ± s.d. of experiments conducted in triplicate. *P < 0.05. **d** qRT-PCR for *uc.339* in A549, H460, H1299, and LoVo cells transfected with *miR-339*, *-663b*, and *-95* or a scrambled miRNA (SCR) after 48 h from transfection. The expression of *uc.339* has been normalized to *RNU44* RNA and presented as normalized to SCR. Data are presented as mean ± s.d. of experiments conducted in triplicate

**Fig. 6:**
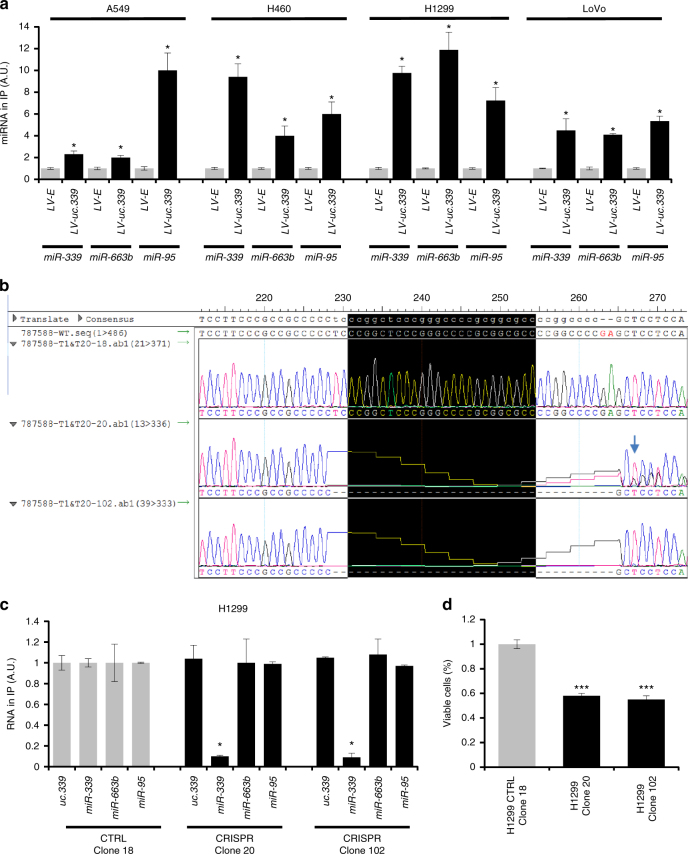
Endogenous *uc.339* sequesters miRNAs. **a** qRT-PCR for *miR-339*, *-663b*, and *-95* in A549, H460, H1299, and LoVo cells infected with LV-*uc.339* or LV-E, in which *uc.339* has been immunoprecipitated. The expression of miRNAs is presented as normalized to the LV-E group. Data are shown as mean ± s.d. of experiments conducted in triplicate. *P < 0.05. **b** Sequencing of clones #18, #20 and #102 in H1299 cells engineered with CRISPR/Cas9 genomic technology for the deletion of the binding site of *miR-339* (nucleotides inside the black box) in the sequence of *uc.339*. **c** qRT-PCR for *uc.339*, *miR-339*, *-663b*, and *-95* in the immunoprecipitate of *uc.339* in H1299 cells wild-type (clone 18) or in clones #20 and #102 of H1299 in which the MBE for *miR-339* has been deleted with CRISPR/Cas9 technology. The expression of the RNAs is presented as normalized to the clone 18 group. Data are shown as mean ± s.d. of experiments conducted in triplicate. *P < 0.05. **d** Cell viability assay in H1299 cells expressing wild-type *uc.339* (H1299 CTRL Clone 18) or in CRISPR clones 20 and 102 after 72 h in culture. Data are presented as mean ± s.d. of experiments conducted in triplicate and normalized to H1299 wt. *P < 0.05

To investigate the mechanism of action of *uc.339*, we hypothesized that the T-UCR might work as a “decoy” for complementary mature miRNAs. First, we confirmed that in addition to the nucleus, the endogenous *uc.339* RNA is also in the cytoplasm of H1299 cells (which express high levels of *uc.339*) by cytospin in situ hybridization (Fig. 4a). We also conducted nuclear and cytoplasmic fractionation of untreated A549, H460, and H1299 cells, and showed that the endogenous *uc.339* transcript is also present in the cytoplasm of these NSCLC cells (Fig. 4b–e). A structural analysis of the *uc.339* transcript with the RNAhybrid algorithm^14^ revealed the presence of sequences complementary to three mature miRNAs (namely, *miR-339-3p*, *-663b-3p*, and *-95-5p* from now on referred to as *miR-339*, *-663b*, and *-95*, respectively) in the *uc.339* RNA transcript (Figs. 4f and 5a). We observed downregulation of *miR-339*, *-663b*, and *-95* when A549, H460, H1299, and LoVo cells were stably expressing *uc.339* by lentiviral transduction compared to that in the same cell line transfected with an empty lentiviral construct (Fig. 5b). Conversely, when *uc.339* was silenced with two different siRNAs, upregulation of *miR-339*, -*663b*, and *-95* was detected in all four tested cell lines, by qRT-PCR (Fig. 5c). We previously showed that T-UCRs can be directly targeted by miRNAs^2^. However, we did not observe any significant downregulation of *uc.339* when any of the three miRNAs was transfected in any of the tested cell lines (Fig. 5d). Similarly, the levels of *uc.339* did not vary significantly when the miRNAs were donwregulated by anti-miRNAs (Supplementary Fig. 3). This is in stark contrast with the downregulation we could achieve with our designed siRNAs (Supplementary Fig. 2b), suggesting that the *uc.339*–*miRNA* interaction described in Fig. 5a does not result in miRNA-mediated degradation of *uc.339*. When we immunoprecipitated the *uc.339* transcript in A549, H460, H1299, and LoVo cells overexpressing *uc.339*, we observed an enrichment for *miR-339*, *-663b*, and *-95* in the immunoprecipitate compared to that in the empty vector counterpart (Fig. 6a). We further tested whether the interaction *uc.339::miRNAs* occurs endogenously and whether complementarity is required to “trap” the miRNAs. To do so, we used CRISPR/Cas9 technology to generate two distinct clones of H1299 carrying a genomic bi-allelic deletion (clone 102) and a monoallelic deletion with inversion in the other allele (clone 20) at the predicted interaction site of *uc.339* that is complementary with *miR-339* (Fig. 6b), and used as control a clone with no targeting gRNA (H1299 CTRL clone 18). When we immunoprecipitated the *uc.339* transcript from clones 20 and 102 (compared to clone 18), we almost completely abrogated the co-precipitation of *miR-339*, while *miR-663b* or *miR-95* were not affected (Fig. 6c). We also observed a significantly reduced level of viable H1299 cells from clones 20 and 102 (compared to clone 18) after  72 h in culture (Fig. 6d), and reduced colony-forming ability by clones 20 and 102 (Supplementary Fig. 4), suggesting that the disruption of *miR-339* miRNA-binding element (MBE) in *uc.339* reduces H1299 cell line growth. Since we observed that *uc.339* modulation affects the expression of its host gene *ATP5G2* (Fig. 1d, e), we considered the possibility that the oncogenic effects of *uc.339* are mediated by *ATP5G2*. H1299 CRISPR clones 20 and 102 (with endogenous deletion of *miR-339* MBE on the *uc.339* transcript) grow significantly less and form fewer colonies than H1299 clone 18 (expressing the *uc.339* wild-type counterpart) (Fig. 6d; Supplementary Fig. 4a, b). Furthermore, nude mice (*n* = 5 per group) injected subcutaneously with clone 18 grow tumors much faster than clones 20 and 102 (Supplementary Fig. 4c, d). Of note, the expression of *uc.339* and of *ATP5G2* (both mRNA and protein) was not affected by the CRISPR deletion (Supplementary Fig. 5a, b). Moreover, ex vivo tumors from mice injected subcutaneously with clones 18, 20, and 102 did not show any difference in *uc.339* and *ATP5G2* expression (Supplementary Fig. 5c, d). Overall, these data suggest that the pro-tumoral effects of *uc.339* are not likely mediated by its modulation of the *ATP5G2* host gene, at least in H1299 cells.

### Downstream pathway regulated by *uc.339*

**Fig. 7:**
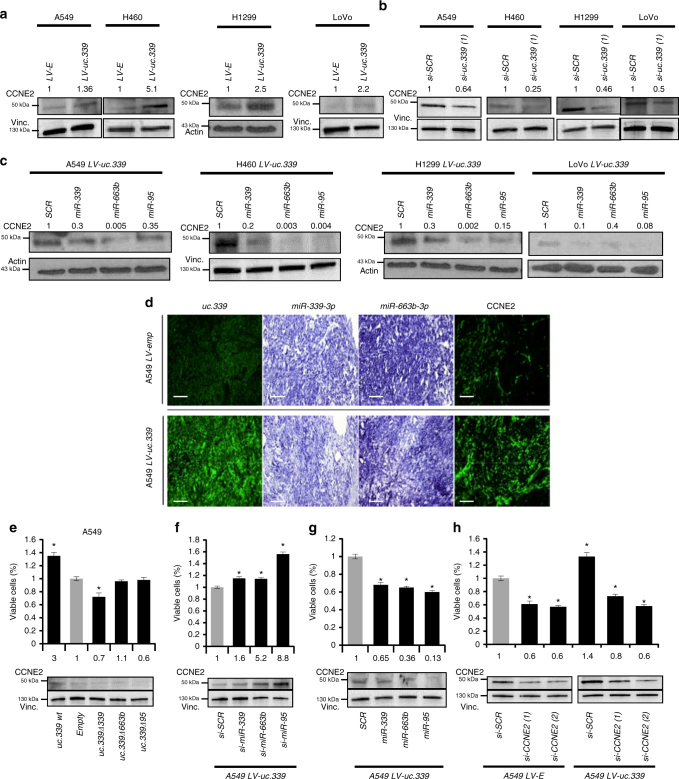
*uc.339* increases the expression of CCNE2 by sequestering its targeting miRNAs. **a** Immunoblotting for CCNE2 and Vinculin in A549, H460, H1299, and LoVo cells infected with LV-*uc.339* or LV-E. **b** Immunoblotting for CCNE2 and Vinculin in A549, H460, H1299, and LoVo cells transfected with si-*uc.339(1)* or si-*SCR* for 72 h. **c** Immunoblotting for CCNE2 and Vinculin in A549, H460, H1299, and LoVo cells infected with LV-*uc.339* and transfected with *miR-339*, *-663b*, and *-95* or a scrambled miRNA (*SCR*) for 48 h from transfection. **d** Representative image of RNA fluorescent in situ hybridization for *uc.339*, *miR-339*, *miR-663b*, and CCNE2 in two nude mice injected subcutaneously with A549 LV-E or A549 LV-*uc.339*. Scale bar = 200 μm. **e** Cell viability assay in A549 cells transfected for 72 h with a plasmid-expressing wild-type *uc.339* (*uc.339 wt*) or expressing *uc.339* in which the miRNA-binding elements of *miR-339*, *-663b*, and *-95* have been deleted (*uc.339Δ339*, *uc.339Δ663b*, and *uc.339Δ95*) or with its empty plasmid counterpart. Data presented as mean ± s.d. of experimental triplicates normalized to empty. *P<0.05. Immunoblotting for CCNE2 and Vinculin of the same experiment are shown in the lower panel. **f** Cell viability assay in A549 LV-*uc.339* transfected with si-*miR-339*, *-663b*, and *-95* or si-*SCR* for 48 h. Data presented as mean ± s.d. of experimental triplicates normalized to si-*SCR*. *P<0.05. Immunoblotting for CCNE2 and Vinculin of the same experiment are shown in the lower panel. **g** Cell viability assay in A549 LV-*uc.339* transfected with *miR-339*, *-663b*, and *-95* or *SCR* for 48 h. Data presented as mean ± s.d. of experimental triplicates normalized to *SCR*. *P<0.05. Immunoblotting for CCNE2 and Vinculin of the same experiment are shown in the lower panel. **h** Cell viability assay in A549 LV-E and A549 LV-*uc.339* transfected with two different siRNAs against CCNE2 (*si-CCNE2* (1), *si-CCNE2* (2)) or si-*SCR* for 72 h. Data presented as mean ± s.d. of experimental triplicates normalized to *LV-E*. *P<0.05. Immunoblotting for CCNE2 and Vinculin of the same experiment are shown in the lower panel. The numbers above the bands represent the quantification of the band intensity normalized to Vinculin and to controls

Next, we performed an Affymetrix mRNA array analysis of A549 and LoVo cells infected with a lentiviral empty vector (control) or with a lentiviral vector expressing *uc.339*. We identified a list of 122 genes upregulated in *uc.339* overexpressing cells and common to both cell lines, and the top five genes upregulated were *EXOC5*, *CCNE2*, *RAB8B*, *EFEMP1*, and *MYO1B* (Supplementary Fig. 6). We decided to focus on Cyclin E2 (CCNE2) as it has been shown to have oncogenic role in NSCLC^15–19^, and is also a predicted target for *miR-339*, *-663b*, and *-95* ^20–22^. We validated *CCNE2* as a direct target of these three miRNAs by performing a luciferase reporter assay. A549, H460, H1299, and LoVo cells were co-transfected with a plasmid carrying the *CCNE2* 3′-UTR region cloned downstream of the luciferase reporter gene, and *miR-339*, *-663b*, *-95*, or a scrambled miRNA as a control. Reduced luciferase activity was observed with all three miRNAs compared to that in the scrambled transfected cells, and this effect was abolished when each of the three in silico predicted miRNA-binding sites on *CCNE2* messenger RNA was deleted (Supplementary Fig. 7a). We also showed that CCNE2 protein levels were reduced in the four cell lines transfected with *miR-339*, *-663b*, or *-95* compared with scrambled miRNA cells (Supplementary Fig. 7b). Next, we investigated whether *uc.339* indirectly affected CCNE2 expression levels by inducing downregulation of *miR-339*, *-663b*, and *-95*. Our digital PCR data show that *uc.339* is expressed much less than *miR-339*, *-663b*, and *-95* in cancer cells (Supplementary Fig. 1a), raising the concern on whether *uc.339* can affect the expression of *CCNE2* by modulating its targeting miRNAs. We reasoned that a stable *uc.339* transcript might compensate for the relatively low endogenous expression of *uc.339* compared to that in the miRNAs. Therefore, we treated H1299 cells with actinomycin D (or PBS, as control) for up to 10 h and the *uc.339* RNA expression was detected at different time points by qRT-PCR, and compared to that of *MYC* mRNA (a highly regulated transcript with a short half-life^23–26^) and of *p16ink4* mRNA (a transcript considered highly stable^27, 28^). We observed that the *uc.339* transcript had an intermediate stability compared to *MYC* and *p16ink4* (Supplementary Fig. 8). Next, we stably overexpressed *uc.339* through lentiviral infection in A549, H460, H1299, and LoVo cells and observed upregulation of CCNE2 protein in all four cell lines (Fig. 7a). Then, we silenced *uc.339* expression in the same cells and observed a downregulation of CCNE2 protein expression compared to that in the si-*SCR* control (Fig. 7b; Supplementary Fig. 9a). Also, we observed downregulation of CCNE2 protein levels in H1299 CRISPR clones 20 and 102 (compared to that in the control clone 18), suggesting that CCNE2 regulation by the *uc.339* transcript is at least in part affected by the presence of the MBE for *miR-339* in the *uc.339* transcript (Supplementary Fig. 9b). Next, we showed that in stably *uc.339* overexpressing cells, the upregulation of CCNE2 could be reversed by co-transfection with *miR-339*, *-663b*, or *-95* (Fig. 7c). We also checked the expression of *uc.339*, *miR-339*, *663b*, and *CCNE2* mRNA in ex vivo xenografts from mice injected with A549 LV-E or A549 LV-*uc.339* by fluorescent in situ hybridization. We observed that tumors with higher expression of *uc.339* also had lower expression of *miR-339* and *miR-663b*, but higher levels of *CCNE2* mRNA (Fig. 7d). Next, we transfected A549 cells with a plasmid-expressing wild-type *uc.339* (wt *uc.339*) or mutants of *uc.339* in which the MBEs of *miR-339*, *-663b*, and *-95* had been deleted (*uc.339* *Δ* *339*, *uc.339* *Δ* *663b*, *uc.339* *Δ* *95*) or with its empty plasmid counterpart. Deletion of the MBE in the *uc.339* transcript prevented *CCNE2* upregulation and significantly reduced cell viability (Fig. 7e). Conversely, individual silencing of each of the three miRNAs further increased CCNE2 protein levels and cell viability in A549 LV-*uc.339* compared to that in the siRNA anti-Scrambled transfected cells (Fig. 7f). Downregulation of *CCNE2* and reduced cell viability were observed in A549 LV-*uc.339* transfected with *miR-339*, *-663b*, or *-95* mimics compared to that in their scrambled counterparts (Fig. 7g). Finally, silencing of *CCNE2* with two different siRNAs reduced cell viability of both A549 LV-E and A549 LV-*uc.339* cells compared to that in siRNA anti-scrambled transfected cells (Fig. 7h). Overall, these experiments indicate that *uc.339* affects the expression of *CCNE2* and cancer cell viability by modulating the expression of *miR-339*, *-663b*, and *-95* through a mechanism that “traps” the miRNA in the *uc.339* RNA, with no degradation of the *uc.339* RNA.

### Lack of miRNA effects on *uc.339* expression

**Fig. 8:**
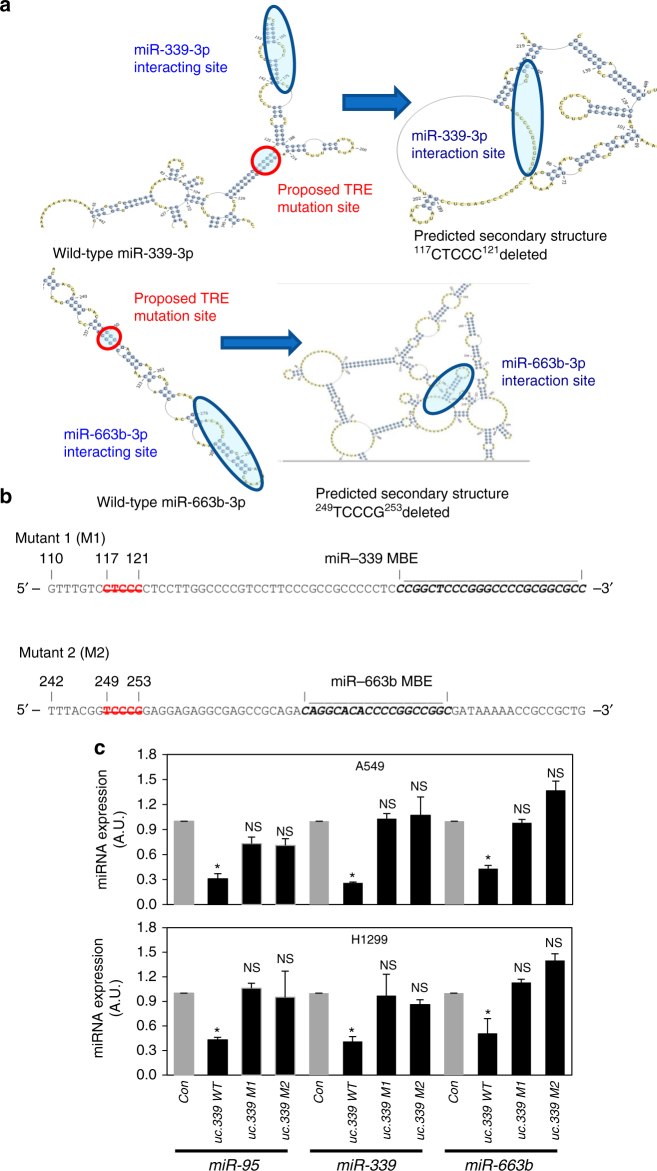
Identification of trapping-related elements (TRE) in the *uc.339* transcript. **a** Proposed local structural effect of deletions in TRE in the *uc.339* RNA transcript by using RNAfold from the ViennaRNA Package. **b** Mutants of the TRE sequences in the *uc.339* transcript. The TREs are indicated in red, and their position related to the beginning of the *uc.339* transcript (as in Supplementary Fig. 1c) is indicated by the numbers above. In bold, the predicted miRNA-binding elements (MBEs) for *miR-339* or *miR-663b*. The figure shows which specific bases were deleted to generate mutant 1 (M1) and mutant 2 (M2) for the experiments in Fig. 8c. **c** qRT-PCR for *miR-95*, *-339*, and *-663b* in A549 and H1299 cells untreated (Con) or transfected with a plasmid-expressing wild-type *uc.339* (uc.339 WT) or TRE M1-mutated *uc.339* (uc.339 M1) or TRE M2-mutated *uc.339* (uc.339 M2). The expression of miRNAs is presented as normalized to the Con group. Data are shown as mean ± s.d. of experiments conducted in triplicate. *P < 0.05. NS not significant

Intriguingly, while modulation of *uc.339* affects miRNA expression (Fig. 5b, c), no variations in the levels of *uc.339* were observed when the three binding miRNAs were overexpressed or downregulated (Fig. 5d; Supplementary Fig. 3). In a classical miRNA sponge interaction, we would also expect downregulation of *uc.339* in response to miRNA upregulation, due to RISC activity. Since we did not observe such downregulation, we reasoned that a different mechanism could be at work. Specifically, we performed a bioinformatics RNA secondary analysis of the *uc.339* structure using the ViennaRNA Package 2, and identified two elements in close proximity with, but located outside of the *miR-339* and the *miR-663b* binding sites, whose deletion completely changed the predicted secondary RNA structure of the *uc.339*, possibly preventing the *uc.339::miRNA* interactions (Fig. 8a). We called these elements trapping-related elements (TRE), and generated two mutants of *uc.339* called M1 and M2, lacking the TRE in the proximity of *miR-339* and *miR-663b* binding sites, respectively (Fig. 8a, b). When A549 and H1299 cells were transfected with M1 or M2, no downregulation of *miR-339*, *-663b*, or *-95* was observed (Fig. 8c), suggesting that TREs affect the ability of all three miRNAs (including those whose MBE is distant from the specific TRE) to bind to *uc.339*. As the interaction of *uc.339* with miRNAs does not result in the degradation of the ultraconserved transcript, we call this mechanism “entrapping”.

### *uc.339* is transcriptionally silenced by TP53

**Fig. 9:**
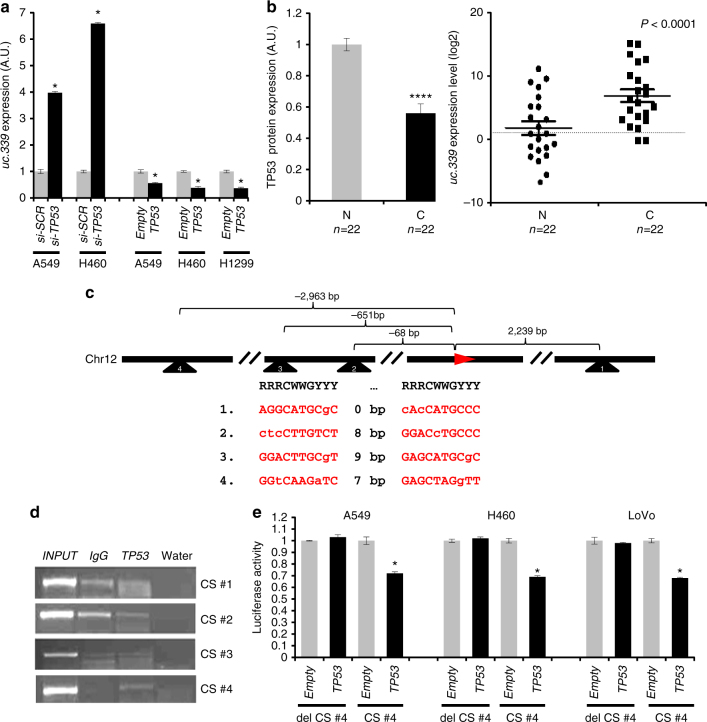
*uc.339* expression is directly silenced by TP53. **a** qRT-PCR for *uc.339* in A549 and H460 cells transfected with an anti-*TP53* siRNA (si-*TP53*) (or its anti-scrambled control (si-*SCR*)), and in A549, H460, and H1299 cells transfected with a plasmid-expressing *TP53* (or its empty plasmid counterpart) for 72 h. The levels of *uc.339* have been normalized to *RNU44* RNA and the results are presented as normalized to si-*SCR* or empty, respectively. Data are presented as mean ± s.d. of experiments conducted in triplicate. *P < 0.05. **b** Quantification of TP53 protein (left panel) and of *uc.339* expression (right panel) in 22 paired primary NSCLC cancerous tissues (C) and the adjacent non-tumor lung (N). TP53 expression has been quantified with enhanced chemiluminescence (Femto) Kit and normalized to Vinculin, whereas *uc.339* expression has been determined by qRT-PCR, normalized to *RNU44* and log2 transformed. Data are shown as mean ± s.d., and referred to N mean value. The linear fold-change between C and N was 65.6. Paired *t*-test *P*-value <0.0001 (****). **c** Map showing the location of four identified TP53 consensus sequences (CS #1–4) relative to the transcription starting nucleotide of the *uc.339* gene on chromosome 12. **d** Chromatin Immunoprecipitation showing the binding of TP53 to the four identified *TP53* CS. **e** Luciferase reporter assay in A549, H460, and LoVo cells transfected with a *TP53*-expressing plasmid (or its empty plasmid counterpart) and a reporter plasmid containing the sequence of CS #4, or a plasmid in which CS #4 has been deleted (del CS #4). Luciferase activity has been normalized to Renilla (RLU), and the results are presented as normalized to empty. Data are shown as mean ± s.d. of experiments conducted in triplicate. *P < 0.05

The observed upregulation and oncogenic function of *uc.339* in NSCLC patients prompted us to investigate how the expression of *uc.339* is regulated. By analyzing the endogenous expression of *uc.339* in the three NSCLC cell lines and one CRC line (Supplementary Fig. 1a), we noticed that while *uc.339* is expressed at the lowest levels in wild-type (wt) TP53-expressing cells (A549), the expression was the highest in TP53-null H1299 cells (Supplementary Fig. 1a; Supplementary Fig. 10). We transfected A549 and H460 cells with a siRNA anti-*TP53* (or si-*SCR* control) and A549, H460, and H1299 cells with a *TP53*-expressing plasmid (or empty plasmid control) (Supplementary Fig. 11). We observed a significantly increased expression of *uc.339* in cells treated with siRNA anti-*TP53* and a reduced expression of *uc.339* in cells treated with the *TP53*-expressing plasmid (Fig. 9a). Also in primary NSCLC patients selected after sequencing for their *TP53* mutation status and included only if carrying a wt *TP53* status (*n* = 22), we determined the expression of TP53 protein and *uc.339* RNA levels both in cancerous tissues and in adjacent non-tumor lung tissue. We observed that non-tumor lung tissues had higher expression of TP53 and lower expression of *uc.339* RNA compared to that in the matched cancerous tissues, whereas cancerous tissues had lower expression of TP53 and higher levels of *uc.339* (Fig. 9b). These data suggest a possible regulatory function of TP53 on *uc.339* expression. Next, we searched for TP53 consensus sequences (CS) in the *uc.339* locus. We identified at least three TP53 CS located upstream of the *uc.339* locus on chromosome 12q13.13 and one TP53 CS located 2239 bp downstream of the beginning of *uc.339* transcription start site (Fig. 9c). To determine whether TP53 binds to the predicted CS, we performed chromatin immunoprecipitation in H1299 cells transfected with a plasmid-expressing TP53 (or its empty counterpart as a control) and observed an enrichment of CS #4 (located 2963 bp upstream of the beginning of *uc.339* transcription) only in the immunoprecipitate of *TP53*-transfected cells, suggesting that TP53 binds to this site (Fig. 9d). To investigate whether TP53 binding to CS #4 leads to transactivation or silencing, we cloned CS #4 in a reporter vector upstream of the luciferase gene. We observed a reduced luciferase activity when a plasmid overexpressing *TP53* (compared to that in the corresponding empty plasmid counterpart) was co-transfected with the CS #4 containing luciferase plasmid in A549, H460, and LoVo cells. This reduction was completely abolished when CS #4 was deleted, even in the presence of co-transfection with the *TP53* overexpressing plasmid (Fig. 9e). Overall, these data indicate that TP53 binds to a CS located upstream of the *uc.339* gene and directly inhibits the expression of *uc.339* RNA.

### TP53 reverts *uc.339*-mediated regulation of *CCNE2* expression

Our data indicate that *uc.339* is directly silenced by TP53 (Fig. 9) and that *uc.339* sequesters *miR-339*, *-663b*, and *-95* (Figs. 5 and 6) leading to upregulation of CCNE2 (Fig. 7) and increased tumor growth and migration both in vitro and in vivo (Figs. 2 and 3). The tumor-suppressor TP53 is mutated in about 50% of NSCLC^12, 13^ and in several other human cancers^11^; therefore, we investigated whether restoration of functional *TP53* expression could revert the oncogenic effects of *uc.339*.

**Fig. 10:**
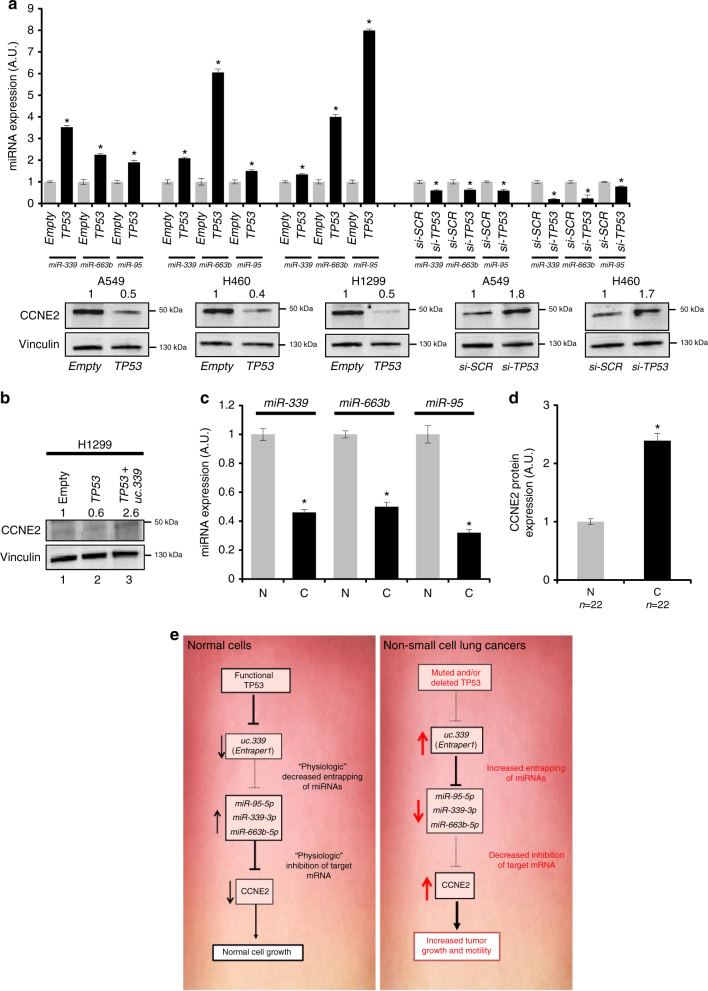
TP53 reverts *uc.339* effects on miRNA and CCNE2 expression. **a** qRT-PCR for *miR-339*, *-663b*, and *-95* in A549, H460, and H1299 cells transfected with a plasmid-expressing *TP53* (or its empty plasmid counterpart) and in A549 and H460 cells transfected with an anti-*TP53* siRNA (si-*TP53*) [or its anti-scrambled control (si-*SCR*)] after 72 h from the transfection. The expression of miRNAs was normalized to *RNU44* RNA and the results are presented as normalized to empty. Data are shown as mean ± s.d. of experiments conducted in triplicate. *P < 0.05. The immunoblotting for CCNE2 and Vinculin is represented for each cell line and treatment group under the qRT-PCR data. **b** Immunoblotting for CCNE2 and Vinculin in H1299 transfected with an empty plasmid (lane 1), the same plasmid-expressing *TP53* (lane 2) or co-transfected with a plasmid-expressing *TP53* and a plasmid-expressing *uc.339* (lane 3) after 72 h from the transfection. The numbers above each lane represent a quantification of the band intensity, normalized to the corresponding Vinculin band. **c** qRT-PCR for *miR-339*, *-663b*, and *-95* in the same 22 primary NSCLC samples described in Fig. 9b. The expression of miRNAs has been normalized to *RNU44* RNA and the results are presented as normalized to N. Data are shown as mean ± s.d. of experiments conducted in triplicate per each patient. *P < 0.05. **d** Quantification of CCNE2 protein in the same 22 primary NSCLC samples described in Fig. 9b. CCNE2 expression has been quantified by enhanced chemiluminescence (Femto) kit, normalized to Vinculin, and presented as normalized to N. Data are shown as mean ± s.d. *P < 0.05. **e** The TP53-*uc.339*-*miRNA*-CCNE2 network. TP53 directly inhibits the expression of *uc.339*. *uc.339* functions as an “entrapper” for CCNE2 targeting *miR-339*, *-663b*, and *-95* leading to upregulation of CCNE2 and increased tumor growth and migration

First, we transfected A549, H460, and the *TP53*-null H1299 cells with a plasmid-expressing wt *TP53* or with its empty plasmid counterpart, and determined the effects on *miR-339*, *-663b*, and *-95* and *CCNE2* expression, by qRT-PCR and immunoblotting, respectively. Also, we transfected A549 and H460 cells with siRNA anti-TP53. Consistent with our hypothesis, overexpression of TP53 induced upregulation of the three miRNAs and downregulation of their target gene *CCNE2*, whereas silencing of *TP53* induced downregulation of the three miRNAs and upregulation of *CCNE2* (Fig. 10a). Moreover, when H1299 transfected with the *TP53* overexpressing plasmid were also transfected with a plasmid overexpressing *uc.339*, the expression of CCNE2 was rescued (Fig. 10b), suggesting that TP53 downregulation of CCNE2 is, at least in part, mediated by TP53 silencing of *uc.339*. These data also indicate that re-expression of functional TP53 can revert the oncogenic effects of *uc.339*, mitigating its miRNA-entrapping effect and the resulting *CCNE2* induction in NSCLC. Finally, we validated the TP53-*uc.339*-*miR-339*/*-663b/-95*-CCNE2 axis in 22 primary NSCLC samples, where we observed that when TP53 was downregulated and *uc.339* was upregulated (Fig. 9b), *miR-339*, *-663b*, and *-95* were downregulated (Fig. 10c) and CCNE2 protein expression was increased (Fig. 10d). Overall, these data indicate that *uc.339* exerts its oncogenic function, at least in part, by entrapping complementary mature *miR-339*, *-663b*, and *-95* and releasing oncogenic CCNE2 from its miRNA-controlled regulation in NSCLC (Fig. 10e).

## Discussion

This work identifies *uc.339* as upregulated in primary NSCLC compared to that in the adjacent non-tumor lung. We also show that *uc.339* increases lung cancer cell growth both in vitro and in in vivo xenograft murine models, and increases cancer cell migration in vitro, therefore, acting as a bona fide oncogene. We also previously described the upregulation of *uc.339* in CRC primary samples^2^, and another report confirmed an oncogenic role for *uc.339* in HCC^9^, although limited to in vitro experiments. The mechanism of action and regulation of *uc.339* and T-UCRs in general are currently unknown. In particular, it is unclear how T-UCR dysregulation occurs in cancerous tissues, and how they contribute to cancer cell increased growth and invasiveness. Although many T-UCRs act as long-range enhancers during mouse development^29^, this function has not been confirmed for most human T-UCRs, and it has been shown that similar proportions of functional enhancers can be found in less conserved sequences^30^. In this study, we show that *uc.339* harbors three sequences complementary to the mature sequence of *miR-339*, *-663b*, and *-95*, and that *uc.339* appears to function as a “decoy”, sequestering these three miRNAs. As a result, the mRNA of *CCNE2* (which we also show is a direct target of *miR-339*, *-663b*, and *-95*) is released from the post-transcriptional regulation exerted by the three miRNAs, and the expression of *CCNE2* is increased, leading to increased cell proliferation and motility. We cannot exclude that the *uc.339* transcript also binds to other miRNAs also targeting *CCNE2* or other genes, and a comprehensive bioinformatics and functional study of all the miRNAs interacting with *uc.339* is warranted. Of the three interacting miRNAs, *miR-339-3p* is very conserved among species, *miR-95-5p* is conserved in humans, pig, dog, cow, and horse, but not in mouse; so it evolved after the mouse appearance, but long before humans, whereas *miR-663b-3p* is primate specific. The fact that an ultraconserved element such as *uc.339* interacts with highly conserved and highly primate-specific miRNAs does not detract to the importance of the observed mechanism for human carcinogenesis. Cyclin E2 belongs to the highly conserved cyclin family, interacts as a regulatory subunit of CDK2, and has a role in cell-cycle G1/S transition^31–33^. CCNE2 is frequently upregulated in human pulmonary dysplasia and malignancy and is correlated with poor prognosis in lung cancer patients^15–19^. In several cancer types (including NSCLC and breast cancer), CCNE2 upregulation occurs as an early event^15, 34^, and overexpression of this cyclin acts as an inducer of genomic instability and polyploidy, differently from cyclin E1, D, or A overexpression^35, 36^. Although we did not determine the impact of *uc.339*-induced upregulation of CCNE2 on the genomic instability of the tested cell lines, this area warrants further investigation. It has also been shown that the CCNE2–CDK2 axis inhibits SIRT2 through a Ser-331 phosphorylation, leading to increased cell migration^37^. We observed overexpression of CCNE2 and increased motility in cancer cells overexpressing *uc.339*. Whether this phenotype is determined by the effects on SIRT2 phosphorylation remains to be elucidated.

It has been shown that CCNE2 levels are upregulated in NSCLC tissues, and that *miR-30d-5p*, by targeting *CCNE2*, inhibits lung cancer cell proliferation and motility^38^. Our study shows that *CCNE2* is directly targeted by *miR-339*, *-663b*, and *-95*. *MiR-95* is overexpressed in pancreatic, prostate, breast, and colorectal cancer, where it promotes cell proliferation by targeting Nexin-1^39^. *MiR-95* promotes resistance to radiotherapy in prostate and breast cancer cells by targeting Sphingolipid Phosphatase SGPP1^40^. *MiR-339-5p* is downregulated in breast cancer cell lines, where it inhibits cell migration and invasiveness, and represents a marker of bad prognosis when downregulated^41^. Moreover, *miR-339-5p* promotes resistance of glioma cells to cytotoxic T-lymphocytes, by targeting ICAM-1^42^. The expression of *miR-339-3p* is dysregulated in the more aggressive NSCLC, identified in later years of screening by computed tomography^43^, suggesting a role for this miRNA in lung carcinogenesis.

A dual nature both as an oncogene and as a tumor-suppressor gene has also been reported for *miR-663*. Although *miR-663* can be induced by the anti-inflammatory drug resveratrol, and acts as a tumor-suppressor miRNA in human THP1 monocyte cells as well as in human blood monocytes by targeting AP-1 and preventing LPS-mediated upregulation of the oncogenic *miR-155* ^44^, in nasopharyngeal carcinoma *miR-663* promotes cancer proliferation by targeting p21 (WAF1/CIP1)^45^, and in prostate cancer overexpression of *miR-663* is associated with increased castration-resistance^46^. Therefore, the existing literature supports that different miRNAs can have distinct effects in different cancer types.

In this study, we observed that *uc.339* can bind to all these three miRNAs in complementary sites positioned along the *uc.339* sequence, also suggesting the possibility that this T-UCR functions as a competing endogenous RNA (ceRNA) for the three miRNAs, regulating their availability to target *CCNE2*. The ceRNA hypothesis would imply a reciprocal modulation of the ceRNA transcript and of the interacting miRNAs^47–49^. However, our study shows that while upregulation of *uc.339* induces downregulation of *miR-339*, *-663b*, *and -95*, increased (or reduced) levels of the three miRNAs do not affect the expression of *uc.339*. Therefore, the type of interaction between *uc.339* and *miR-339*, *-663b*, *and -95* cannot be considered a ceRNA mechanism *sensu strictu*. We call the observed interaction “entrapping”. Specifically, in this work we described this type of interaction between a long non-coding RNA (*uc.339*) and miRNAs, in which specific sequences in the *uc.339* transcript (that we named TRE) induce conformational changes in the secondary structure of *uc.339*, affecting the ability of miRNAs to interact with the long non-coding RNA at the miRNA-binding sites. Our data support the hypothesis that *uc.339* could bind and “hide” miRNAs attached to their MBEs. However, if TREs are mutated, miRNAs cannot bind any longer (or cannot be retained) at the location of their MBEs. Therefore, the existence of TREs could explain why the modulation of *uc.339* reduces the levels of miRNAs, whereas the upregulation of miRNAs does not reduce *uc.339* levels. *Uc.339* is the first example of an ‘Entrapper” long ncRNAs, and others will be certainly discovered; for example, it was published that the long ncRNA SNH6-003 interacts with miR-26a/b and the levels of this ncRNA did not change when the Huh7 cells are treated with either miR-26a or b mimics or inhibitors^50^. Future research will explore this type of interaction. Whether a long ncRNA (ceRNA or entrapper) can affect the expression of a miRNA target gene by competing for same miRNA-binding sites is still a very controversial question^51, 52^. One of the unsolved issues is whether changes in the expression of an individual long ncRNA, usually expressed at very-low endogenous levels in cells, would be sufficient to affect the expression of a more abundant target miRNA transcript. We found that the endogenous *uc.339* transcript is quite stable in our cell lines and that one *uc.339* transcript is able to entrap not one but at least three different miRNAs sharing a common target gene, providing a possible further clue on how long ncRNAs may affect miRNA target gene expression despite their generally low expression. Our data show an inverse expression of *uc.339* and its “host” gene *ATP5G2*. These findings suggest that *uc.339* could also act as an antisense transcript for *ATP5G2* mRNA. However, our experiments with the CRISPR clones of *uc.339* exclude that the oncogenic effects of *uc.339* are mediated by its modulation of *ATP5G2* at least in H1299 cells. Moreover, it cannot be excluded that *uc.339* also functions as an enhancer RNA (eRNA) on other transcripts, as recently shown for other non-coding RNAs^53^. The entrapping mechanism is likely not the only mechanism of action, and further study will clarify these aspects of the *uc.339* biology.

Finally, we observed that the tumor-suppressor TP53 directly silences *uc.339*. We also observed an inverse correlation between TP53 and *uc.339* expression in primary NSCLC samples, confirming a negative regulation by TP53. Since mutations of the *TP53* gene occur in about 50% of NSCLC and are very frequent in several cancer types, the identified mechanism could represent a significant event in lung carcinogenesis, with possible implications for other cancer types. The biology of *TP53* is complex and further studies will be needed to investigate whether *TP53* mutants affect *uc.339* expression and its downstream miRNA-CCNE2 pathway. However, this study provides evidence of a *TP53* transcriptional regulation of an ultraconserved element.

In conclusion, this study suggests that loss of TP53 activates *uc.339* as an oncogene in NSCLC by entrapping *miR-339*, *-663b*, and *-95*, and preventing their targeting of oncogenic *CCNE2*, leading to increased tumor growth and migration. These findings identify *uc.339* as an important molecular target for NSCLC and other types of cancer in which TP53 expression is impaired.

## Methods

### Patient samples and cell lines

Paired frozen tumor and adjacent non-tumor lung from 30 NSCLC patients were obtained at the Istituto Scientifico Romagnolo per lo Studio e la Cura dei Tumori IRST, S.r.l, IRCCS, in Meldola (FC), Italy. Written informed consent was obtained from all patients before sample analyses.

All cell lines were purchased from American Type Culture Collection and were maintained as a monolayer at 37 °C. A549 (lung adenocarcinoma) and LoVo (colorectal adenocarcinoma) cells were grown in F12K medium (ATCC), supplemented with 10% FBS, whereas H1299 (lung adenocarcinoma) and H460 (large cell lung cancer) cells were cultured in RPMI 1640 (ATCC) with 10% FBS. 293TN cells were used for the packaging of the *uc.339*-expressing lentivirus (or its empty lentiviral counterpart) and were grown in DMEM medium (ATCC), supplemented with 10% FBS. Each cell line was tested for the presence of mycoplasma every 2 months (MycoAlert™ Mycoplasma Detection Kit—Lonza).

### Chromatin immunoprecipitation

The TP53 binding sites upstream of the *uc.339* were identified by combining a published global map of the TP53 transcription factor binding sites^54^ with the OMGProm algorithm^55^. Chromatin immunoprecipitation (ChIP) was performed using the EZ-ChIP kit (Millipore) and 5 μg of anti-TP53 antibody (Santa Cruz Biotechnology) on CMV-TP53/Empty transfected H1299. As a negative control, we immunoprecipitated one sample with 5 μg of pre-immune serum (Santa Cruz Biotechnology). After final elution and purification of DNA, the samples were PCR amplified using specific primers. The input sample was diluted to 0.1 ng l^−1^ and was used as a positive control for PCR. PCR was performed using the AmpliTaq Gold^®^ PCR Master Mix (Applied Biosystems).

### Isolation of *uc.339*–*miRNA* complexes

The immunoprecipitation of the *uc.339* transcript was performed as follows: total RNA was extracted from LV-*uc.339* or LV-E infected A549, H460, H1299, and LoVo cells with complete RIPA buffer (Santa Cruz Biotechnology) following the manufacturer’s instructions. Next, we incubated the extracted RNA with 120 pmoles of a 3′-biotinylated RNA probe (Supplementary Table 1) that was complementary to the *uc.339* RNA transcript and did not overlap with the transcript sequence of the *ATP5G2* host gene, overnight at 4 °C. Co-precipitation of the bound *uc.339*-*miRNA* complexes was performed by using streptavidin-conjugated magnetic beads (Miltenyi Biotec), according to the manufacturer’s instructions^56^.

### TCGA data set analysis

The cases from TCGA analysis consisted of 210 Lung squamous cell carcinoma (LUSC) samples with overall survival information and *uc.339* expression computed. Clinical data were retrieved from cbio portal (http://www.cbioportal.org/). We downloaded RNA-seq BAM files from UCSC Cancer Genomics Hub (CGHub, https://cghub.ucsc.edu/). TCGA BAM files were generated based on Mapsplice2 algorithm^57^ for alignment against the hg19 reference genome using default parameters. We quantified the expression of *uc.339* as RPKM (reads per kilobase per million mapped reads)^58^. The data can be retrieved from TANRIC http://ibl.mdanderson.org/tanric/_design/basic/index.html. We downloaded patient clinical information for the TCGA patients with LUSC from cbioPortal (http://www.cbioportal.org/).

### Study of the *TP53* gene mutation status in primary NSCLC samples

Sequencing of *TP53* was performed on exons 5, 6, 7, and 8 of the *TP53* gene in all NSCLC patients included in this study.

Tumor DNA from each patient was extracted with the QIAamp DNA Mini Kit (Qiagen) and purified with the QIAamp DNA Micro Kit (Qiagen). The fragment between exons 2 and 11 of the *TP53* gene was amplified with the primers F2 and R11 (listed in Supplementary Table 1), at an annealing temperature of 68 °C with the LA Taq^®^ DNA Polymerase (Takara). The PCR fragment was purified with MinElute PCR Purification kit (Qiagen), according to the manufacturer’s instructions. Exons 5–8 were sequenced with the BigDye^®^ Terminator v3.1 Cycle Sequencing kit (Life Technologies) at 56 °C annealing temperature, using primers 5F, 5R, 6F, 6R, 7F, 7R, 8F, and 8R listed in Supplementary Table 1.

Subsequently, the sequences were purified with the DyeEx 2.0 Spin kit (Qiagen) and loaded in a 3130 Genetic Analyzer (Applied Biosystems) with Montage Injection Solution (Millipore).

### qRT-PCR and QuantStudio 3D digital PCR

RNA was extracted using TRIzol^®^ reagent (Invitrogen), according to the manufacturer’s instructions. RNA concentration and quality were assessed with a NanoDrop^®^ ND-1000 Spectrophotometer (Thermo Scientific). RNA was treated with the TURBO DNA-free™ kit (Ambion).

Retrotranscription of *uc.339* was performed using the RT *uc.339* reverse primer (Supplementary Table 1) designed so to make sure that it does not overlap with the sequence of the transcript of the *ATP5G2* host gene (Fig. 1c) and by using the TaqMan^®^ MicroRNA Reverse Transcription kit (Applied Biosystems), according to the manufacturer’s instructions. The *uc.339* cDNA was pre-amplificated with TaqMan^®^ PreAmp Master Mix (Applied Biosystems) and next qRT-PCR was assessed in triplicate with TaqMan^®^ Universal PCR Master Mix (Applied Biosystems) according to the manufacturer’s instructions. *RNU44* was used as a normalizer for qRT-PCR.

Retrotranscription of *ATP5G2*, *TP53*, and *CCNE2* was obtained using the TaqMan^®^ Reverse Transcription Reagents kit (Applied Biosystems), according to the manufacturer’s instructions and qRT-PCRs was tested in triplicate with TaqMan^®^ Universal PCR Master Mix (Applied Biosystems) according to the manufacturer’s instructions. *HPRT*1 gene was used as a housekeeping gene.

Retrotranscription of *miR-339-3p*, *-663b-3p*, and *-95-5p* was carried out by using the TaqMan^®^ MicroRNA Reverse Transcription kit (Applied Biosystems), according to the manufacturer’s instructions and qRT-PCRs were made in triplicate with the TaqMan^®^ Universal PCR Master Mix, no AmpErase^®^ UNG reagents (Applied Biosystems), according to the manufacturer’s instructions. RNU44 was used as a normalizer for qRT-PCR.

All quantitative retrotranscriptions and qRT-PCR reactions were performed in an Applied Biosystems^®^ 7500 Real-Time PCR System (Applied Biosystems), according to the operator’s manual.

Digital PCR was performed using with QuantStudio 3D digital PCR system (Thermo Fisher Scientific). Briefly, cDNA for *uc.339*, *miR-339-3p*, *-663b-3p*, *-95-5p*, *and CCNE2* was obtained through retrotranscription as described above in this section. cDNA was amplified in a 15.5 µl reaction volume containing 0.725 µl of TaqMan PCR assay, 7.25 µl of Quantstudio 3D Master Mix 2, and water. Each digital chip was prepared by loading 14.5 µl of reaction mix. Thermal cycling conditions were: 96 °C for 10 min, then 45 cycles at 60 °C for 2 min and 98 °C for 30 s, and a final step at 60 °C for 2 min. The PCR was analyzed by QuantStudio 3D software (Thermo Fisher Scientific). Poisson distribution was used to estimate the average number of copies per reaction microliters.

### Rapid amplification of cDNA ends (RACE)

To identify the 5′- and 3′-end of the *uc.339* transcript, H1299 and LoVo cell total RNAs were treated with DNase I (RNase-free) (Invitrogen) and the SMARTer RACE cDNA Amplification Kit (Clontech) was used, according to the manufacturer’s instructions. The cDNA ends were amplified with the Platinum Taq DNA Polymerase High Fidelity (Invitrogen) and gene-specific primers (listed in Supplementary Table 1) were used. The primer for the 5′-end was designed to not overlap with the transcript of the *ATP5G2* host gene, to make sure that only the *uc.339* transcript was amplified. Furthermore, we performed a nested PCR with the nested universal primer provided with the kit and the nested gene-specific primers listed in Supplementary Table 1. Placental RNA and Transferrin receptor-specific primers provided with the kit were used as reaction controls. The PCR fragments were then run on a 1.5% agarose gel, and DNA was extracted with the QIAquick Gel Extraction Kit (Qiagen), according to the manufacturer’s instructions. The RACE products were then cloned into a TOPO^®^ TA pCR^®^2.1 cloning vector (Invitrogen), according to the manufacturer’s instructions, and the inserts were sequenced by using the T7 and T3 primers and blasted with the UCSC Genome Browser website (http://genome.ucsc.edu/cgi-bin/hgBlat?command=start).

### Reagents and transfection conditions

The TP53-expressing vector TrueClone pCMV6-XL5 (pCMV-*TP53*) and its empty control vector were purchased from OriGene. The *uc.339* RACE sequence was cloned into the pCDH-CMV-MCS-EF1-copGFP expression vector (pCDH-*uc.339*) (System Biosciences), by using the *Eco*RI and *Not*I restriction sites and the primers indicated in Supplementary Table 1. This plasmid was also used to generate *uc.339*-expressing lentiviral particles (LV-*uc.339*) or their empty lentiviral vector controls (LV-E), according to the Lentiviral Expression Technology (System Biosciences) and following the manufacturer’s instruction. The *uc.339* plasmid and empty plasmid were transfected with the pPACK™ Lentivector Packaging System (System Biosciences) in the 293TN packaging cell line. The obtained viruses were concentrated using the PEG-it™ Virus Precipitation Solution (System Biosciences), and lentiviral particle titration was performed by using the Global UltraRapid Lentiviral Titer Kit™ (System Biosciences). A549, H460, H1299, and LoVo cells were infected at an MOI (multiplicity of infection) of 10 and were selected for green fluorescence by cytofluorimetry with a BD FACSCalibur platform (BD Biosciences). After cytofluorimetric cell selection, the infection efficiency resulted >90% by fluorescent microscopy and the expression of the *uc.339* transcript was further validated by qRT-PCR. As a control vector, we performed a deletion of the miRNA consensus sequences in the *uc.339* plasmid with the mutagenesis primers listed in Supplementary Table 1 and by using the QuikChange II XL Site-Directed Mutagenesis kit (Stratagene), following the manufacturer’s instructions.

The pCMV-*TP53* and pCDH-*uc.339* plasmids were transfected in cell lines with Lipofectamine^®^ LTX (Invitrogen), at a final concentration of 1 μg ml^−1^ and according to the manufacturer’s instructions. The precursors of *miR-95-5p*, *-339-3p*, and *-663b-3p* and *si-miR-339-3p*, *-663b-3p*, *and -95-5p* (as well as the scrambled miRNA negative control #1) were purchased from Ambion, and were transfected in cell lines at a final concentration of 100 nM, by using Lipofectamine^®^ 2000 (Invitrogen), according to the manufacturer’s instructions.

Two siRNAs against two different regions of the *uc.339* transcript were designed as follow: *si-uc.339(1)* 5′-GGGAAUCAAUCAACAGGUATT-3′ and *si-uc.339(2)* 5′-CUCCAGUUUUAGUUGUUGATT-3′. These two siRNAs do not overlap with the sequence of the transcript of the *ATP5G2* host gene, to avoid cross-silencing of the host gene which might interfere with data interpretation. Anti-*uc.339* siRNAs [si-*uc.339*(*1*) and si-*uc.339* *(2)*], anti-*ATP5G2* [si-*ATP5G2(1) and* si-*ATP5G2(2)*], anti-*CCNE2* [si-*CCNE2(1)* and si-*CCNE2(2)*], and a scrambled siRNA (si-*SCR*) were supplied by Ambion. siRNAs were transfected into cell lines at a final concentration of 50 nM by using Lipofectamine^®^ RNAi Max (Invitrogen) and according to the manufacturer’s instructions.

### Luciferase reporter assays

To analyze the direct targeting of *miR-339-3p*, *-663b-3p*, and *-95-5p* on the *CCNE2* mRNA, the LightSwitch 3′UTR vector containing the *CCNE2* 3′-UTR was purchased from the LightSwitch 3′UTR Reporter GoClone Collection of SwitchGear Genomics.

As a control vector, we performed a deletion of the miRNA-binding sites in *CCNE2* 3′-UTR vector with the mutagenesis primers listed in Supplementary Table 1 and by using the QuikChange II XL Site-Directed Mutagenesis kit (Stratagene), following the manufacturer’s instructions^59^.

The TP53 CS #1–4 containing fragments were cloned in the pGL4.23 [luc2/minP] vector (Promega), upstream of the minimal promoter for firefly luciferase. Cloning was successfully achieved by using the *Kpn*I and *Nhe*I restriction sites and the primers listed in Supplementary Table 1.

To normalize the luciferase reporter assay experiments, we co-transfected cells with the vectors containing the firefly luciferase gene, and with the pGL4.74 (hRluc/TK) vector with Lipofectamine^®^ 2000 (Invitrogen) as indicated by the supplier. The luciferase signal was analyzed with the Dual-Luciferase^®^Reporter Assay System (Promega) and read in a luciferase reporter assay system kit and Glomax^®^96 Microplate Luminometer (Promega), according to the operator’s manual.

### In situ hybridization

The formalin-fixed paraffin embedded tissue sections were dewaxed in xylenes and rehydrated through an ethanol dilution series^60^. Tissue sections were digested with 5 μg ml^−1^ proteinase K for 15 min at RT, and were then loaded onto Ventana Discovery Ultra for in situ hybridization analysis. The tissue slides were incubated with double-DIG labeled mercury LNA probe (Exiqon) for 2 h. The slides were treated with 3% H_2_O_2_ to inactivate endogenous peroxidase. Followed by a rabbit polyclonal anti-DIG antibody (Sigma, Cat.#: D7782, 1:10,000 dilution) and HRP conjugated secondary antibody (Ventana, Cat.#: 760–4311, ready to use) incubation, tyramine-conjugated fluorochrome (TSA) reaction was performed for 12 min. Finally, the slides were mounted with antifading ProLong Gold Solution (Life Technologies). For the chromogenic detection of miRNAs, miRNA signal was recognized by anti-DIG antibody, and alkaline phosphatase (AP) conjugated second antibody (Ventana, Cat.#: 760–4314, ready to use) using NBT-BCIP as the substrate. The following probes were used: *CCNE2*: 5′-TGCTCTTCGGTGGTGTCATAA-3’ and *uc.339*: 5′-AGATGGAGGATCGGTGTGAAA-3′. For *U6* control probe # 99002-15 (Exiqon) was used. For *miR-339-3p*, probe # 38578-15 (Exiqon) and for *miR-663b-3p*, probe # 21359-15 (Exiqon) were used. The *uc339* signal in ISH was amplified by anti-rab-HRP antibody and Tyramide signal amplification (TSA) system.

### Microarray profile and analysis

To determine the expression of mRNAs in A549 and LoVo cells stably infected with LV-E or LV-*uc.339* lentiviral vectors, the GeneChip® Human Genome U133 Plus 2.0 Array (Affymetrix) was used. Affymetrix CEL files were imported to the BRB software tools developed by Dr. Richard Simon and the BRB-ArrayTools Development Team, and the arrays were normalized using the robust multichip analysis (RMA) procedure. A filtering step was performed to remove the probe sets that did not show significant variation across the samples; a probe was excluded if less than 20% of expression data have at least a 1.5-fold change in either direction from gene’s median value or the percentage of data missing or filtered out exceeds 50%. Class comparisons were performed using the univariate *t*-test, and we included only those probes that had a *P*-value of <0.01.

### Subcellular fractionation assay

Fractionation assay was performed using the Thermo Scientific Subcellular Protein Fractionation Kit as per the manufacturer’s instructions. In brief, H460, H1299, and A549 cells were cultured in 100mm dish for a period of 72 h to confluence. To isolate the cytoplasmic and nuclear fractions, the cells were resuspended in ice-cold cytoplasmic extraction reagent I in the presence of protease inhibitors (Roche Life Sciences). After vigorous vortex, the cell lysates were kept on ice for 10 min followed by the addition of cytoplasmic extraction reagent II. After a short vigorous vortex, the cell lysates were kept on ice for 1 min. After centrifugation, the supernatant was collected as the cytoplasmic fraction. The leftover nuclear pellets were then resuspended in nuclear extraction buffer on ice for 40 min. The nuclear fraction (supernatant) was collected by centrifugation and was used for western blotting experiments. Tubulin and nucleophosmin-1 (B23) proteins were used as markers for cytoplasmic and nucleus fractions, respectively.

### Western blotting

For immunoblotting analysis, cells were lysed with complete RIPA buffer (Santa Cruz Biotechnologies) and cellular proteins were denaturated at 100 °C for 10 min. Overall, 50 μg of proteins were loaded on Criterion™ XT 4–20% Precast Gels (Bio-Rad), and transferred on Trans-Blot^®^Turbo™ Midi Nitrocellulose Transfer Pack membrane (Bio-Rad) by using a Trans-Blot^®^Turbo™ Transfer System (Bio-Rad), according to the manufacturer’s instructions. The membrane was stained with Ponceau S (Sigma-Aldrich) to make sure that equal amounts of proteins were loaded in each lane. The membranes were then incubated for 2 h at room temperature with T-PBS containing 5% non-fat dry milk. The membrane was probed overnight at 4 °C with the primary antibody, then horseradish peroxidase-conjugated secondary antibody (Dako Corporation) was added at a dilution of 1:5000. The following primary antibodies were used: anti-ATP5G2, rabbit polyclonal antibody (Thermo Scientific, Cat.#: PA5-25799) diluted 1:100, anti-CCNE2, rabbit monoclonal antibody (Abcam, Cat.#: ab32103) diluted 1:500, anti-PARP, rabbit polyclonal antibody (Cell Signaling, Cat.#: 9542) diluted 1:1000, anti-TP53 Ab-2, mouse monoclonal antibody (Thermo scientific, Cat.#: MS-105-P0) diluted 1:400, and anti-vinculin, mouse monoclonal antibody (Biohit, Cat.#: 610014) diluted 1:1000. The used secondary antibodies were: goat anti-mouse HRP conjugated (Santa Cruz Biotechnology, Cat.#: sc-2005) diluted 1:5000 and goat anti-rabbit HRP conjugated (Bethyl, Cat.#: A120-101P) diluted 1:5000.

The bound antibodies were detected by enhanced chemiluminescence and by using the SuperSignal West Femto Chemiluminescent Substrate (Thermo Scientific), according to the manufacturer’s instructions. The quantification of the chemiluminescent bands was determined by using the Quantity One software (Bio-Rad). All uncropped western blotting images are provided as Supplementary Figs. 12–33.

### Cell viability and cell-cycle assays

For cell viability assay, A549, H460, H1299, and LoVo cells and relative stably infected cells with LV-E or LV-*uc.339* were detached with trypsin after 72 h from plating or from the treatment (with si-*SCR* or si-*uc.339*), washed, and resuspended in PBS. An aliquot of the cell suspension was combined with an equal volume of 0.4% Trypan Blue and incubated for 8–10 min at room temperature. Total cell numbers and percentages of viable and non-viable stained cells were counted in a KOVA^®^Glasstic^®^ Slide counting chamber (Hycor Biomedical).

For cell-cycle analysis, the cells were harvested 72 h after treatment with si-*SCR* or si-*uc.339*, fixed in 70% ethanol, and stained in a solution containing 10 μg ml^−1^ of propidium iodide (Sigma-Aldrich), 10,000 U ml^−1^ of RNase (Sigma-Aldrich) and 0.01% of NP40 (Sigma-Aldrich). After 30–60 min, the samples were analyzed by flow cytometry using a BD FACSVantage™ cytofluorimeter (BD Biosciences). Data acquisition (10,000 events were collected for each sample) was performed by using the BD CellQuest™ Pro software (BD Biosciences), according to the manufacturer’s instructions. The data were elaborated using the ModFit LT™ software (Verity Software House), according to the manufacturer’s instructions and expressed as fractions of cells in the different cell-cycle phases.

### Cell migration assay

Wound healing assay was performed with ibidi (Martinsried, Germany) cell culture inserts with a defined cell-free gap. Overall, 3 × 10^5^ A549 cells infected with LV-E or LV-*uc.339* were suspended in 70 μl full media for each well of the insert, which had been previously placed in a 12-well cell culture dish. The cells were allowed to adhere in a 37 °C, 5% CO_2_ incubator for 4 h. The insert was carefully removed with sterile forceps. The cells were then washed with prewarmed media several times to remove all non-adherent cells. Phase contrast images (×4) were immediately taken and designated as time 0. The position of the field was waymarked. Eight and twenty-four hours later, the images were again obtained at the same location as time 0.

The images were then uploaded to the ibidi company website and analyzed with their Wim–Scratch Analysis program. The gap is 500 ± 50 μm and the distance is measured comparing to time 0 (500 μm), over time this number got smaller. Data are reported as percent of area of the initial cell-less gap. The experiment was conducted in triplicate.

### RNA stability assay

H1299 cells were grown until 70–80% confluence then treated with Actinomycin D (10 µg ml^−1^, Sigma-Aldrich) for the indicated times. RNA was extracted at each indicated time for qRT-PCR analysis. RNA was extracted using Trizol reagent (Invitrogen) and retro-transcribed using the SuperScript III Reverse Transcriptase (Thermo Fisher Scientific) following the manufacturer’s instruction. After the real-time PCR normalization was performed using three housekeeping genes (*HPRT*, *PGK1*, and *β-Actin*). mRNA level at each time point was calculated as percentage respect to the untreated cells.

### Prediction of the secondary structure of the ultraconserved RNA *uc.339*

This was conducted with two different RNA folding algorithms, RNAfold^61^ and CentroidFold^62^, to predict the consensus secondary structure. The default parameters were used unless otherwise stated. We attempted to choose the most destructive mutations (disruption of the conserved stem structure of RNA). The consensus Watson–Crick base pairs outside the binding site of *miR-339-3p*, *miR-663b-3p*, and *miR-95-5p* were evaluated for their effect on the structure stability of *uc.339*. The deletions were selected based on our in silico modeling such that they would alter the local folding around the corresponding miRNA-binding site and release the miRNA from trapping. Using such rationale and strategy, we designed Δ117-CTCCC-121 for *miR-339-3p* TRE and Δ249-TCCCG-253 for *miR-663b-3p* TRE deletions.

### Generation of CRISPR/Cas9 *uc.339* knockout NCI-H1299 cell line for *miR-339-3p* MBE

One pair of gRNAs were designed by GenScript flanking 24 bp of the core sequence “CCGGCTCCCGGGCCCCGCGGCGCC”. -g1: CGATTAAATGGAGGAGCTCG and g20: TCCTTCCCGCCGCCCCCTCC. gRNAs were cloned in the pSpCas9(BB)-2A-GFP(PX458) plasmid (GenScript), and wild-type H1299 cells were transfected with Lipofectamine 2000 (Invitrogen). The deletion of the *miR-339-3p* MBE in the *uc.339* gene was confirmed by TA cloning and DNA sequencing. Clone g1&g20-20 (Clone 20) was identified as a heterozygous knockout clone with deletion of the core sequence on one allele and reversion of core sequence on the other allele. Clone g1&g20-102 (Clone 102) was identified as a homozygous knockout clone with deletion of the core sequence. Both clones were tested to be mycoplasma-free and banked.

### Colony-forming assay

The cells were seeded in 6-well plate at 2000 cells per well or 5000 cells per well, and allowed to grow for 10 days at 37 °C in a 5% CO_2_ humidified incubator. The cells were fixed with 100% methanol at room temperature for 20 min, stained with 0.5% crystal violet in 25% methanol at room temperature for 5 min, and washed with water until excess dye is removed. Images were captured with a digital camera, and the number of colonies in each well was counted with the image processing program ImageJ. The experiment was repeated in triplicate, and data were presented as mean ±  s.d. with individual value shown. Unpaired *t*-test was used to analyze the difference between the treatment and control groups. A *P*-value <0.05 was considered significant. An independent experiment with 5000 cells per well repeated in triplicate showed similar results.

### Animal experiments

Four-week-old female nude mice (NU/J, Jax #002019,) were purchased from Jackson Laboratories (total *n* = 14). At five weeks of age, all mice were irradiated with a 200 cGy total body irradiation at the Animal Care Facility of the Saban Research Institute of Children’s Hospital Los Angeles. The following day, seven mice were injected subcutaneously with 5 × 10^6^ A549 viable cells stably infected with LV-E and seven mice were injected subcutaneously with 5 × 10^6^ A549 viable cells stably infected with LV-*uc.339*. Tumor diameters were measured after 8 days from the injection and then every 3 days. At 29 days after the injection, the mice were killed, a necropsy performed, and the tumors were excised, measured, and photographed. The tumor volumes were determined by using the equation *V* (mm^3^) = *L* × *W* ^2^/2, where *L* is the largest diameter and *W* is the perpendicular diameter. From about 1/10 of the excised tumor, total RNA was extracted and *uc.339* expression was determined by qRT-PCR in triplicate, per each mouse and as described in the Supplementary methods.

For the H1299 CRISPR in vivo experiments, a total of 15 NSG mice (NOD.Cg-Prkdcscid Il2rgtm1Wjl/SzJ, Jax #005557), aged 4–6 weeks, randomized to an average age of 5 weeks at the start of the experiment, underwent total body irradiation (200 cGy) on day 1 and were separated into three groups (*n* = 5 per group, 3 F/2 M (group 1, 2) resp. 2 F/3 M (group 3)). After 24 h (day 0) the animals were injected subcutaneously with tumor cells at a concentration of 4 × 10^6^/animal as follows: group 1: H1299 clone #18; group 2: H1299 clone #20; and group 3: H1299 clone #102.

Tumors were palpable on day 7, and were measured via caliper every 3rd day from thereon. The tumor volume was calculated according to the ellipsoid formula (*W* ^2^ × *L *× π)/6. On day 25, the animals were killed and the tumors extracted and analyzed.

All procedures used in this study were complied with federal guidelines and were approved by the Institutional Animal Care and Use Committee at Children’s Hospital Los Angeles (IACUC #352-13, IACUC #352-16).

### Statistical analysis of data

Statistical data are presented as mean ± standard deviation (s.d.) of experiments conducted in triplicate, unless otherwise specified. Significance was calculated by two-tailed *t-test* for two group comparisons, while ANOVA test with Bonferroni correction was used for multiple group comparisons. A *P*-value <0.05 was considered statistically significant. To visualize the association between overall survival and *uc.339* expression, we used the log-rank test to find the point (cut-off) with the most significant (lowest log-rank test *P*-value) split in high vs low RNA level groups. The Kaplan–Meier method was used to generate survival curves for the optimal cut-off (0.56). Survival analysis was performed in R (version 3.2.5) (http://www.r-project.org/) and the statistical significance was defined as a *P*-value <0.05.

### Data availability

All data were submitted using MIAMExpress to the ArrayExpress database and can be retrieved using the accession number GSE70540.

The data that support the findings of this study are available from the corresponding authors upon reasonable request.

## Electronic supplementary material

Supplementary Information
