# Publisher Correction: Transcribed ultraconserved region 339 promotes carcinogenesis by modulating tumor suppressor microRNAs

**DOI:** 10.1038/s41467-017-02485-1

**Published:** 2018-01-08

**Authors:** Ivan Vannini, Petra M. Wise, Kishore B. Challagundla, Meropi Plousiou, Mirco Raffini, Erika Bandini, Francesca Fanini, Giorgia Paliaga, Melissa Crawford, Manuela Ferracin, Cristina Ivan, Linda Fabris, Ramana V. Davuluri, Zhiyi Guo, Maria Angelica Cortez, Xinna Zhang, Lu Chen, Shuxing Zhang, Cecilia Fernandez-Cymering, Leng Han, Silvia Carloni, Samanta Salvi, Hui Ling, Mariam Murtadha, Paolo Neviani, Barbara J. Gitlitz, Ite A. Laird-Offringa, Patrick Nana-Sinkam, Massimo Negrini, Han Liang, Dino Amadori, Amelia Cimmino, George A. Calin, Muller Fabbri

**Affiliations:** 1 Istituto Scientifico Romagnolo per lo Studio e la Cura dei Tumori (IRST) S.r.l., IRCCS, Gene Therapy Unit, Meldola, FC 47014 Italy; 2 Departments of Pediatrics and Molecular Microbiology & Immunology, Norris Comprehensive Cancer Center, Keck School of Medicine, University of Southern California, Children’s Center for Cancer and Blood Diseases and The Saban Research Institute, Children’s Hospital Los Angeles, Los Angeles, CA 90027 USA; 3 Department of Biochemistry & Molecular Biology, University of Nebraska Medical Center, 985870 Nebraska Medical Center, Omaha, NE 68198 USA; 4 Department of Internal Medicine, Division of Pulmonary, Allergy, Critical Care and Sleep Medicine, The Ohio State University, Columbus, OH 43210 USA; 5 Department of Experimental, Diagnostic and Specialty Medicine- DIMES, University of Bologna, Bologna, 40126 Italy; 6 Department of Experimental Therapeutics, The University of Texas MD Anderson Cancer Center, Houston, TX 77030 USA; 7 The Center for RNA Interference and Non-coding RNAs, The University of Texas MD Anderson Cancer Center, Houston, TX USA; 8 Departments of Preventive Medicine and Neurological Surgery, Northwestern University-Feinberg School of Medicine, Chicago, IL 60611 USA; 9 Department of Experimental Radiation Oncology, The University of Texas MD Anderson Cancer Center, Houston, TX 77030 USA; 10 Department of Gynecologic Oncology and Reproductive Medicine, The University of Texas MD Anderson Cancer Center, Houston, TX 77030 USA; 11 Integrated Molecular Discovery Laboratory, Department of Experimental Therapeutics, The University of Texas MD Anderson Cancer Center, Houston, TX 77030 USA; 12 Department of Molecular Virology, Immunology and Medical Genetics, Comprehensive Cancer Center, Ohio State University, Columbus, OH 43210 USA; 13 Department of Biochemistry and Molecular Biology, McGovern Medical School, The University of Texas Health Science Center at Houston, Houston, TX 77030 USA; 14 Istituto Scientifico Romagnolo per lo Studio e la Cura dei Tumori (IRST) S.r.l., IRCCS, Biosciences Laboratory Unit, Meldola, FC 47014 Italy; 15 Division of Medical Oncology, Norris Comprehensive Cancer Center, Keck School of Medicine, University of Southern California, Los Angeles, CA 90033 USA; 16 Departments of Surgery and of Biochemistry and Molecular Medicine, Norris Comprehensive Cancer Center, Keck School of Medicine, University of Southern California, Los Angeles, CA 90033 USA; 17 Division of Pulmonary Diseases and Critical Care Medicine, Virginia Commonwealth University, Richmond, VA 23298 USA; 18 Department of Morphology, Surgery and Experimental Medicine and Laboratory for Technologies of Advanced Therapies (LTTA), University of Ferrara, Ferrara, 44121 Italy; 19 Department of Bioinformatics and Computational Biology, The University of Texas MD Anderson Cancer Center, Houston, TX 77030 USA; 20 Department of Oncology Unit, Istituto Scientifico Romagnolo per lo Studio e la Cura dei Tumori (IRST) S.r.l., IRCCS, Meldola, FC 47014 Italy; 21 Institute of Genetics and Biophysics, National Research Council, Naples, 80131 Italy; 22 Present Address: Division of Pre-Clinical Innovation, National Center for Advancing Translational Sciences (NCATS), Rockville, MD 20850 USA

Correction to: *Nature Communications* 10.1038/s41467-017-01562-9, published online 27 November 2017

In the originally published version of this Article, the positions of the final two authors in the author list were inadvertently inverted during the production process. This error has now been corrected in both the PDF and HTML versions of the Article.

