# Supplementary material for: Transcribed ultraconserved region 339 promotes carcinogenesis by modulating tumor suppressor microRNAs
Source: Nat Commun. 2017 Nov 27;8:1801. doi: 10.1038/s41467-017-01562-9 (PMC5703849; doi:10.1038/s41467-017-01562-9)
Supplement: Supplementary file 1 — Supplementary Information [file 41467_2017_1562_MOESM1_ESM.pdf]

SUPPLEMENTARY FIGURES

Supplementary Figure 1.

a

Absolute expression level (Copies / RNA ngr)				
**Gene**	**A549**	**H460**	**H1299**	**LoVo**
uc.339	0.356	0.839	1.416	0.528
RNU44	3043.619	6081.619	2706.3	3238.144
miR-339-3p	531.316	460.815	346.658	332.35
miR-663-3p	171.299	262.117	167.346	236.703
miR-95-5p	12.447	21.402	6.817	11.405
RNU44	3356.048	7087.494	4763.567	5702.51
CCNE2	2.005	13.076	21.146	55.488
HPRT1	5870.238	6985.85	8161.719	26830.5

b

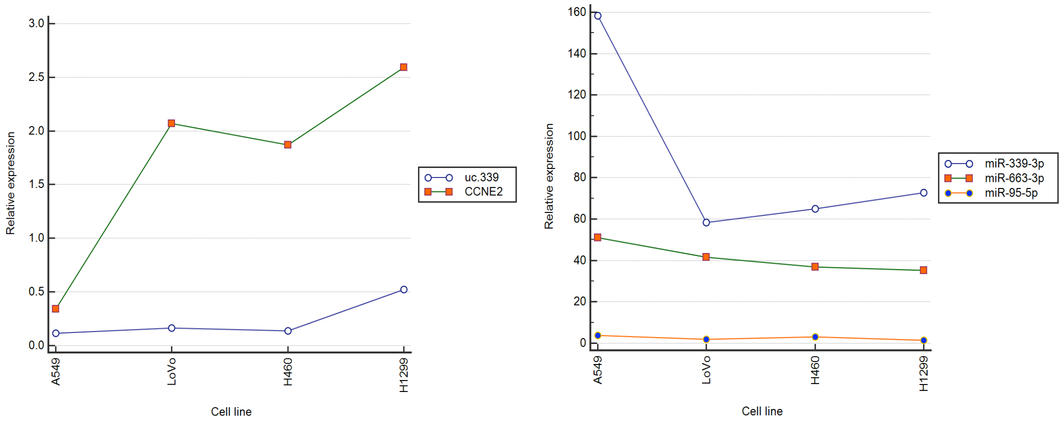

	A549	H460	H1299	LoVo
**uc.339 / CCNE2**	0.34	0.07	0.20	0.08
**CCNE2 / uc.339**	2.92	13.57	4.95	12.70

c

ACGCAGCACGAGAAAGACGGGAAAAGCTCACCAAACCCCATAAAAAATAAAAAATAAAAAGAAAGCCGCGGCTG
TAAATAAAGGCAAATCCGCCACTCTCTGAGGGGCCGTTTGTCCTCCCCTCCTTGGCCCCGTCCTTCCCGCCGCC
CCCTCCCGGCTCCCGGGCCCCGCGGCGCCCCGGCCCCGAGCTCCTCCATTTAATCGGATTTGGGAGAAGGGGAG
GATAAATCACGGCAGCAGCTTTACGGTCCCGGAGGAGAGGCGAGCCGCAGACAGGCACACCCCGGCCGGCGATA
AAAACCGCCGCTGAAAGCCCACGGAGCAATTTCCCGGGACCCCGAGCGACGCCATTACAGGAATGTAATTTTGC
CCGGATGAGGCCCCGAGTTTAATTATCCTCGCGGAGGAATTTCAATGCGGCCAATCCATCTTGCAGGCGGGCGG
CAGAGGGATTTATATGGCCCGTTATTTCACACCGATCCTCCATCTGCATTTTTATGGCCCTGAGCTCCTGAAA
GGGAGGGGAAGGAGTGGGGAGAAATAGGAGAAGCGGGCGAGAAGAGGAGGGTGGTAGACTGCCTGTCTTTCCTA
AATTCGCATTTCCAGCTCCATCAGGATTCTCACAGGTTCCTCTGAAATCCCCAAATCTCCAGTTTTAGTTGTTG
ATTGCGCCCCGAGCGGGCAGGTACGTGGGAAATGGAACTGGGATCTTTGGGAATCAATCAACAGGTATTTATCA
AGTACCAAGTGGTGCCAGGCACTGTGGAGATGCTGTTAGGAGAAAGACAGGCTAGCAAAGACACAAGTTTATCC
CCTGAGGTCTCCAGACATCTCCATTCCCACCAAAA

Cloning of *uc.339* and endogenous expression in cell lines.

(**a**) Endogenous absolute expression levels of *uc.339, miR-339, miR-663b, miR-95, CCNE2* in A549, H460, H1299, and LoVo cells detected as copies $ngRNA^{-1}$ input by QuantStudio3D digital PCR system.

(**b**) Endogenous relative expression levels of *uc.339, and CCNE2* (left), and of *miR-339, miR-663b, miR-95* (right) in A549, H460, H1299, and LoVo cells. The expression of *uc.339, miR-339, miR-663b, miR-95 and CCNE2* was determined using QuantStudio3D digital PCR system and normalized to *RNU44 (miRNAs and UCR) or HPRT1* (CCNE2) reference genes. The ratio of *CCNE2 / uc.339 expression* in A549, H460, H1299, LoVo is reported in the lower part of the Figure. Numbers have been rounded to 2 decimals.

(**c**) Sequence of the *uc.339* transcript, as obtained by RACE. The red bases indicate the overlapping sequence with the gene *ATP5G2*, the underlined bases indicate the sequence as reported by Bejerano *et al.* [1], while the highlighted bases represent the interaction consensus sequence of *miR-339, -663b, -95* in light blue, green and purple, respectively.

Supplementary Figure 2.

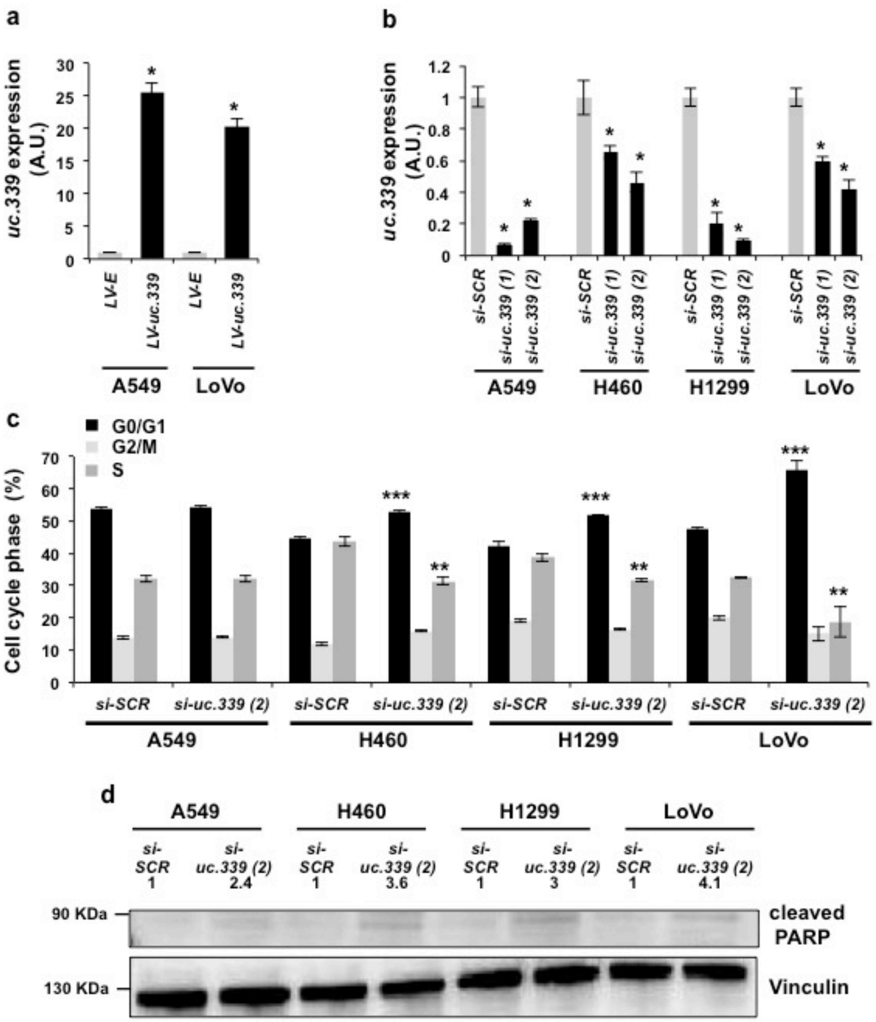

***uc.339* promotes NSCLC growth in cell lines.**

(**a**) qRT-PCR for *uc.339* in A549 and LoVo infected with a lentiviral vector over-expressing *uc.339* (LV-*uc.339*) or its empty vector counterpart (LV-E) and detected after 72h.

(**b**) qRT-PCR for *uc.339* in A549, H460, H1299 and LoVo cells transfected with two different anti-*uc.339* siRNAs [si-*uc.339 (1) and si-uc.339 (2)*], or an anti-*scrambled* siRNA (si-*SCR*) for 72h. The expression of *uc.339* has been normalized to *RNU44* and the results are presented as mean ± s.d. of experiments conducted in triplicate and normalized to si-*SCR*. *Paired t-test *P*-value<0.05.

(**c**) Cell cycle analysis (shown as the percentage of cells in G_0/G_1 or G_2/M or S phase of the cell cycle) conducted by cytofluorimetry with propidium iodide staining in A549, H460, H1299 and LoVo cells transfected with si-*uc.339 (2)* or si-*SCR* for 72h. Data are presented as mean ± s.d. of experiments conducted in triplicate. ** Paired t-test *P*-value <0.01. *** Paired t-test *P*-value <0.001.

(**d**) Immunoblotting for cleaved PARP and Vinculin in A549, H460, H1299 and LoVo cells transfected with si-*uc.339 (2)* or si-*SCR* for 72h.

Supplementary Figure 3.

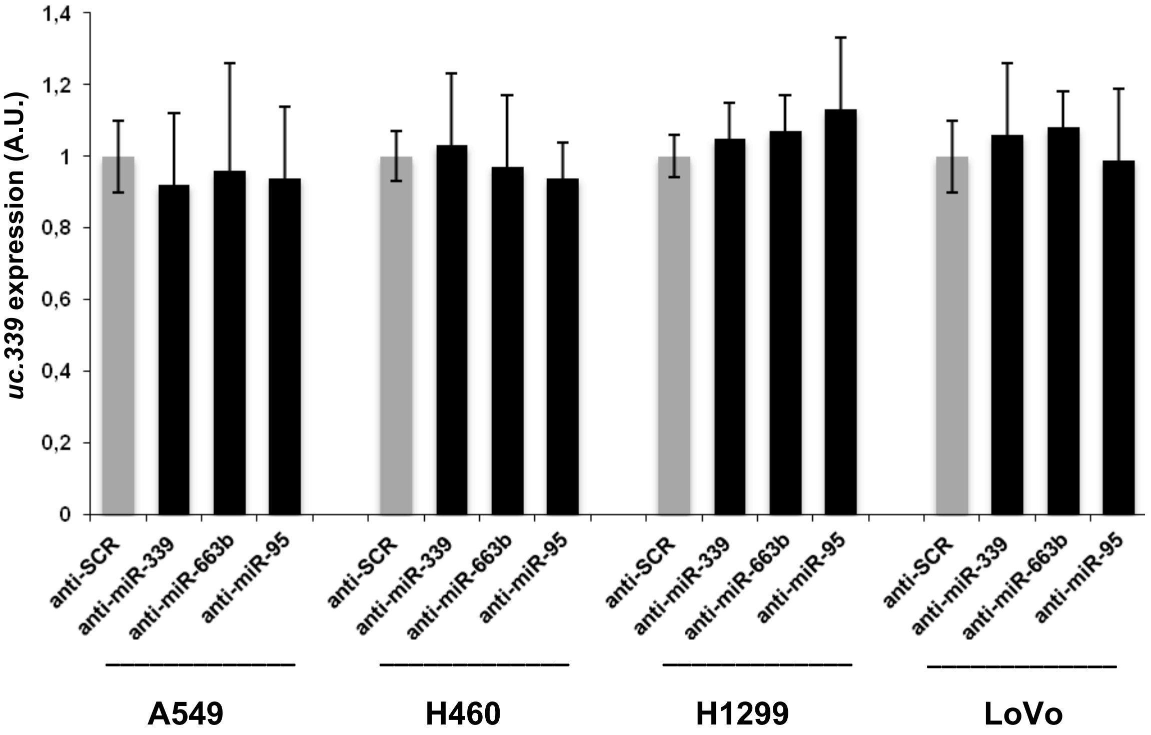

miRNA downregulation by anti-miRNAs does not significantly affect *uc.339* levels.
qRT-PCR for *uc.339* in A549, H460, H1299 and LoVo cells transfected with anti-scrambled (anti-*SCR*) or anti-*miR-339*, anti-*miR-663b*, and anti-*miR-95* for 48h. Data presented as mean ± s.d. of experiments conducted in triplicate.

Supplementary Figure 4.

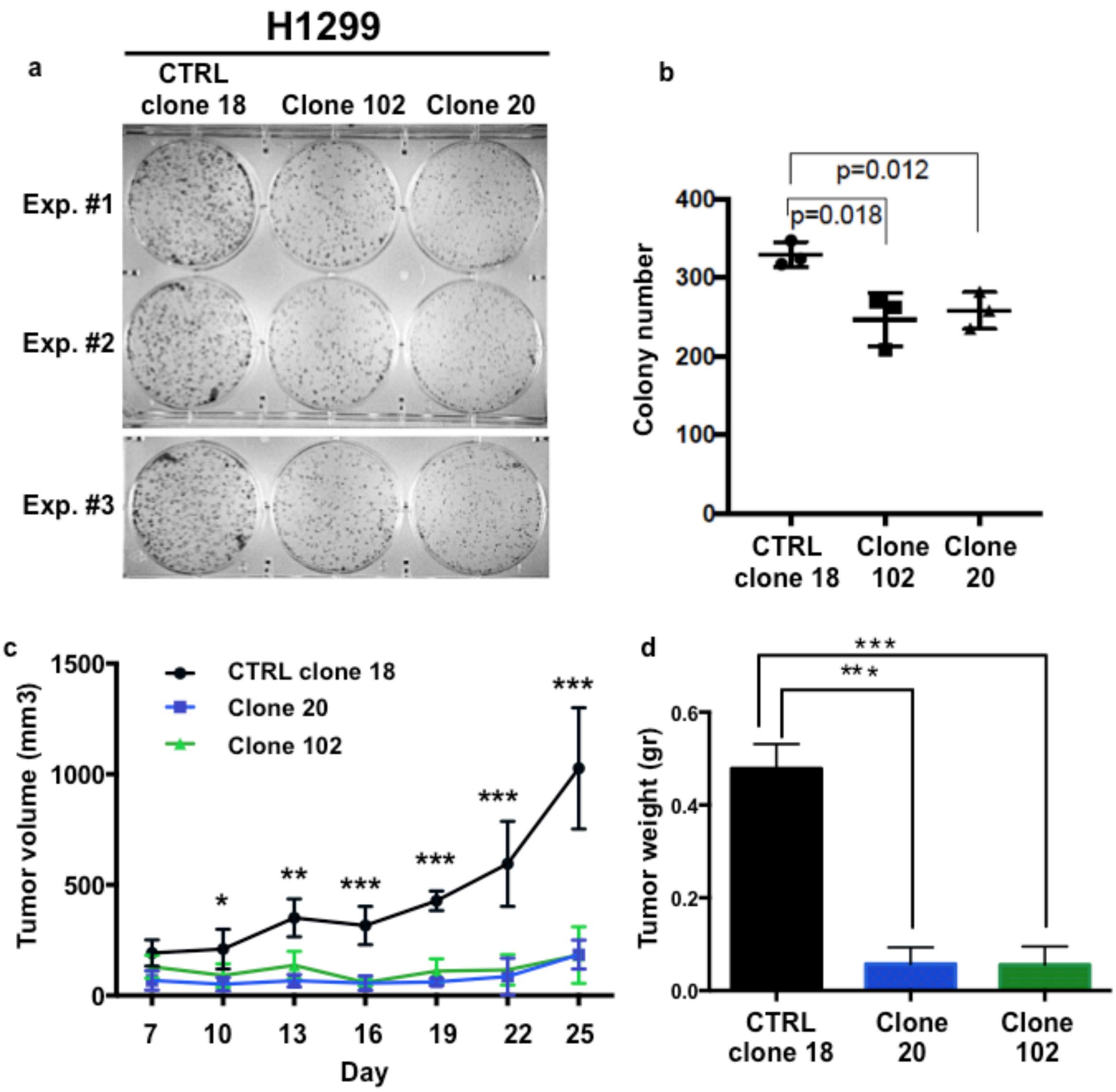

CRISPR clones of H1299 cell expressing endogenous *uc.339* with deletion of *miR-339* binding site have a reduced ability to form clones *in vitro* and grow less *in vivo*.

(**a**) Clone forming assay images at 48h from 3 different experiments with H1299 cells expressing wild-type endogenous *uc.339* (CTRL clone 18) or with deletion of the binding site for *miR-339* in the *uc.339* transcript (clones 102 and 20).

(**b**) Quantification of the colony formed by the CRISPR clones of experiments in **a**, as obtained by the Image J software.

(**c**) Tumor volumes measured at the indicated time points in nude mice (*n*=5/group) injected sub-cutaneously with H1299 clones 18, 20, or 102. *Paired t-test *P*-value<0.05. ** Paired t-test *P*-value <0.01. *** Paired t-test *P*-value <0.001.

(**d**) Tumor weights collected *ex vivo* from the same animals in **c**, after euthanizing the animals on day 25. *** Paired t-test *P*-value <0.001.

Supplementary Figure 5.

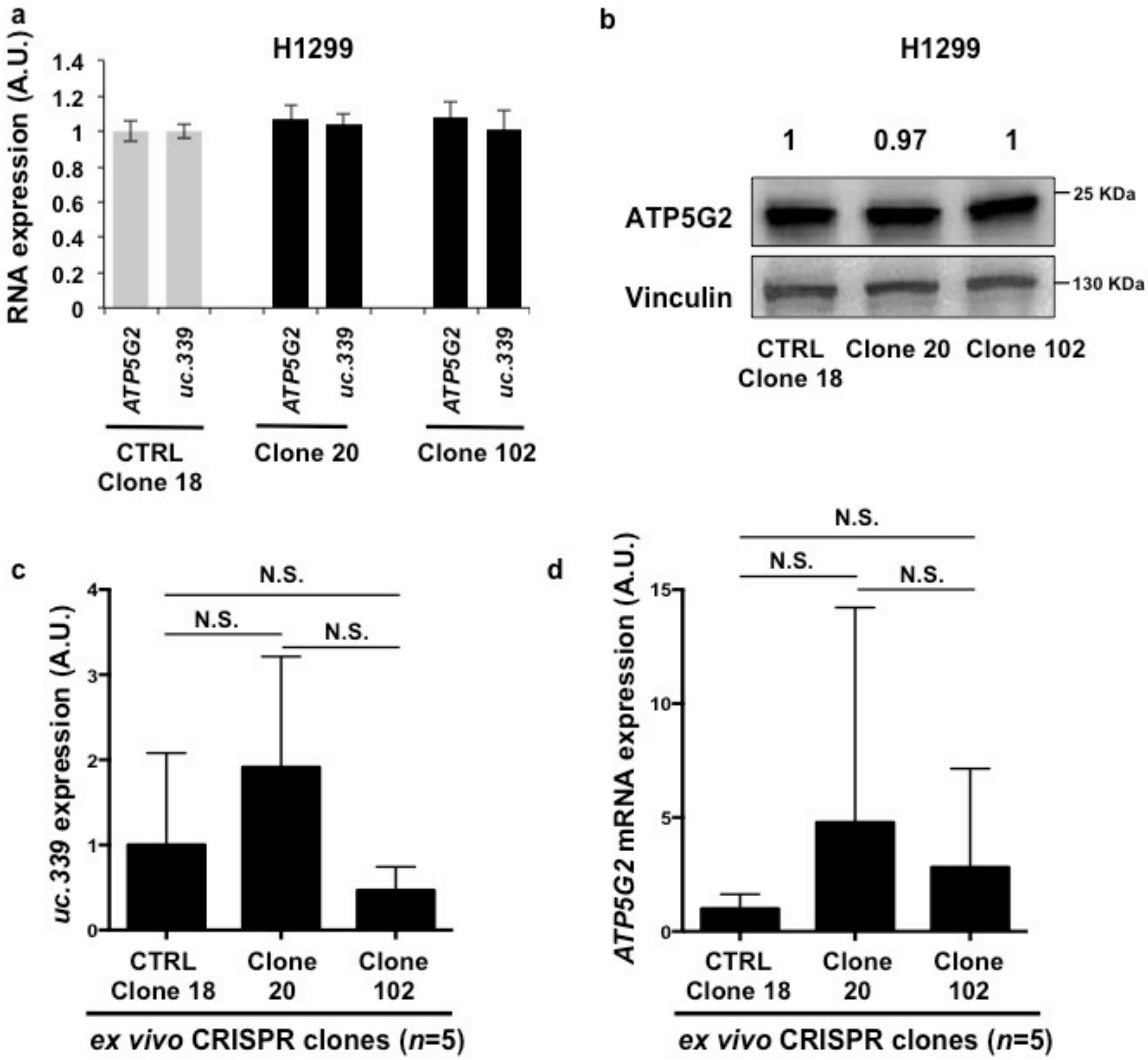

***ATP5G2* expression is not altered in H1299 CRISPR clones.**
(**a**) qRT-PCR for *ATP5G2* and *uc.339* in H1299 cells wild type (CTRL clone 18) or with CRISPR deletion of the *miR-339* binding site in the endogenous *uc.339* transcript (clones 20 and 102).
(**b**) Immunoblotting for ATP5G2 and Vinculin (housekeeping gene) in H1299 cell CRISPR clones 18, 20 and 102.
(**c**) qRT-PCR for *uc.339* in *ex vivo* xenografts from the mice of the experiment in **Supplementary Fig. 4c,d**.
(**d**) qRT-PCR for *ATP5G2* in *ex vivo* xenografts from the mice of the experiment in **Supplementary Fig. 4c,d.**

Supplementary Figure 6.

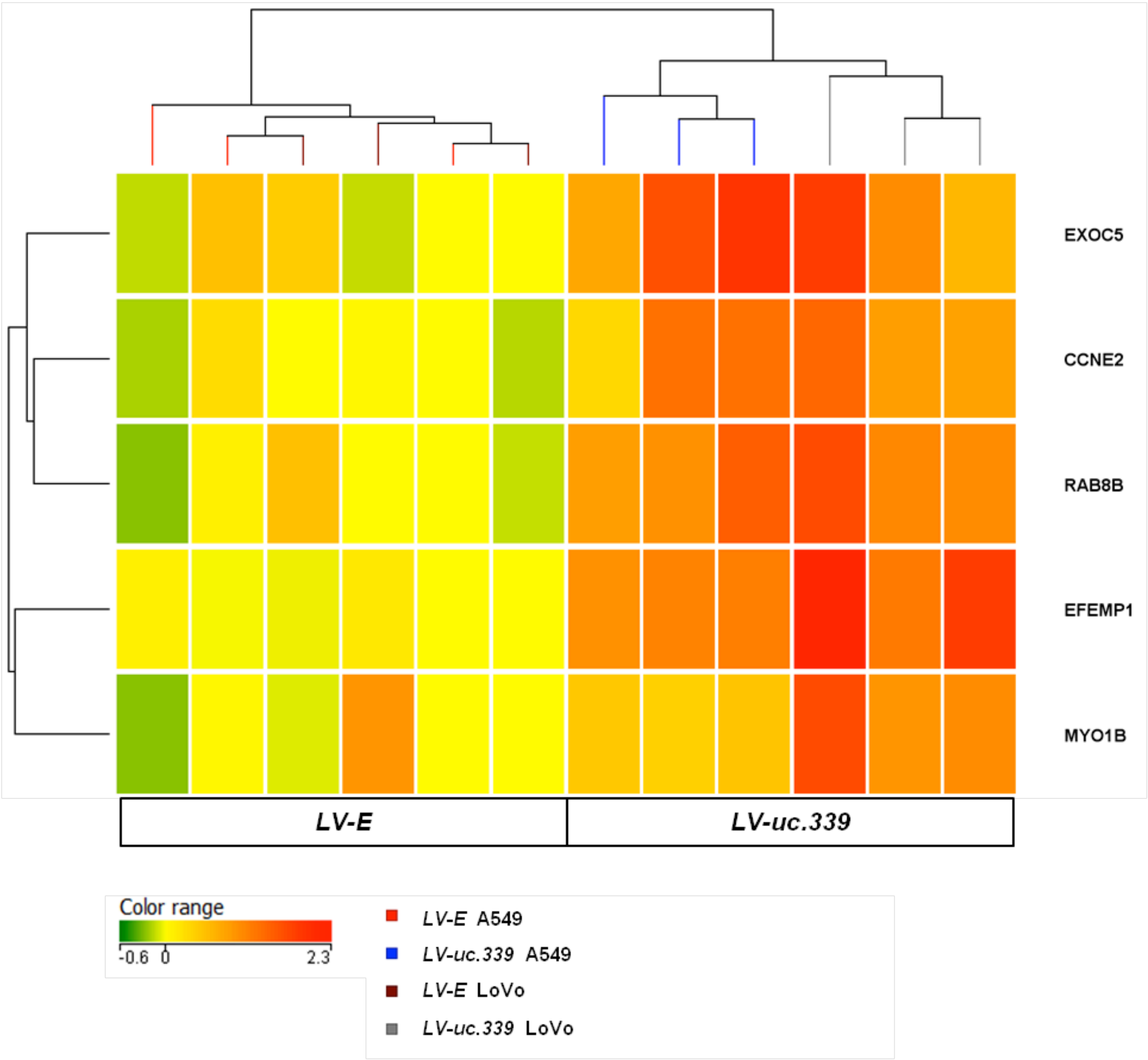

Up-regulated genes in A549 and LoVo cells over-expressing *uc.339.*

Heat map for the top 5 up-regulated genes (fold change >2, *P*<0.05) common to A549 and LoVo cells over-expressing *uc.339* by lentiviral infection compared to the same cell line infected with an empty lentiviral vector. The differential mRNA expression has been assessed by Affymetrix microarray. In A549 cells 936 genes were significantly (*P*<0.05) up-regulated and 1675 down-regulated in LV-*uc.339* vs LV-E, whereas in LoVo cells, 945 genes were up-regulated and 619 down-regulated in LV-*uc.339* vs LV-E. This heatmap highlights the top 5 genes commonly up-regulated in both cell lines. The colors of the legend in the figure refer to the vertical lines above the clusters.

Supplementary Figure 7.

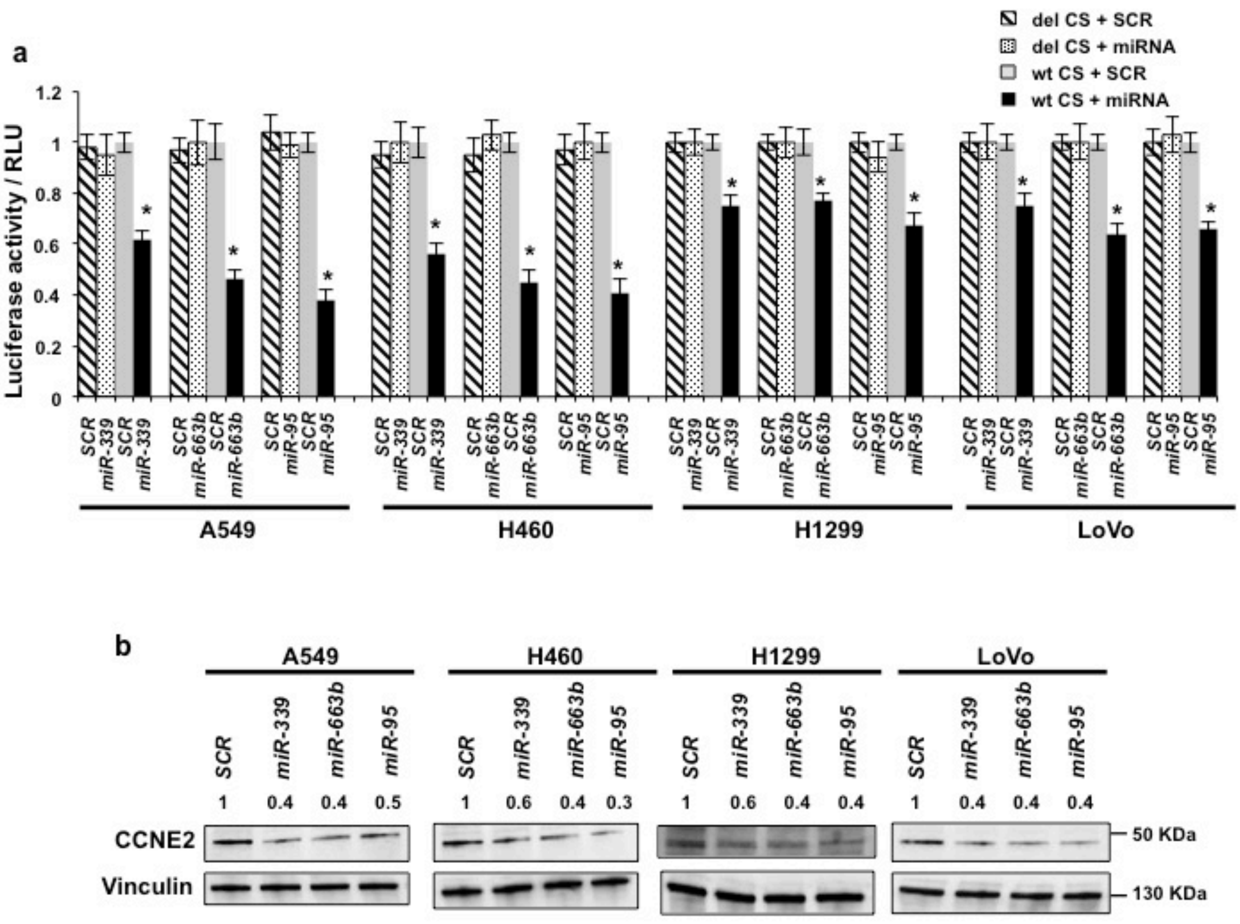

***miR-339, -663b, -95* directly target CCNE2 in cancer cells.**

(**a**) Luciferase reporter assay in A549, H460, H1299 and LoVo cells co-transfected with *miR-339, -663b* and *-95* or a scrambled miRNA (SCR) and a reporter plasmid containing the 3'-UTR complementary site (CS) for the miRNAs on the mRNA of the *CCNE2* gene (wt CS) or a mutant in which the binding site has been deleted (del CS). Luciferase activity has been normalized to Renilla (RLU) and the results are presented as normalized to SCR. Data are shown as mean ± s.d. of experiments conducted in triplicate. *Paired t-test *P*-value<0.05.

(**b**) Immunoblotting for CCNE2 and Vinculin in A549, H460, H1299 and LoVo cells transfected with *miR-339, -663b* and *-95* or a scrambled miRNA (SCR) for 72h. The numbers above the bands represent the quantification of the band intensity, calculated with Quantity One software and normalized to Vinculin and SCR.

Supplementary Figure 8.

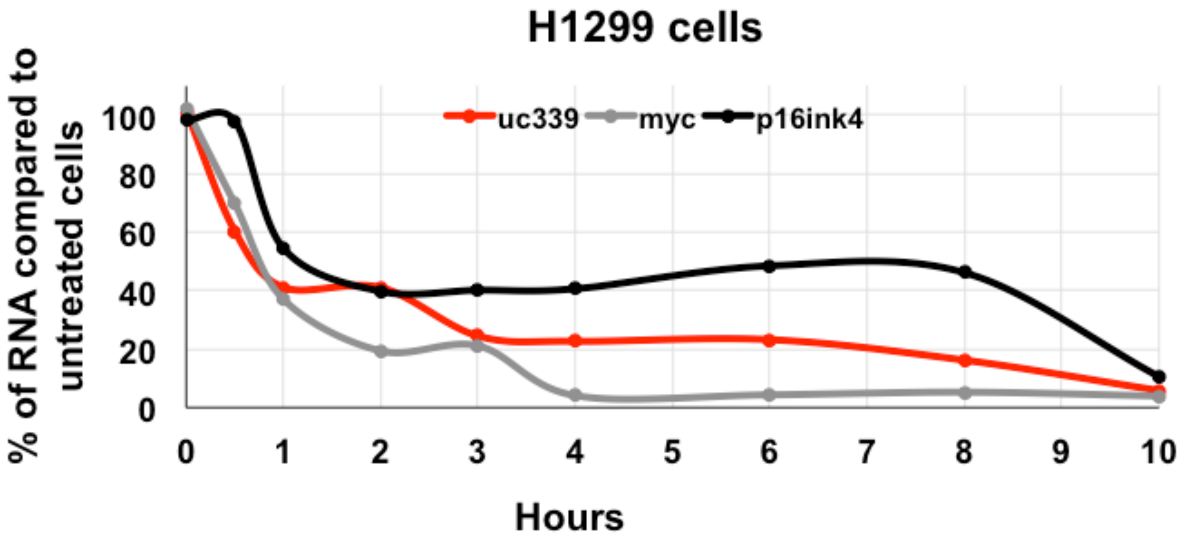

The *uc.339* transcript is stable.
RNA stability assay in H1299 cells treated with Actinomycin D (ActD) for the indicated time points. The expressions of *uc.339*, *MYC* and *p16ink4* were detected by qRT-PCR in ActD treated and untreated cells and the qRT-PCR were normalized with three housekeeping genes (*HPRT*, *PGK1*, and b-*actin*). The RNA expression at each time point is reported as percentage of RNA in ActD-treated cells compared to untreated cells.

Supplementary Figure 9.

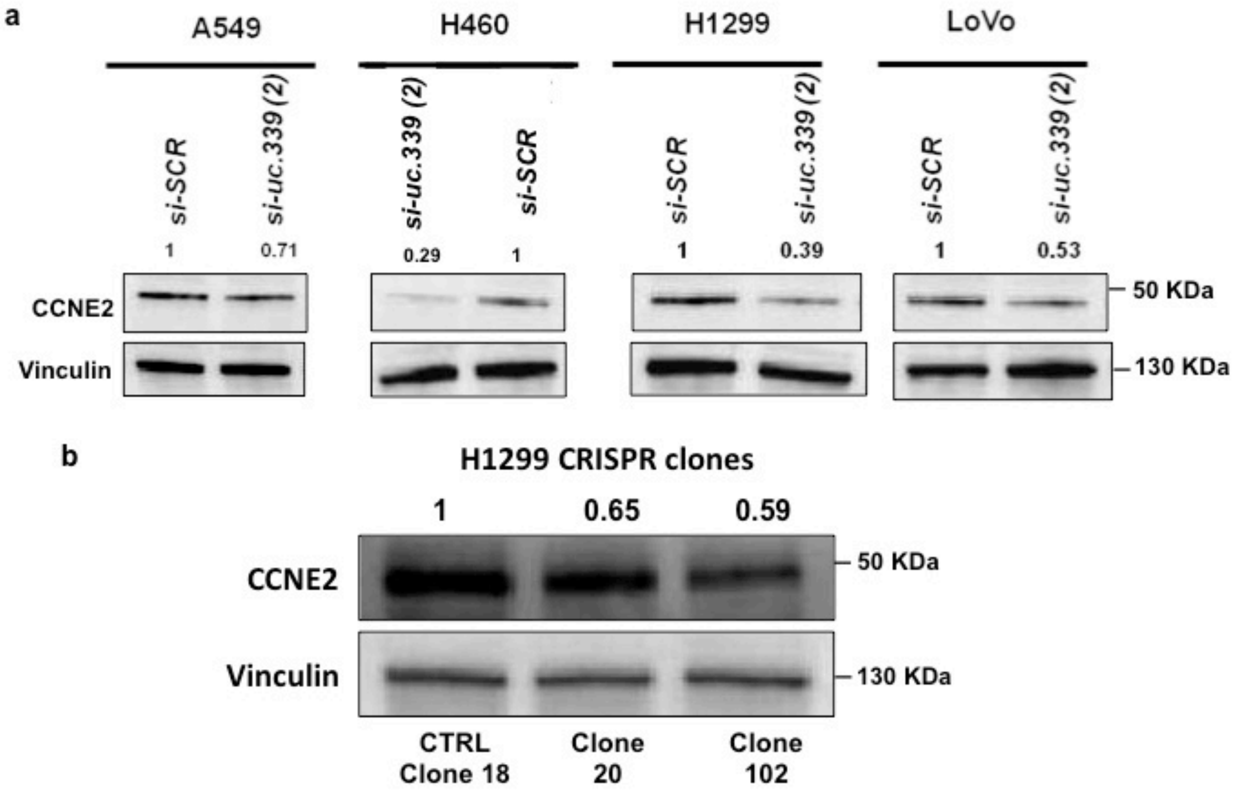

Expression of protein CCNE2 in A549, H460, H1299, and LoVo cells silenced with an additional siRNA anti-*uc.339* and in H1299 CRISPR clones.

(**a**) Immunoblotting for CCNE2 and Vinculin in A549, H460, H1299, and LoVo cells transfected with si-*uc.339 (2)* or si-*SCR* for 72h. The numbers above each lane represent a quantification of the band intensity, normalized to the corresponding Vinculin band and to *si-SCR*.

(**b**) Immunoblotting for CCNE2 and Vinculin in H1299 CRISPR clone 18 (wild-type expression of *uc.339*), clone 20 and clone 102 (with endogenous, genomic deletion of the *miR-339* MBE in the *uc.339* gene and transcript). The numbers above each lane represent a quantification of the band intensity, normalized to the corresponding Vinculin band and to CTRL Clone 18.

Supplementary Figure 10.

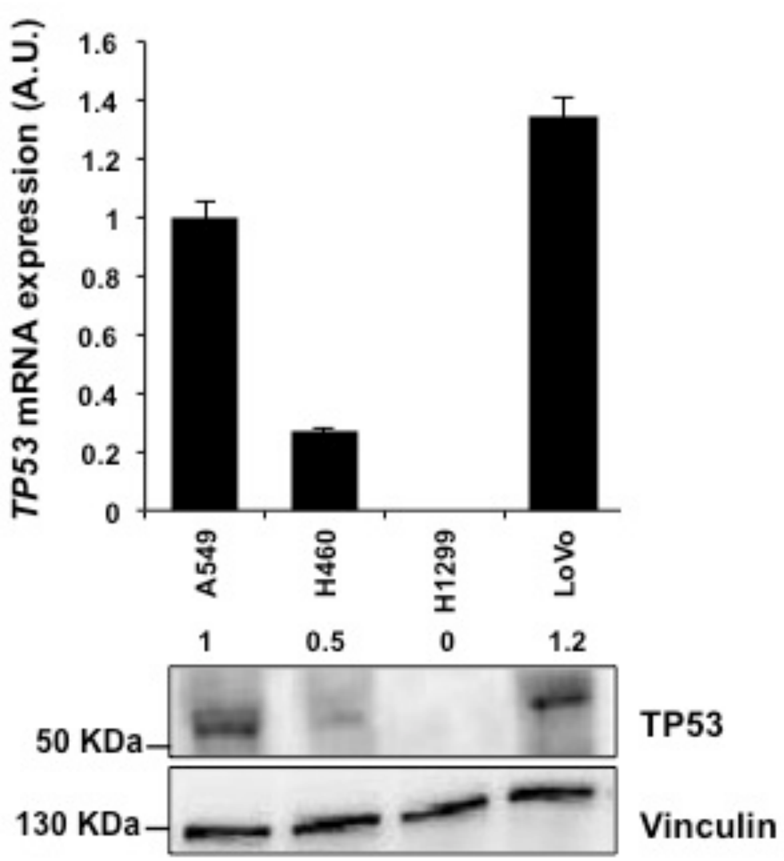

Endogenous *TP53* expression in the cell lines used in this study.
(**upper**) qRT-PCR for *TP53* in A549, H460, H1299 and LoVo cells. The expression of *TP53* has been normalized to *HPRT1* and the results are presented as mean ± s.d. of experiments conducted in triplicate relative to A549 cells. (**lower**) Immunoblotting for TP53 and Vinculin in A549, H460, H1299 and LoVo cells. The expression of TP53 has been normalized to A549.

Supplementary Figure 11.

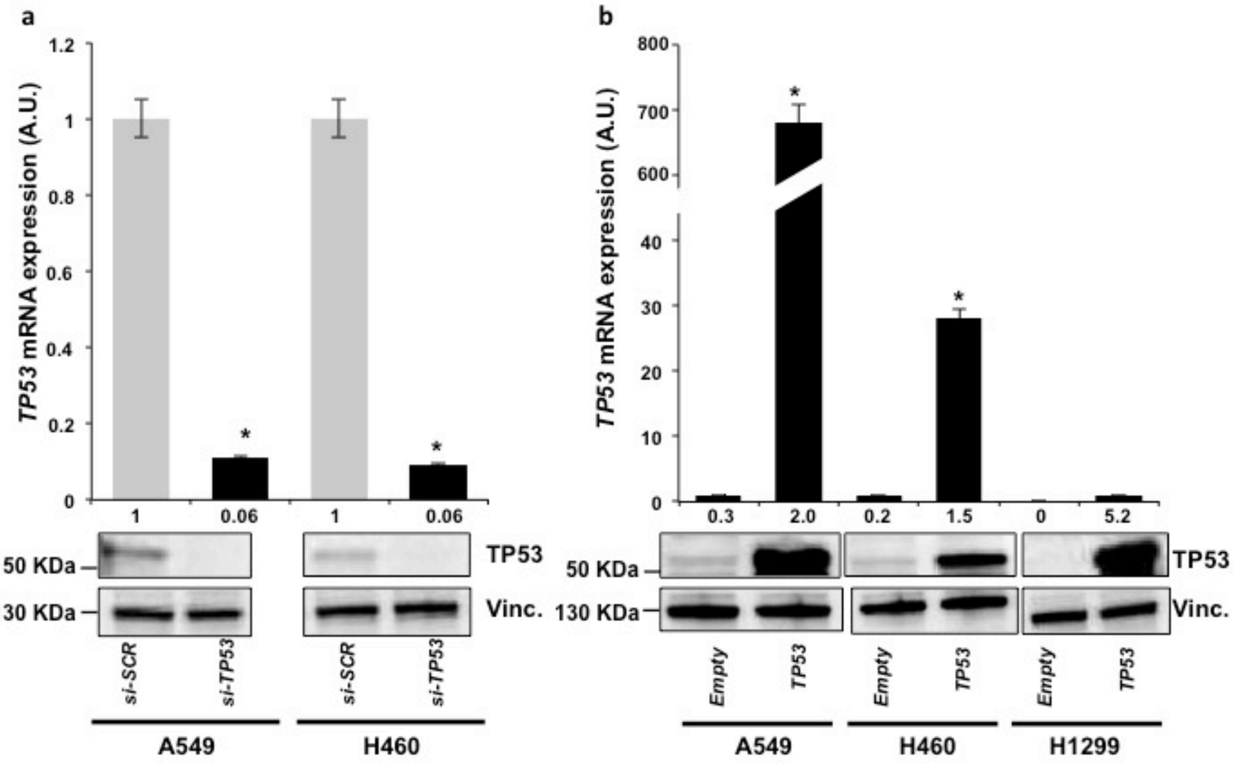

Silencing and re-expression of *TP53*.

(**a**) qRT-PCR and immunoblotting for *TP53* in A549 and H460 cells transfected with an anti-*TP53* siRNA (si-*TP53*) or its anti-scrambled siRNA (si-*SCR*) for 72h. In qRT-PCR, the expression of *TP53* has been normalized to *HPRT1* and the results are presented as mean ± s.d. of experiments conducted in triplicate and normalized to si-*SCR*. *Paired t-test *P*-value<0.05. In immunoblotting the expression of TP53 protein has been normalized to vinculin and si-SCR.

(**b**) qRT-PCR and immunoblotting for *TP53* in A549, H460 and H1299 cells transfected with a plasmid expressing *TP53* or its empty plasmid counterpart (Empty) for 72h. The expression of *TP53* has been normalized to *HPRT1* and the results are presented as mean ± s.d. of experiments conducted in triplicate and normalized to Empty. *Paired t-test *P*-value<0.05. In immunoblotting the expression of TP53 protein has been normalized to Vinculin.

Supplementary Figure 12.

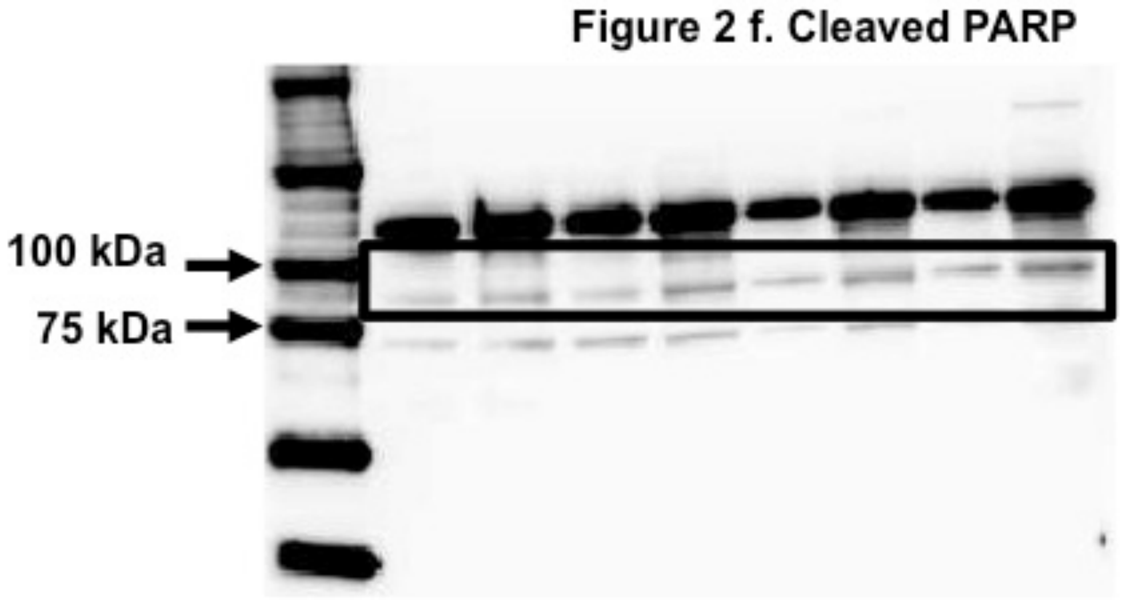

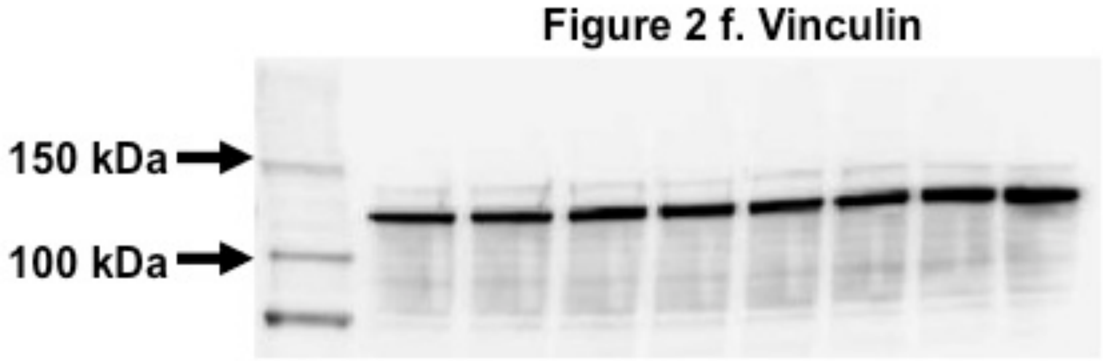

Uncropped western blotting images for Figure 2f.

Supplementary Figure 13.

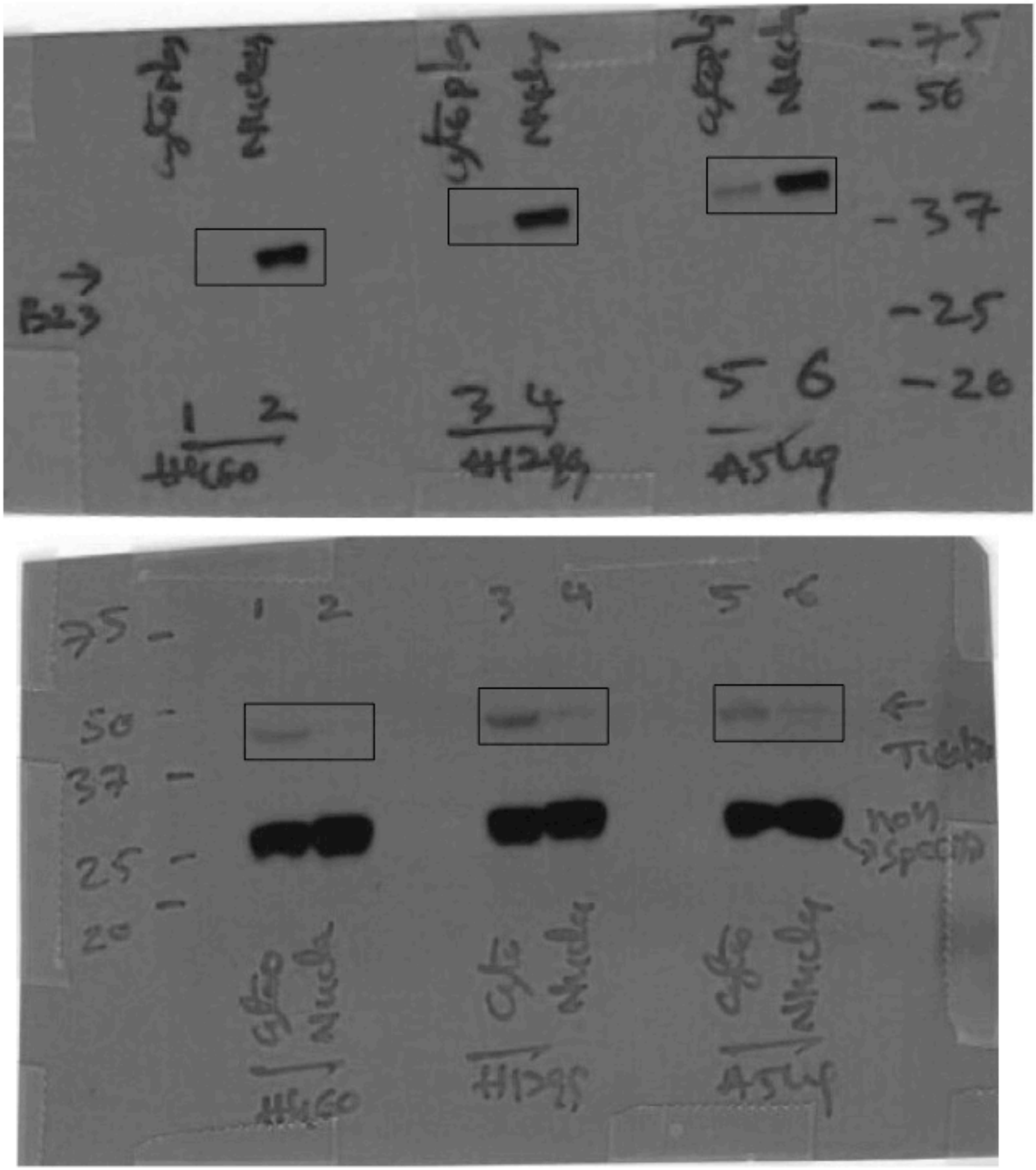

Uncropped western blotting images for Figure 4e

Supplementary Figure 14.

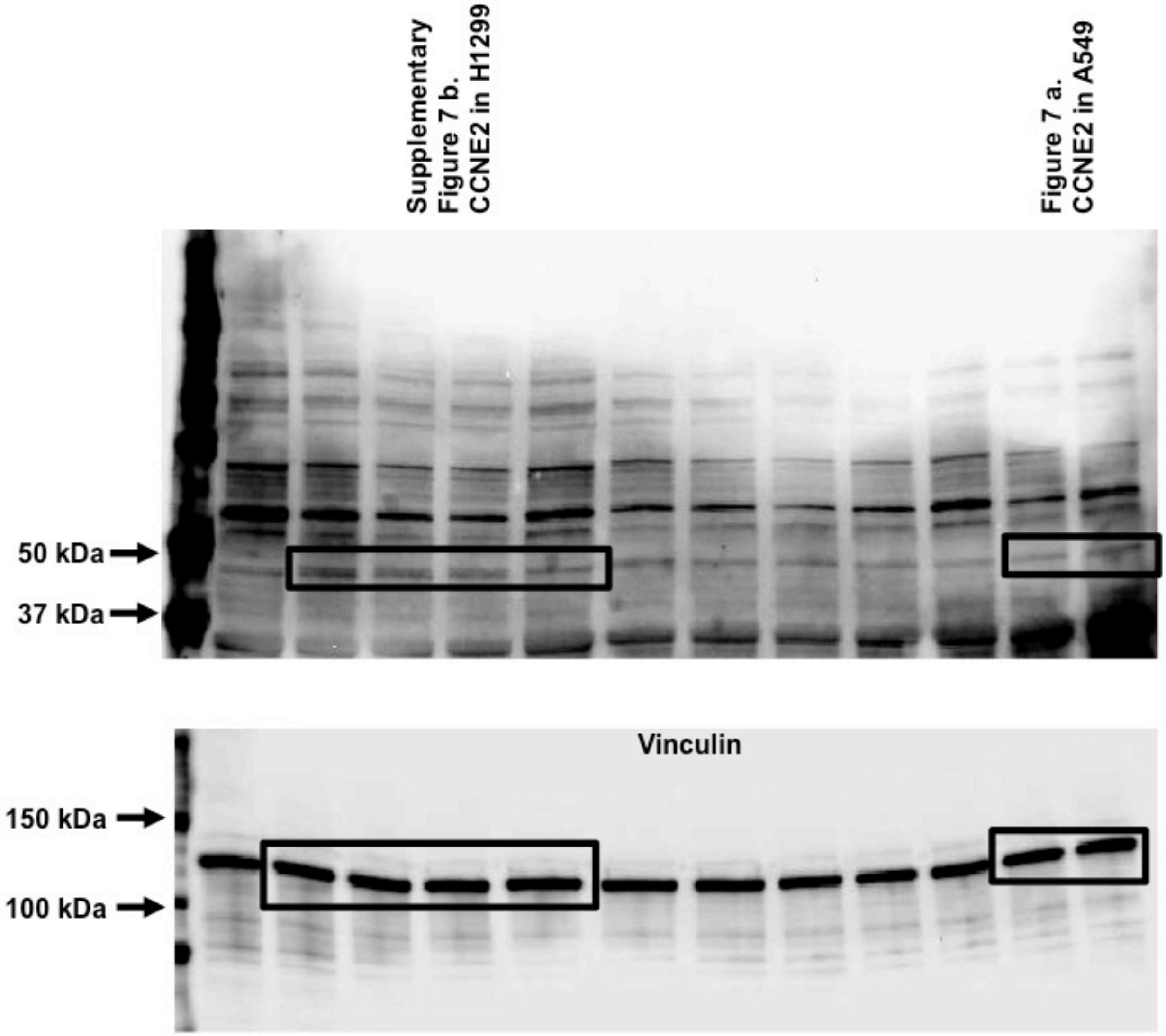

Uncropped western blotting images for Figure 7a and Supplementary Figure 7b.

Supplementary Figure 15.

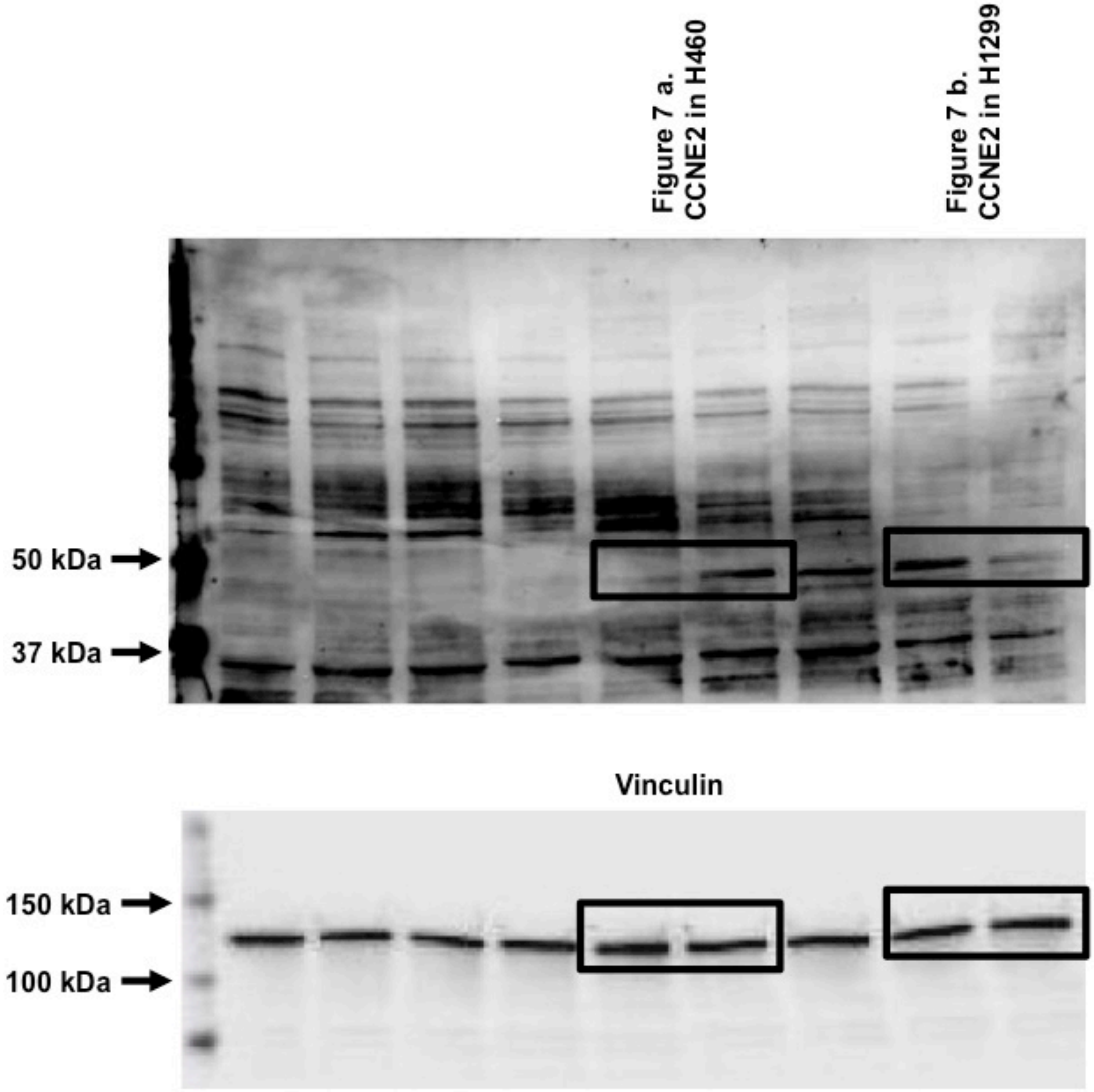

Uncropped western blotting images for Figure 7a and Figure 7b.

Supplementary Figure 16.

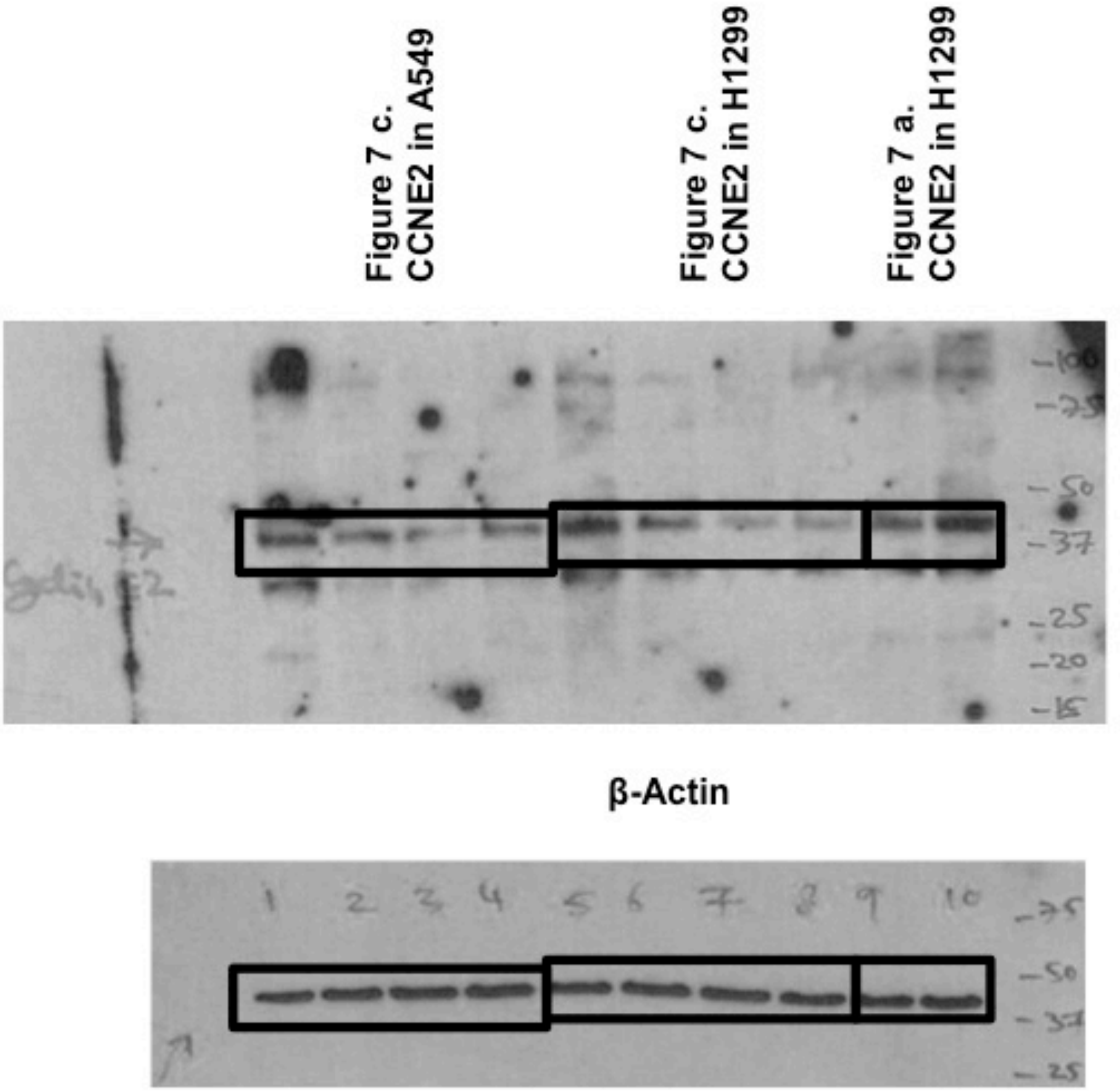

Uncropped western blotting images for Figure 7a and Figure 7c.

Supplementary Figure 17.

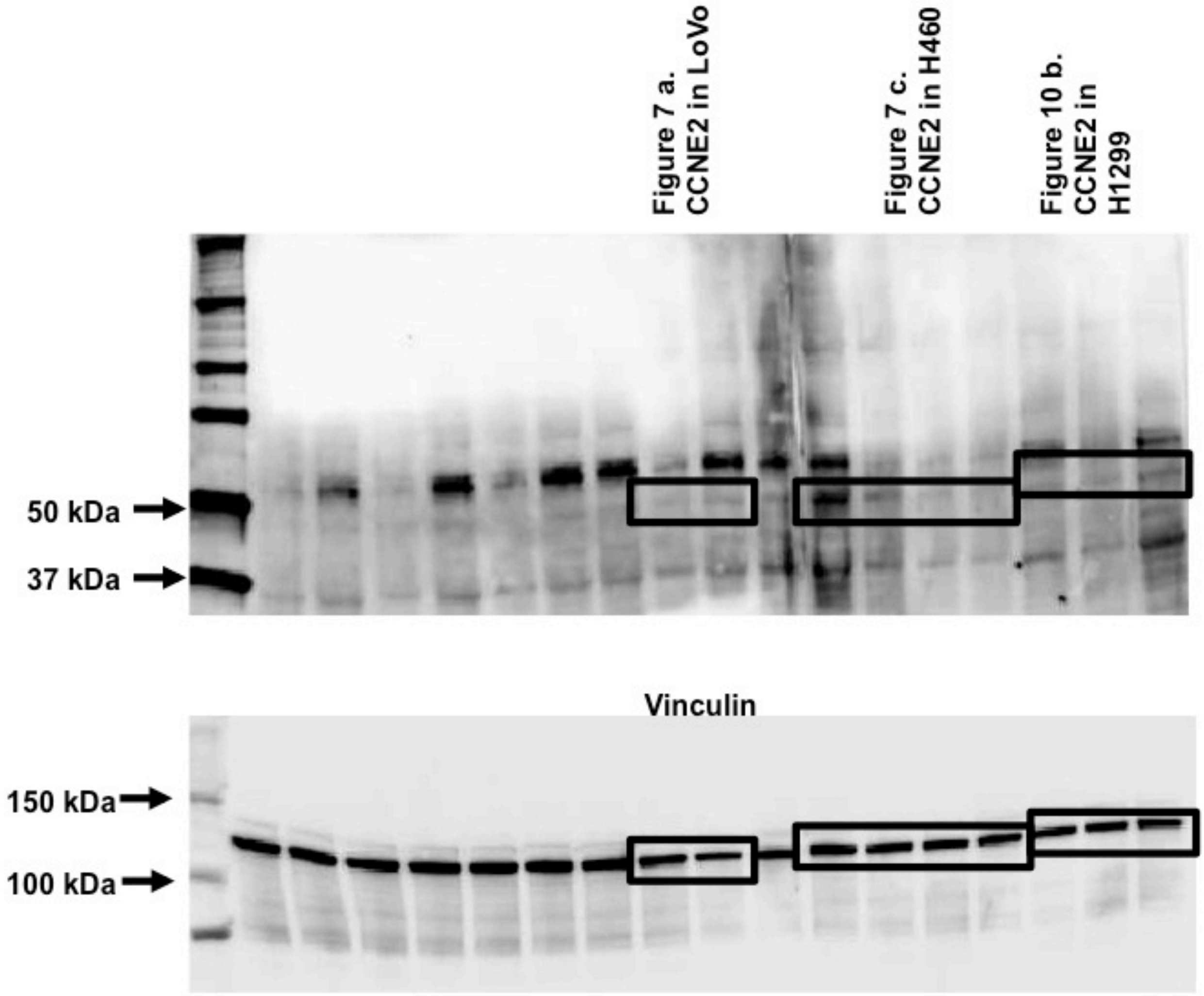

Uncropped western blotting images for Figure 7a, Figure 7c, and Figure 10b.

Supplementary Figure 18.

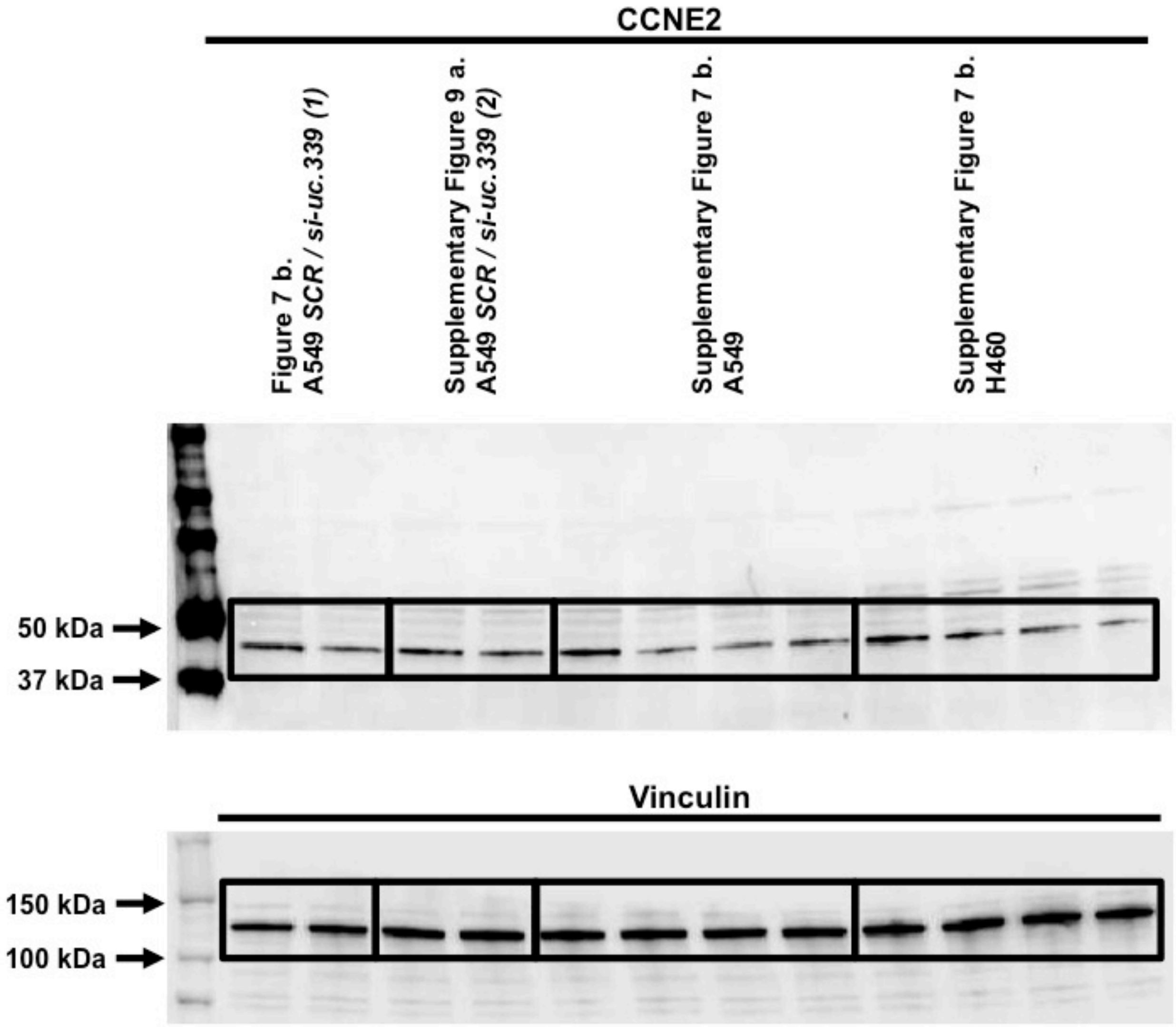

Uncropped western blotting images for Figure 7b, Supplementary Figure 7b and Supplementary Figure 9a.

Supplementary Figure 19.

Figure 7 b. CCNE2 in H460

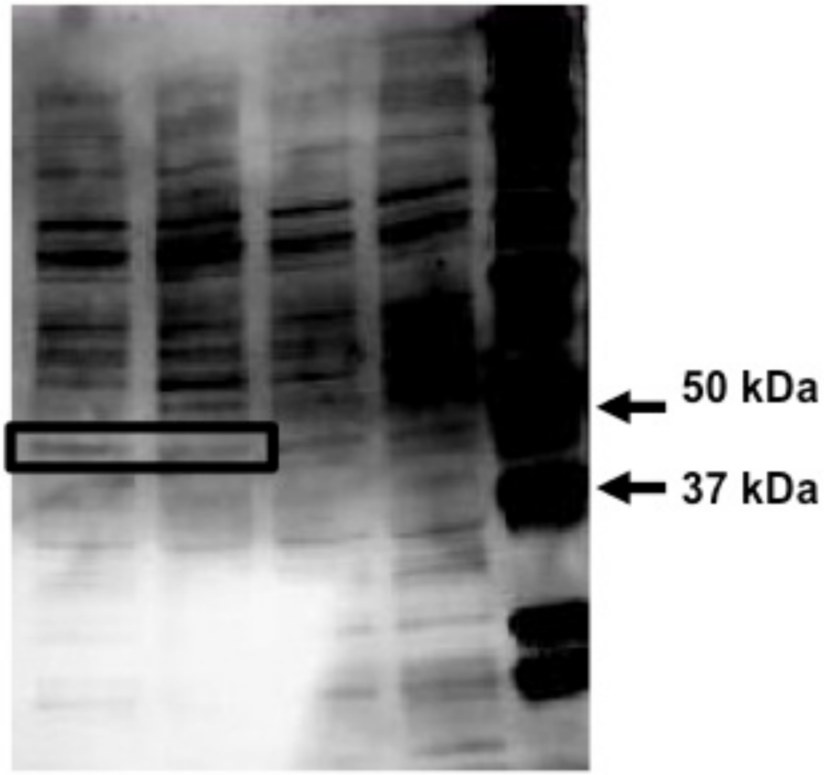

Figure 7 b. Vinculin in H460

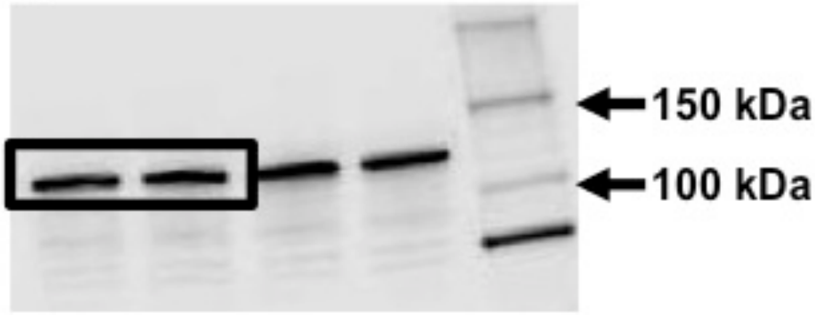

Uncropped western blotting images for Figure 7b.

Supplementary Figure 20.

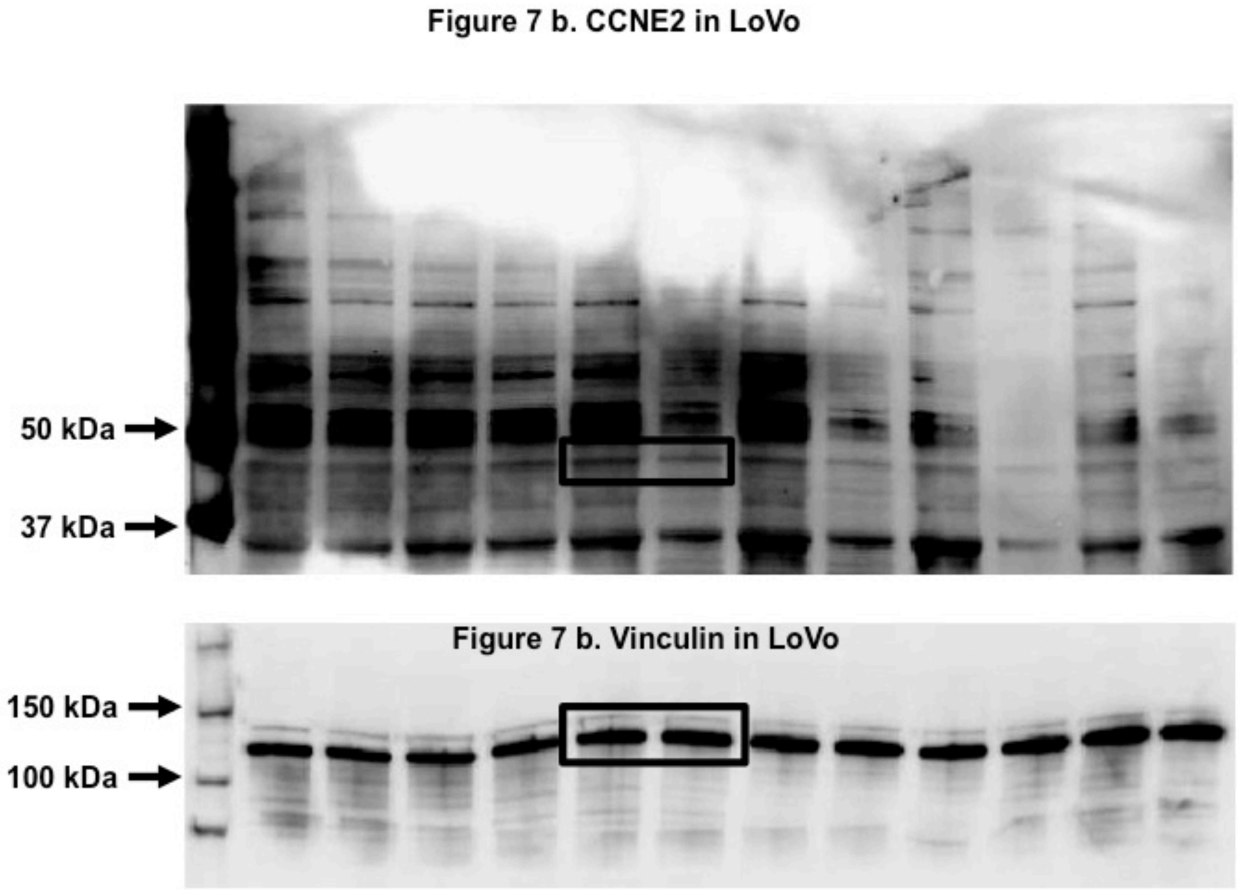

Uncropped western blotting images for Figure 7b.

Supplementary Figure 21.

Figure 7 c. CCNE2 in LoVo

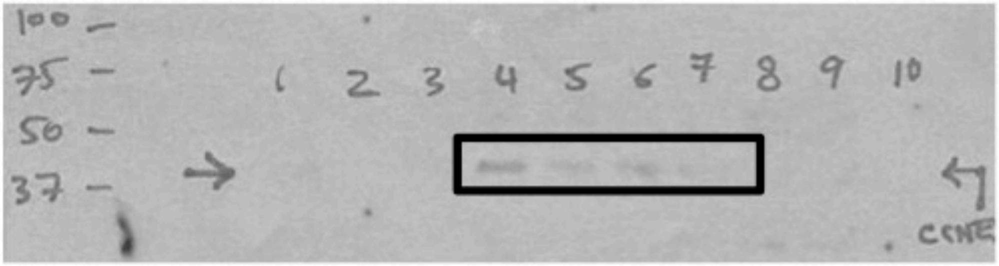

β-Actin

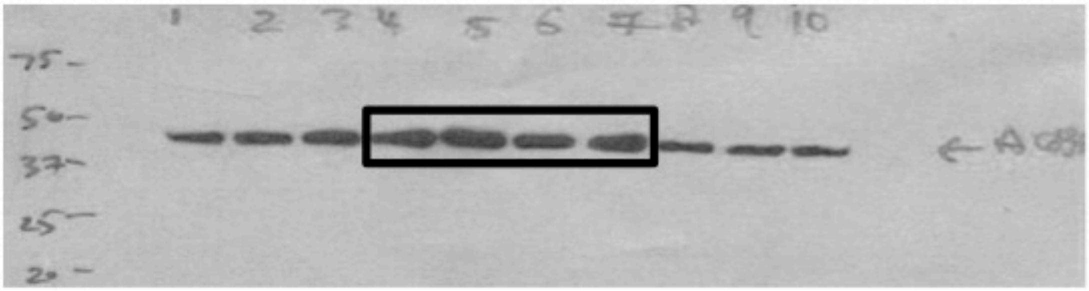

Uncropped western blotting images for Figure 7c.

Supplementary Figure 22.

Figure 7 e. CCNE2

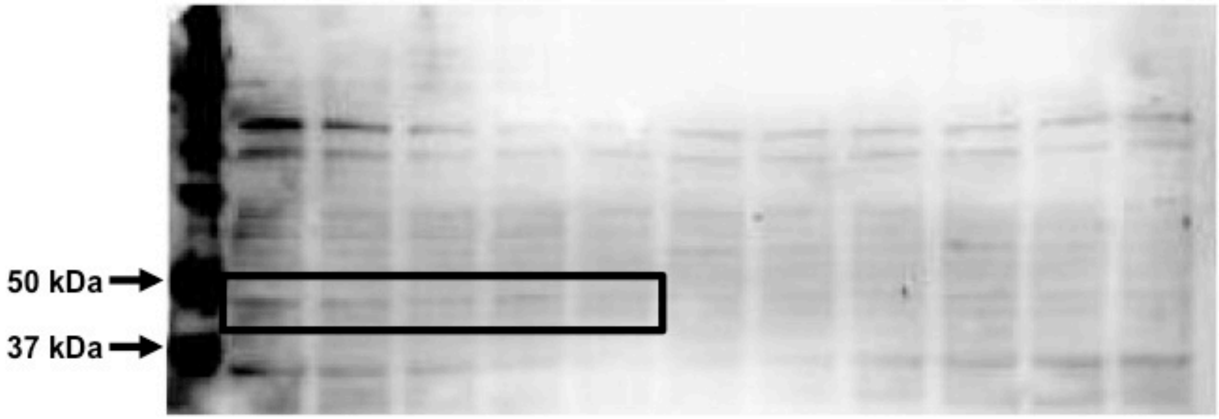

Figure 7 e. Vinculin

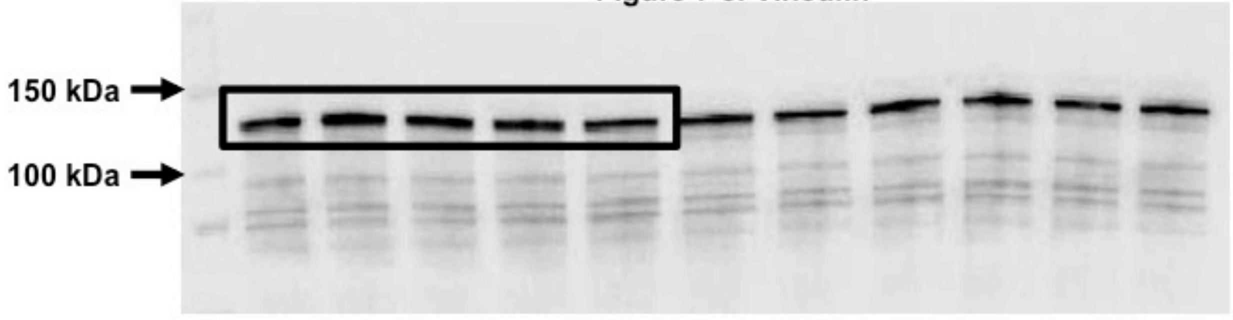

Uncropped western blotting images for Figure 7e.

Supplementary Figure 23.

Figure 7 f. CCNE2

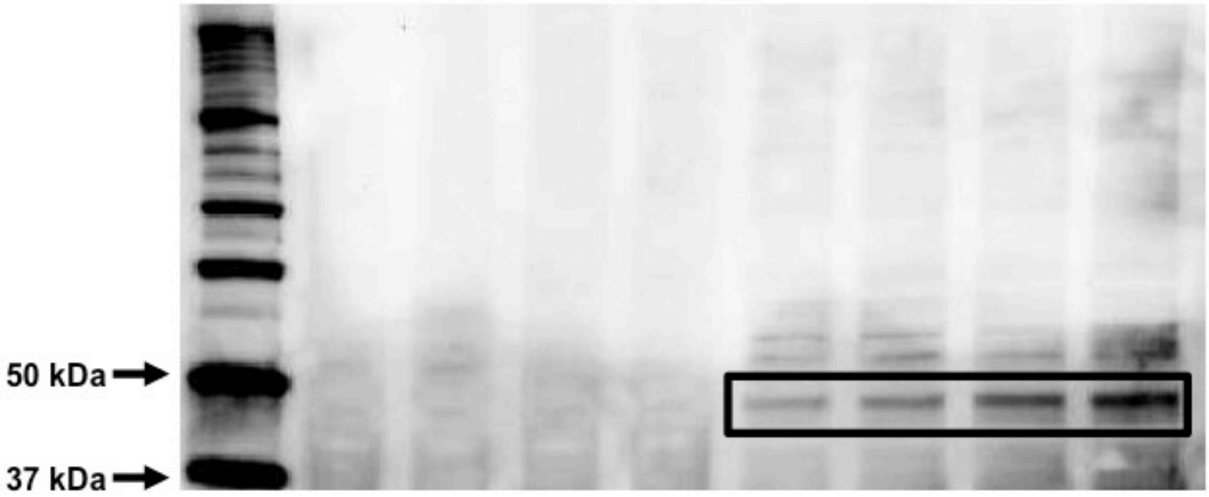

Figure 7 f. Vinculin

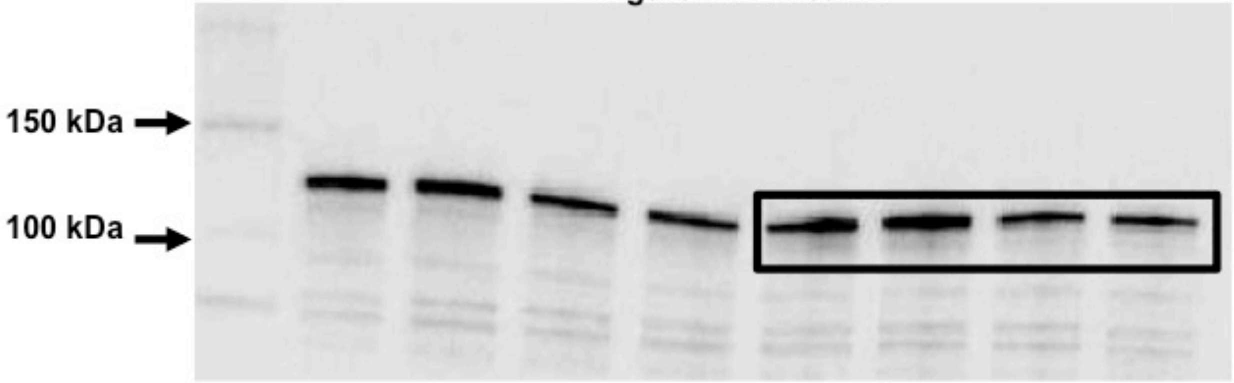

Uncropped western blotting images for Figure 7f.

Supplementary Figure 24.

Figure 7 g. CCNE2

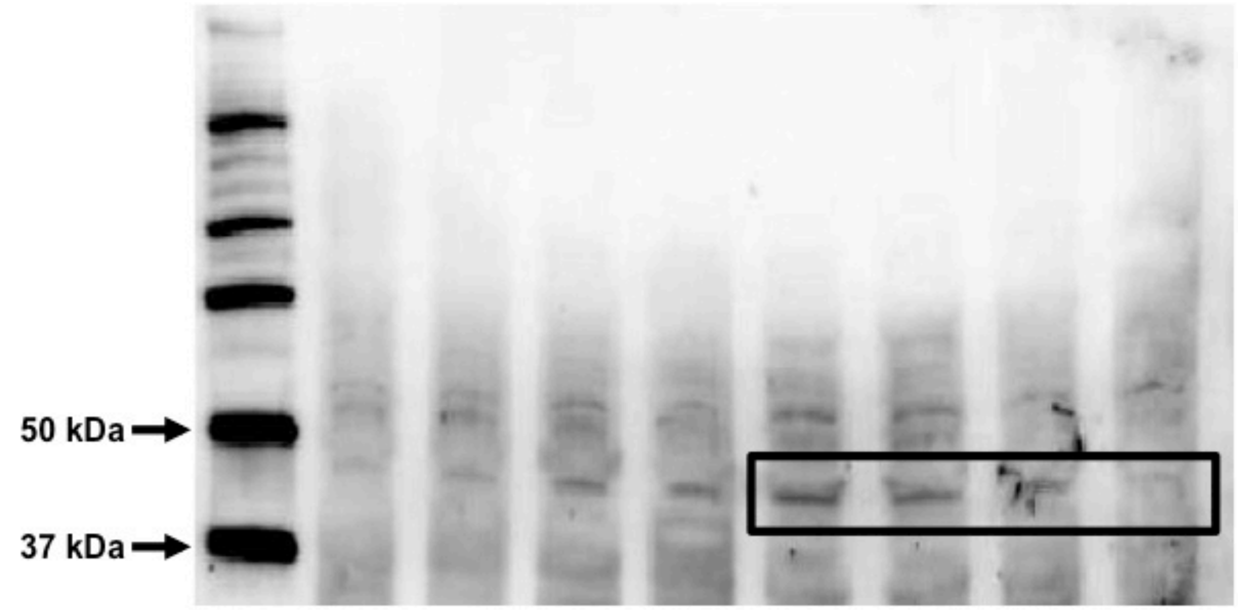

Figure 7 g. Vinculin

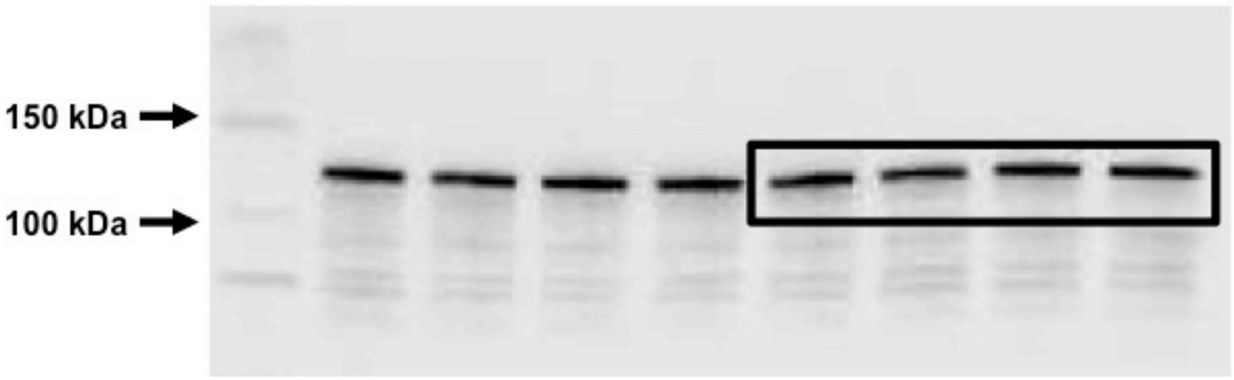

Uncropped western blotting images for Figure 7g.

Supplementary Figure 25.

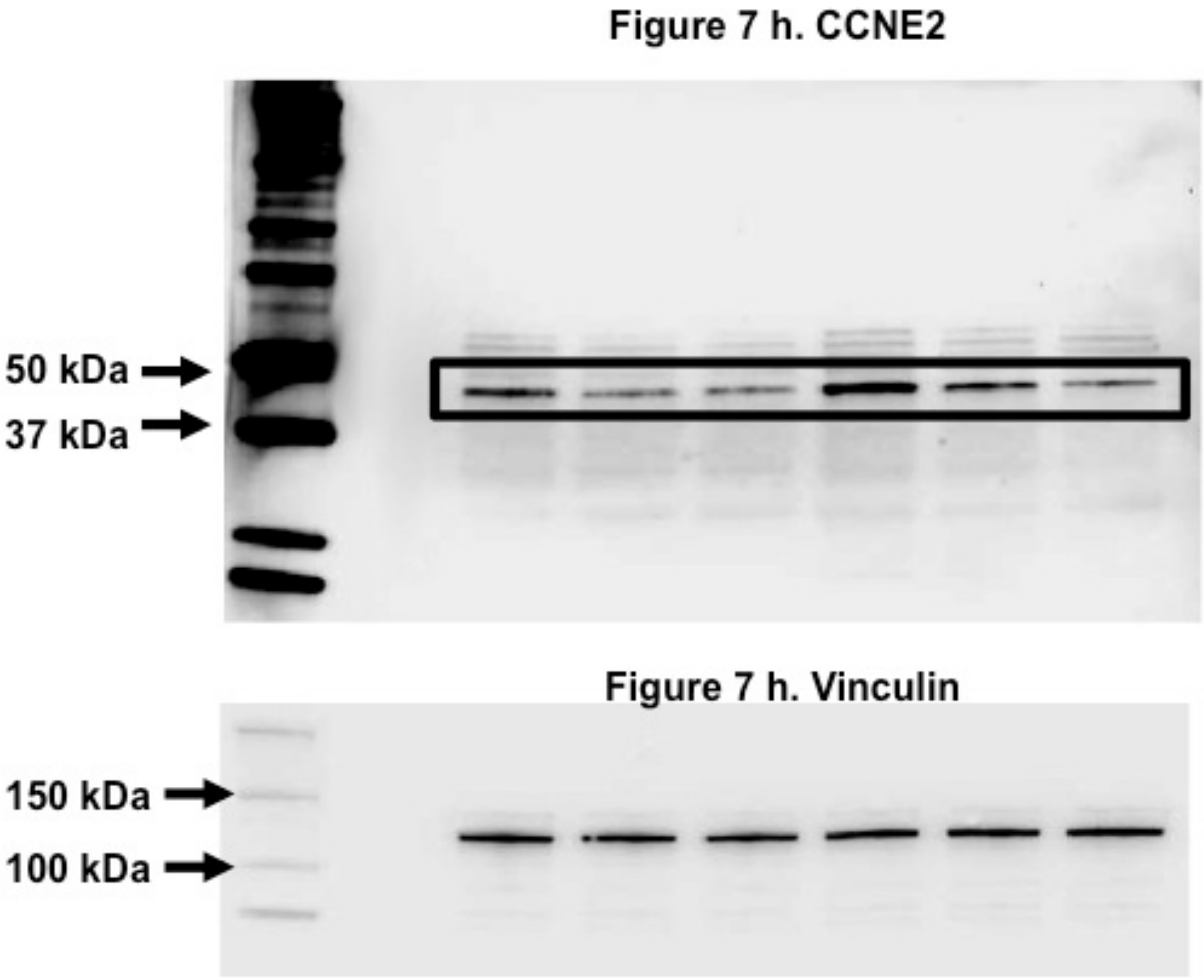

Uncropped western blotting images for Figure 7h.

Supplementary Figure 26.

Figure 10 a. CCNE2

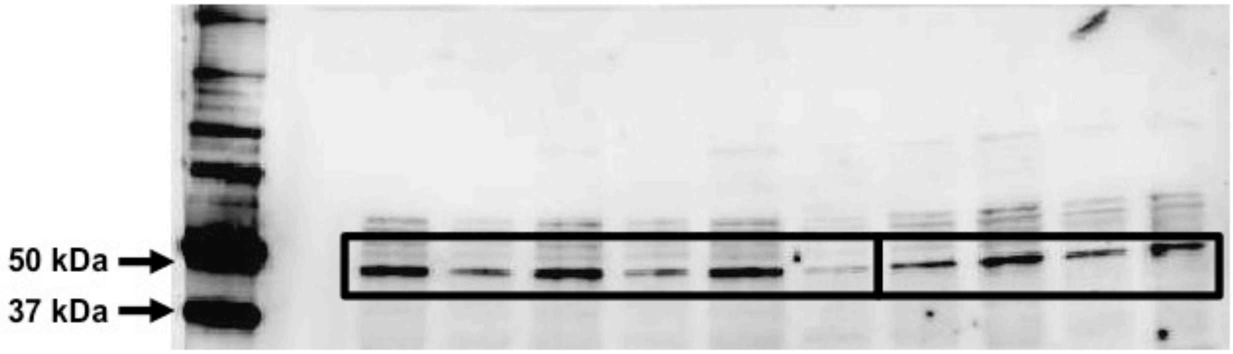

Figure 10 a. Vinculin

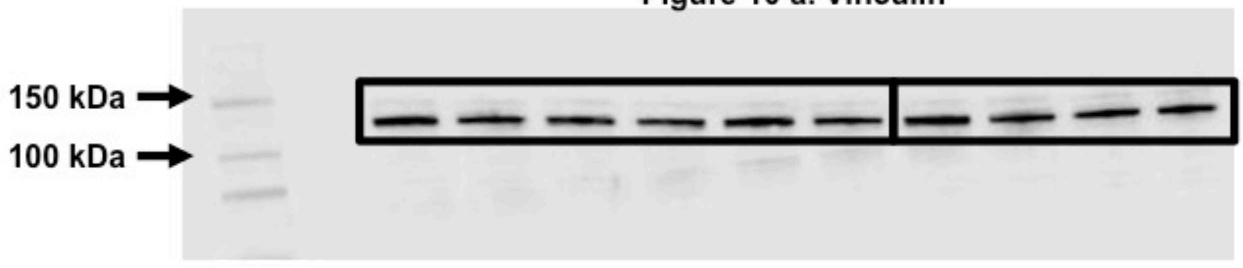

Uncropped western blotting images for Figure 10a.

Supplementary Figure 27.

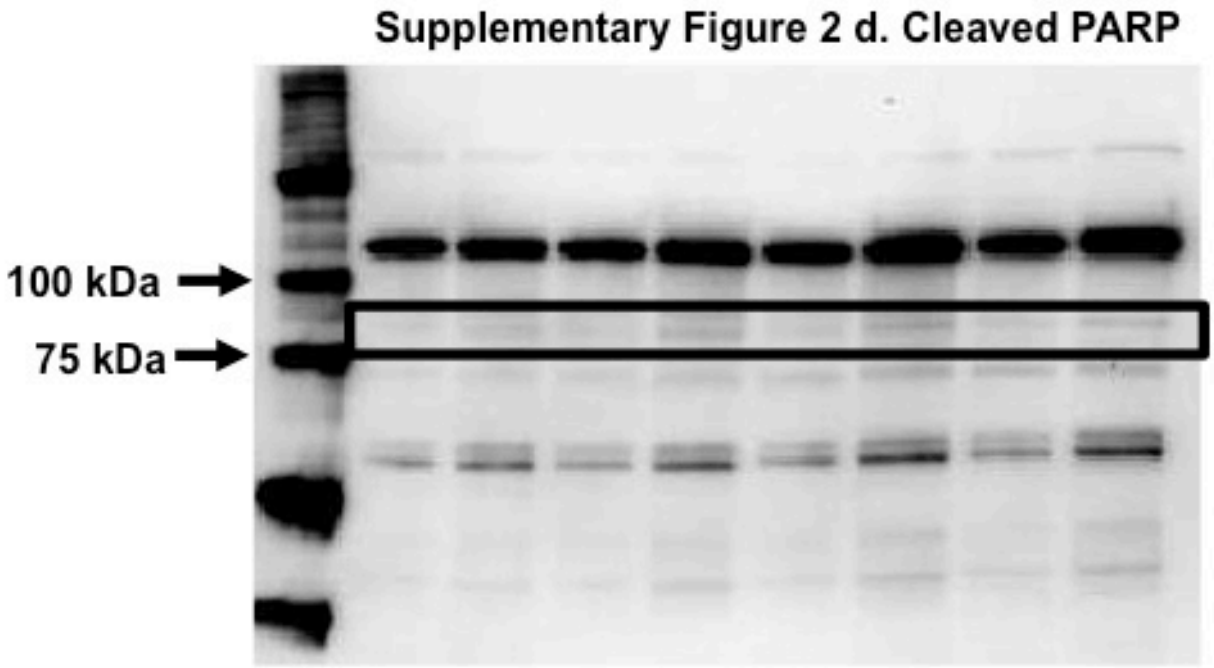

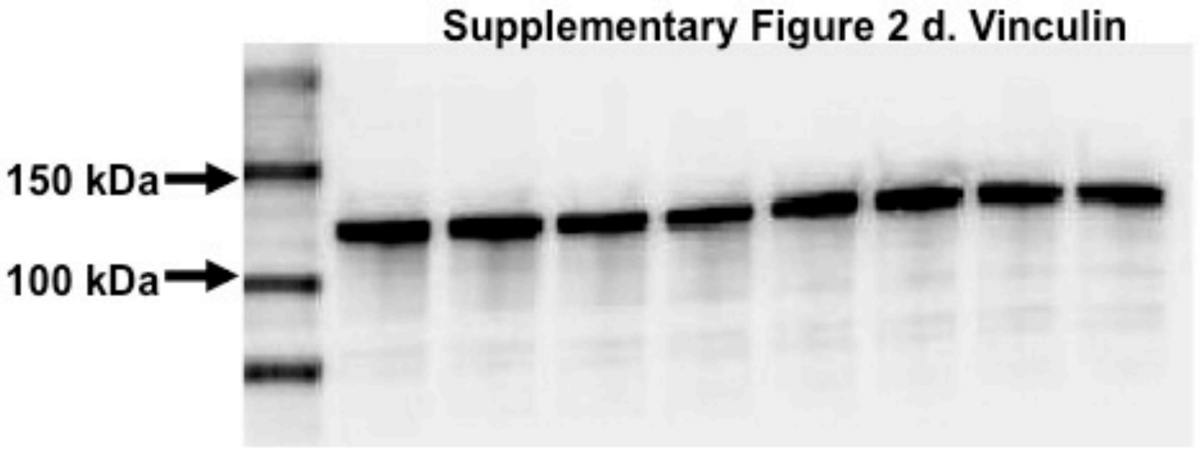

Uncropped western blotting images for Supplementary Figure 2d.

Supplementary Figure 28.

Supplementary Figure 5 b. ATP5G2

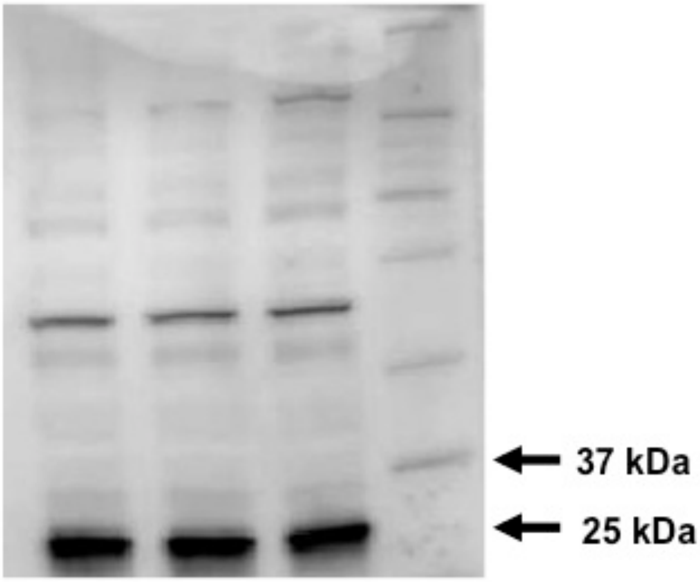

Supplementary Figure 5 b. Vinculin

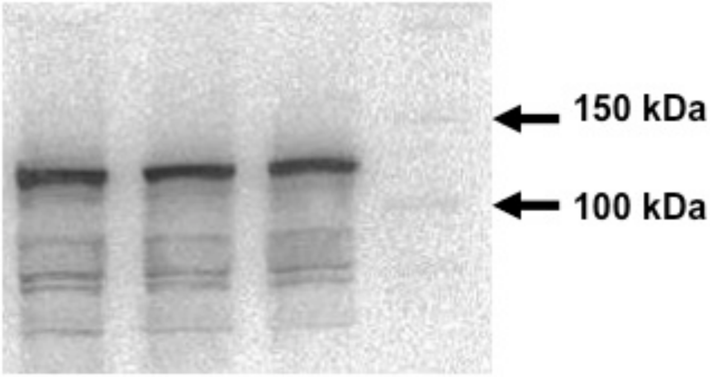

Uncropped western blotting images for Supplementary Figure 5b.

Supplementary Figure 29.

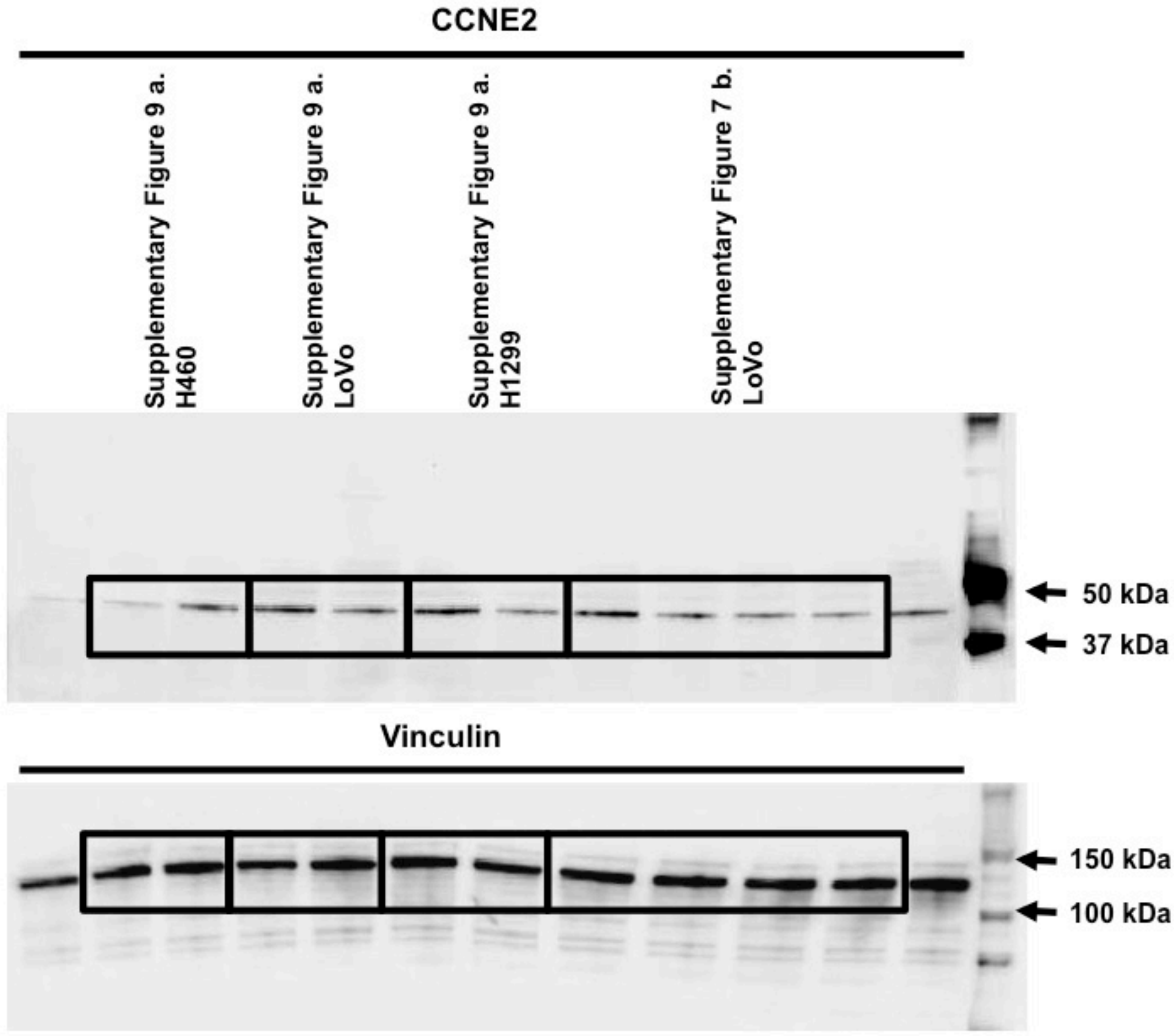

Uncropped western blotting images for Supplementary Figure 7b and Supplementary Figure 9a.

Supplementary Figure 30.

Supplementary Figure 9 b. CCNE2

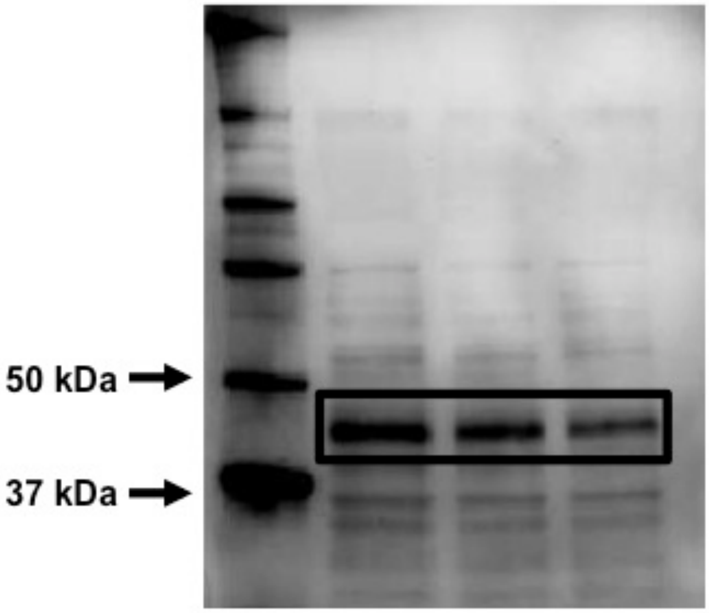

Supplementary Figure 9 b. Vinculin

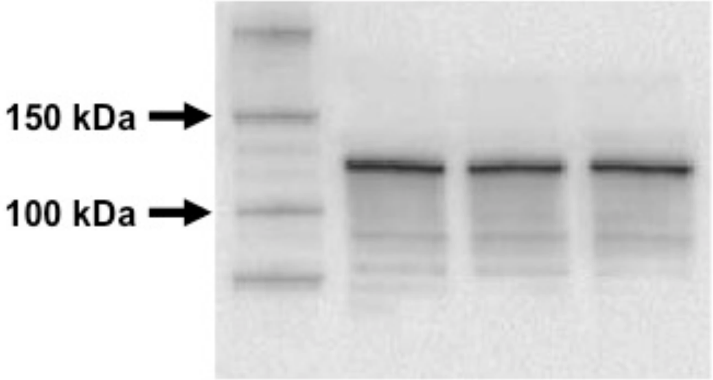

Uncropped western blotting images for Supplementary Figure 9b.

Supplementary Figure 31.

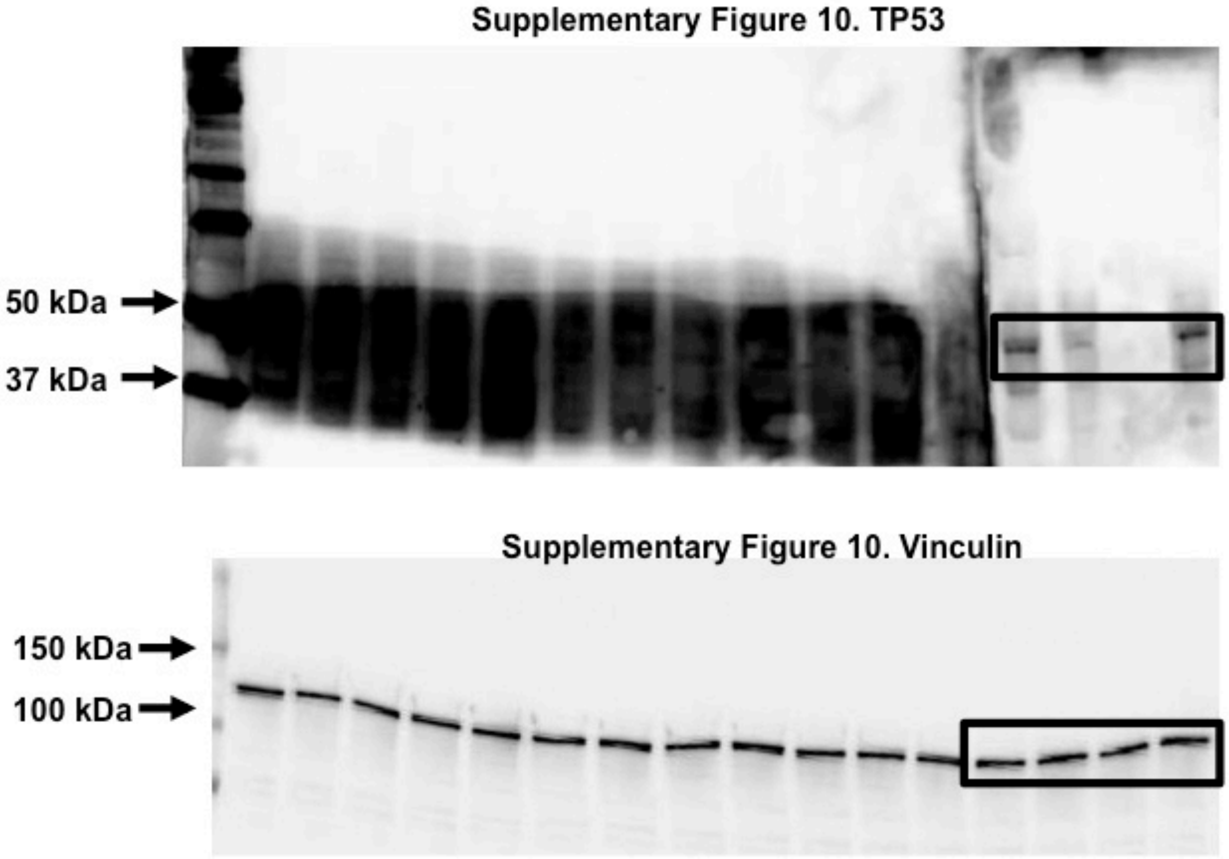

Uncropped western blotting images for Supplementary Figure 10.

Supplementary Figure 32.

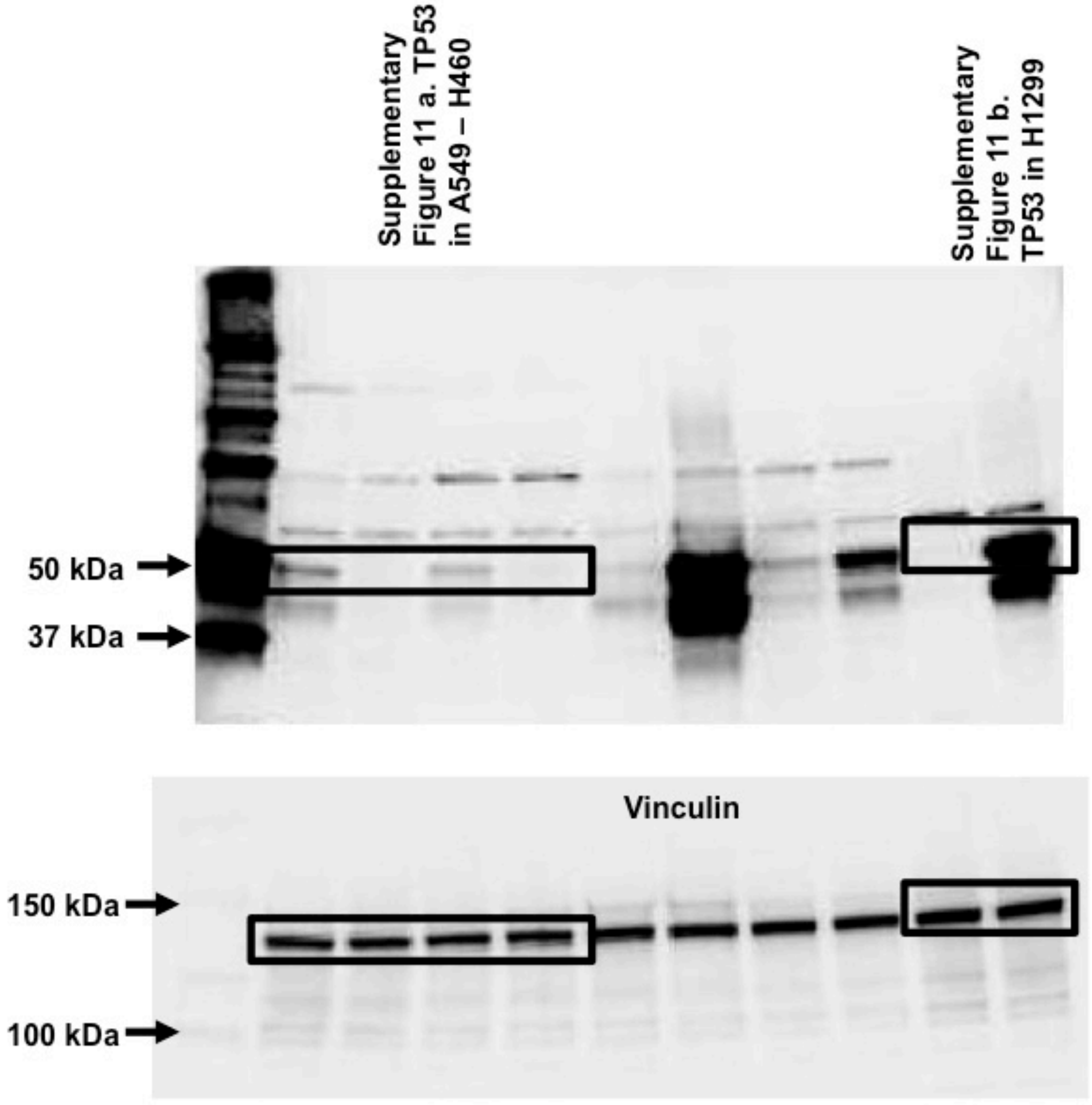

Uncropped western blotting images for Supplementary Figure 11a and Supplementary Figure 11b.

Supplementary Figure 33.

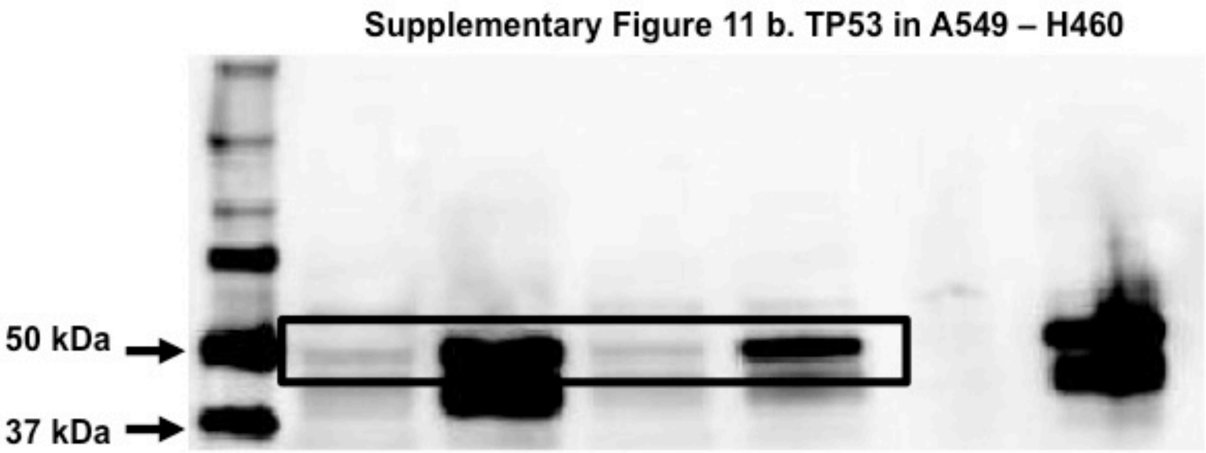

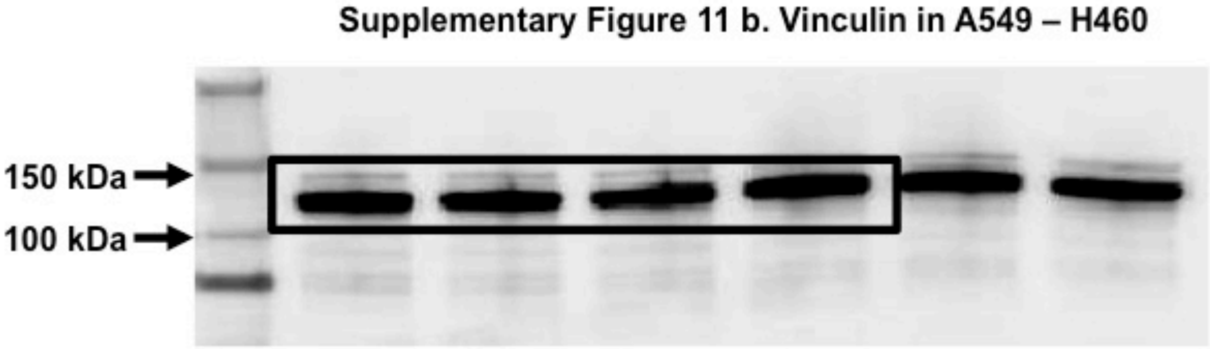

Uncropped western blotting images for Supplementary Figure 11b.

Supplementary Table 1. List of primers used in this study.

Procedures	Primer name	Sequence
Sequencing	F2	5'-CAGCCATTCTTTTCCTGCTC-3'
Sequencing	R11	5'-AAGTGGGCCCCTACCTAGAA-3'
Sequencing	5F	5'-CTCTTCCTGCAGTACTCCCCTGC-3'
Sequencing	5R	5'-GCCCCAGCTGCTCACCATCGCTA-3'
Sequencing	6F	5'-GATTGCTCTTAGGTCTGGCCCCTC-3'
Sequencing	6R	5'-GGCCACTGACAACCACCCTTAACC-3'
Sequencing	7F	5'-GTGTTGTCTCCTAGGTTGGCTCTG-3'
Sequencing	7R	5'-CAAGTGGCTCCTGACCTGGAGTC-3'
Sequencing	8F	5'-ACCTGATTTCCTTACTGCCTCTGGC-3'
Sequencing	8R	5'-GTCCTGCTTGCTTACCTCGCTTAGT-3'
uc.339 RT	RT *uc.339* reverse	5'- CTCCACAGTGCCTGGCACCAC-3'
uc.339 qPCR	AJX0Z39	Applied Biosystems (Life Technology) proprietary sequence of custom-made primers
RACE	Gene specific F	5'-CATTTTTATGGCCCTGAGCT-3'
RACE	Gene specific R	5'-CTTCTCGCCCGCTTCTCC-3'
RACE	Nested gene specific F	5'-GGAGAAGCGGGCGAGAAG-3'
RACE	Nested gene specific R	5'-AGCTCAGGGCCATAAAAATG-3'
Cloning of *uc.339* in pCDH-CMV-MCS-EF1-copGFP vector	*uc.339* F	5'- GCGAATTCACGCAGCACGAGAAAGACG - 3'
Cloning of *uc.339* in pCDH-CMV-MCS-EF1-copGFP vector	*uc.339* R	5'- TCGCGGCCGCTTTTGGTGGGAATGGA -3'
Mutagenesis of *miR-339-3p* CS in *uc.339*	Del. *miR-339-3p uc.339* CS F	5'- GTCCTTCCCGCCGCCCGCGGCGCCC -3'
Mutagenesis of *miR-339-3p* CS in *uc.339*	Del. *miR-339-3p uc.339* CS R	5'- GGGCGCCGCGGGCGGCGGGAAGGAC -3'

Mutagenesis of *miR-663b-3p* CS in *uc.339*	Del. *miR-663b-3p uc.339* CS F	5’- CAGACAGGCACACCCGATAAAAACCGCCGC -3’
Mutagenesis of *miR-663b-3p* CS in *uc.339*	Del. *miR-663b-3p uc.339* CS R	5’- GCGGCGGTTTTTATCGGGTGTGCCTGTCTG -3’
Mutagenesis of *miR-95-5p* CS in *uc.339*	Del. *miR-95-5p uc.339* CS R	5’- CACCACTTGGTACTTGTTGATTCCCAAAGATC -3’
Mutagenesis of *miR-339-3p* CS in CCNE2 3’ UTR	Del. *miR-339-3p* CCNE2 CS F	5'-TTTGCCTTGCCATAACACATTTTTTAACTAATAACCTGTGCTCTAAACAG-3'
Mutagenesis of *miR-339-3p* CS in CCNE2 3’ UTR	Del. *miR-339-3p* CCNE2 CS R	5'-CTGTTTAGAGCACAGGTTATTAGTTAAAAAATGTGTTATGGCAAGGCAAA-3'
Mutagenesis of *miR-663b-3p* CS in CCNE2 3’ UTR	Del. *miR-663b-3p* CCNE2 CS F	5'-TAAATGCTGTGGCTCCTTCCTATTTTGTATATCACAATTTGGGTG-3'
Mutagenesis of *miR-663b-3p* CS in CCNE2 3’ UTR	Del. *miR-663b-3p* CCNE2 CS R	5'-CACCCAAATTGTGATATACAAAATAGGAAGGAGCCACAGCATTTA-3'
Mutagenesis of *miR-95-5p* CS in CCNE2 3’ UTR	Del. *miR-95-5p* CCNE2 CS F	5’-CTAGATTGCTAGTTTATTTTCTCTTCTCCCTTTGAAGAAAC- 3’
Mutagenesis of *miR-95-5p* CS in CCNE2 3’ UTR	Del. *miR-95-5p* CCNE2 CS R	5’-GTTTCTTCAAAGGGAGAAGAGAAAATAAACTAGCAATCTAG 3’
Cloning of *TP53* CS 1 in pGL4.23(luc2/minP)	CS1 F	5’- CAGGCATGCGCCACCATGCCCG-3’
Cloning of *TP53* CS 1 in pGL4.23(luc2/minP)	CS1 R	5’-CTAGCGGGCATGGTGGCGCATGCCTGGTAC-3’
Cloning of *TP53* CS 2 in pGL4.23(luc2/minP)	CS2 F	5’-CCTCCTTGTCTCCCAGACAGGACCTGCCCG-3’
Cloning of *TP53* CS 2 in pGL4.23(luc2/minP)	CS2 R	5’-CTAGCGGGCAGGTCCTGTCTGGGAGACAAGGAGGGTAC-3’
Cloning of *TP53* CS 3 in pGL4.23(luc2/minP)	CS3 F	5’-CGGACTTGCGTCCCCTTTCCGAGCATGCGCG-3’
Cloning of *TP53* CS 3 in pGL4.23(luc2/minP)	CS3 R	5’-CTAGCGCGCATGCTCGGAAAGGGGACGCAAGTCCGGTAC-3’
Cloning of *TP53* CS 4 in pGL4.23(luc2/minP)	CS4 F	5’-CGGTCAAGATCTCTGGGGGAGCTAGGTTG-3’
Cloning of *TP53* CS 4 in pGL4.23(luc2/minP)	CS4 R	5’-CTAGCAACCTAGCTCCCCCAGAGATCTTGACCGGTAC-3’
uc.339 precipitation	3’ biotinylated RNA	5’-UACCUGUUGAUUGAUUCCCAA-3’.

SUPPLEMENTARY REFERENCES

1. Bejerano G*, et al.* Ultraconserved elements in the human genome. *Science* **304**, 1321-1325 (2004).
